# Sodium Tetraazidoaurate(III)—From Na[AuCl_4_]·2H_2_O to Na[Au(N_3_)_4_] and Beyond One Step at a Time

**DOI:** 10.1002/chem.202502800

**Published:** 2026-03-26

**Authors:** Mehmet Somer, Joannis Psilitelis, Raul Cardoso‐Gil, Thomas Doert, Franziska Jach, Ayberk Yılmaz, Alexander Ovchinnikov, Yurii Prots, Helge Rosner, Marcus P. Schmidt, Peter Höhn

**Affiliations:** ^1^ Chemistry Department Koç University Sariyer‐İstanbul Türkiye; ^2^ Eberhard‐Karls‐Universität Tübingen Teufen Switzerland; ^3^ Chemische Metallkunde Max‐Planck‐Institut für Chemische Physik fester Stoffe Dresden Germany; ^4^ Faculty of Chemistry and Food Chemistry TUD Dresden University of Technology Dresden Germany; ^5^ Department Energy Materials and Test Devices Fraunhofer Institute for Integrated Systems and Device Technology IISB Erlangen Germany; ^6^ Fen Fakültesi Fizik Bölümü İstanbul Üniversitesi İstanbul Türkiye; ^7^ Physik der Quantenmaterie Max‐Planck‐Institut für Chemische Physik fester Stoffe Dresden Germany

**Keywords:** azide, crystal structure, gold, thermal analysis, vibration spectroscopy

## Abstract

The novel sodium tetrachlorido‐/azidoaurate(III) dihydrates Na[AuCl_4–_ *_x_* (N_3_)*_x_*]·2H_2_O (*x* = 0, 1, 2, 3, 4) provide the first example of a complete series of gradual substitution on square planar complex anions to be described. Transparent yellow to dark orange single crystals of these phases were synthesized by reaction of NaN_3_ and AuCl_3_ or HAuCl_4_ in different molar ratios from aqueous solution. Controlled dehydration of Na[Au(N_3_)_4_]·2H_2_O led to Na[Au(N_3_)_4_]·H_2_O and Na[Au(N_3_)_4_] in form of orange microcrystalline powders, the latter being a highly explosive material. Predominant structural features of all phases are discrete anions [AuCl_4–_ *_x_* (N_3_)*_x_*]^–^ with gold in an ordered square planar coordination of azide/chloride anions. Vibrational spectra show good agreement with other known azidoaurates(III).

## Graphical abstract

Novel sodium chlorido‐/azidoaurate(III) dihydrates Na[AuCl_4–_ *_x_* (N_3_)*_x_*]·2H_2_O (*x* = 0, 1, 2, 3, 4) provide the first example of a complete series of gradual substitution on tetramer complex anions to be described. Controlled dehydration of Na[Au(N_3_)_4_]·2H_2_O leads to Na[Au(N_3_)_4_]·H_2_O and Na[Au(N_3_)_4_], the latter being a highly explosive material.

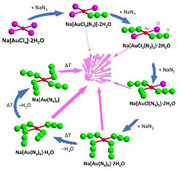

## 1 Introduction

Inorganic azides receive great attention due to their explosive nature; their shock and friction sensitivity, as energetic materials; and finally, as starting materials for the synthesis of both nitrides and nitridometalates [1, 2, 3, 4, 5, 6, 7]. Our recent interest into azidometalates was rekindled in testing them as a novel synthetic route toward nitridoaurates and gold nitrides.

The binary azides of alkali metals such as LiN_3_, NaN_3_, KN_3_, RbN_3_ and CsN_3_ [8, 9], like the heavy alkaline‐earth metal azides *Ae* (N_3_)_2_ (*Ae* = Ca, Sr, Ba) [10, 11, 12], contain linear and (nearly) symmetrical azide species. In these phases, coordination numbers of cations by azide range from 6 (LiN_3_) [9] to 8 (KN_3_ [13], Sr(N_3_)_2_ [14, 15]) and 9 (Ba(N_3_)_2_) [14, 16, 17].

Binary gold azides are hitherto unknown. First reports dating to 1898 [18] were shown to be in reality a ternary sodium azidoaurate(III) in 1957 [19]. The crystal structures and vibrational properties as well as topology and bonding of ternary azidoaurates(III) *A*[Au(N_3_)_4_] with monovalent metal ions (*A =* K, Rb, Cs) [20] or complex cations (*A =* [As(C_6_H_5_)_4_] [21], [N(CH_3_)_4_] [22]) have been investigated in more detail. The explosive nature of the azidoaurates has been shown to increase with decreasing cationic radius [23]. Although binary azides of the alkaline‐earth elements were extensively investigated [4], the only alkaline‐earth gold azide known up to now is Ba[Au(N_3_)_4_]_2_·4H_2_O [24], which may be handled safely but explodes as soon as crystal water is removed.

All azidoaurate(III) anions [Au(N_3_)_4_]^–^ feature square‐planar coordination of Au by N with azide species displaying a N–N–N bond angle of 172 ± 3° and two different N–N bond lengths [25]; however, they show structural variety besides the ubiquitous “windmill” pattern.

In this study, we present the phases Na[AuCl_4–_ *_x_* (N_3_)*_x_*]·2H_2_O (*x* = 0, 1, 2, 3, 4). This series is also the first example of the complete evolution of ordered phases in a system *ABX*_4_ ↔ *ABY*_4_ for *any* combination of cations *A*, *B* or anions *X*, *Y* as well as the stepwise dehydration of Na[Au(N_3_)_4_]·*n*H_2_O (*n* = 2, 1, 0).

***Explicit Safety Hazard!*** Whereas all hydrated phases Na[AuCl_4–_ *_x_* (N_3_)*_x_*]·2H_2_O (*x* = 1, 2, 3, 4) may be handled safely in humid conditions, upon drying or heating they show a tendency to explode on various occasions and are therefore extremely dangerous materials. Experiments to obtain samples free of crystal water resulted in even more sensitive materials and frequently ended in heavy explosions.

## 2 Results and Discussion

### 2.1 Crystal Structures of the Sodium Tetrachlorido/Azidoaurate Dihydrates

It was possible to identify seven different phases in the system Na[AuCl_4–_ *_x_* (N_3_)*_x_*]·*n*H_2_O (*x* = 0, 1, 2, 3, 4; *n* = 2, 1, 0), of which only Na[AuCl_4_]·2H_2_O has been published before [26]. Tables S1, S5 in the Supporting Information contain details of the crystal structure refinements and crystallographic data for all phases; atomic positions and corresponding displacement parameters are given in Tables S2a–e, S3a–e, S6a–b, S7b, respectively. Selected distances and angles are listed in Tables S4a–e, S8a–b.

Single crystals (Figure S1) of all new hydrated phases Na[AuCl_4–_ *_x_* (N_3_)*_x_*]·2H_2_O (*x* = 1, 2, 3, 4) are obtained by the reaction of NaN_3_ with either AuCl_3_ or HAuCl_4_ in defined ratios from aqueous solution; the (partially) dehydrated phases were prepared as microcrystalline powders by heating or cautious drying in a vacuum. Crystal structures and vibrational spectroscopic properties are reported in detail.

All phases obtained are relatively stable under ambient conditions in air and under laboratory light. During the course of our investigations spanning several years, samples Na[AuCl_4–_ *_x_* (N_3_)*_x_*] slowly decomposed to elemental Au and NaCl without any identifiable intermediate stages. In particular, no signs of the formation of Au(I) intermediate complexes were observed for any of the phases, in contrast to the light‐induced reduction of deep red [As(C_6_H_5_)_4_][Au(N_3_)_4_] to colorless [As(C_6_H_5_)_4_][Au(N_3_)_4_] in THF [27].

**FIGURE 1:**
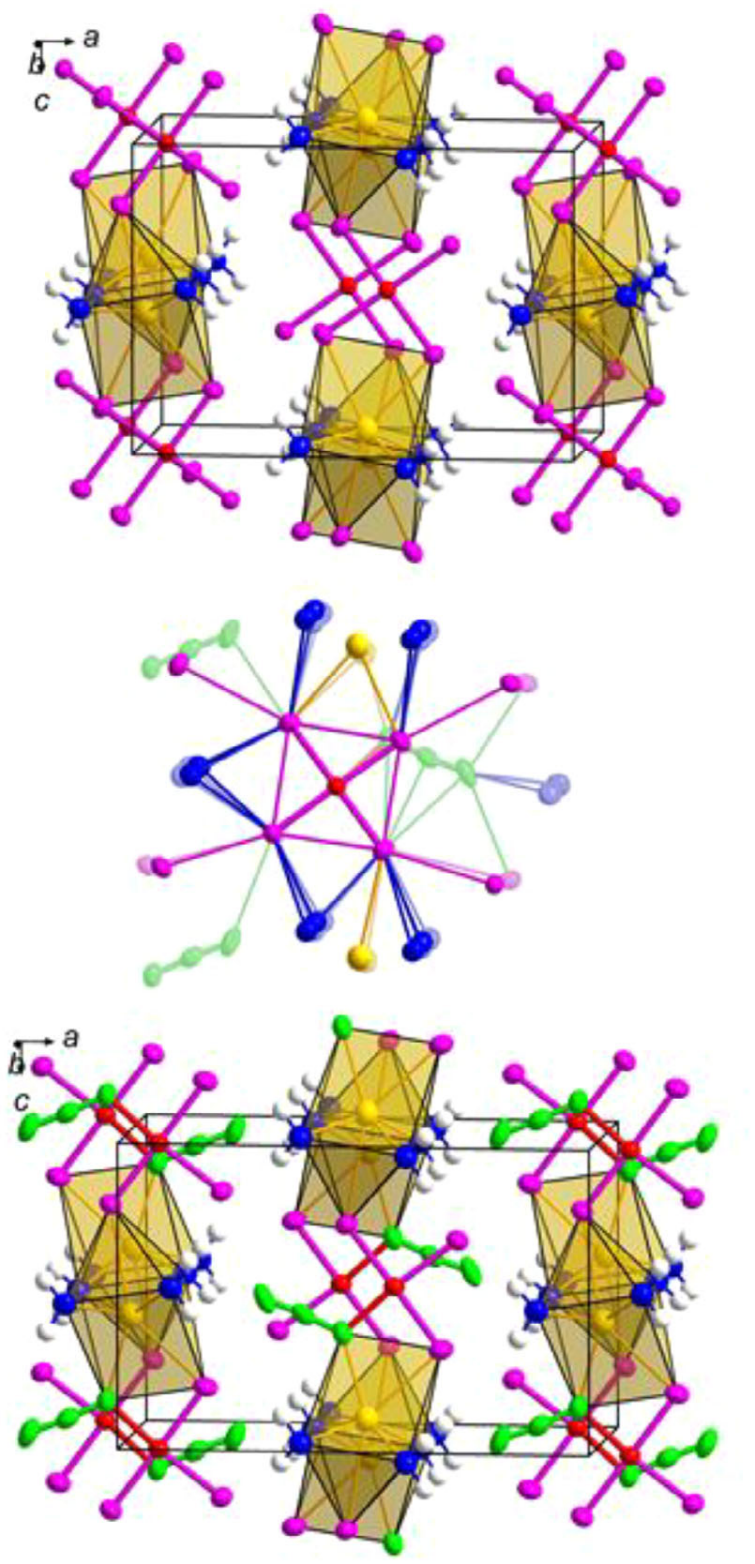
The crystal structures of Na[AuCl_4_]·2H_2_O (top) and Na[AuCl_3_(N_3_)]·2H_2_O (bottom); Au red, Na yellow, Cl pink, N green, O blue, H white. Details of the overlaid complex anions [AuCl_4_]^–^ (solid) and [AuCl_3_(N_3_)]^–^ (shaded) and their next nearest neighbors Na, Cl, O, and N (center) highlight the changes accommodated with the substitution Cl ↔ N_3_.

**Na[AuCl_4_]·2H_2_O** and **Na[AuCl_3_(N_3_)]·2H_2_O** (Figure 1 top and bottom) crystallize isotypic (considering N_3_ as one atom) in the orthorhombic space group *Pnma* (No. 62) with *Z* = 4 and form the endmembers of the continuous solution series Na[AuCl_4–_ *_x_* (N_3_)*_x_*]·2H_2_O (0 ≤ *x* ≤ 1, Figure S2). Only one of the four crystallographically independent Cl moieties in Na[AuCl_4_]·2H_2_O is exchanged by azide; upon full exchange of this position, no further exchange takes place on other positions in the same structure type, but the new phase Na[AuCl_2_(N_3_)_2_]·2H_2_O forms.

The predominant structural features in Na[AuCl_4_]·2H_2_O and Na[AuCl_3_(N_3_)]·2H_2_O are the complex anions [AuCl_4_]^–^/[AuCl_3_(N_3_)]^–^. The gold atom is located within a slightly distorted tetragonal planar coordination sphere built up by Cl and the *inner* N species of the azide ion, which is also located on this plane. These anions are stacked ···*AB*··· along [010] with *d*(Au–Au) = 3.8774(2) Å / 3.9912(3) Å and  ∠(Au–Au–Au) = 131.26(1)° / 119.80(1)° in Na[AuCl_4_]·2H_2_O and Na[AuCl_3_(N_3_)]·2H_2_O, respectively, indicating that the formal zig‐zag chains Au–Au are slightly more stretched in the tetrachloride. However, both *d*(Au–Au) are considerably larger than the sum of the van der Waals radii (3.32 Å) [28], therefore ruling out any significant Au···Au interactions.

The coordination spheres of the chloride/azide ions rather mimic uncharged complexes than inorganic ionic compounds. In the complex anions [AuCl_4_]^–^/[AuCl_3_(N_3_)]^–^, one Cl connects only to gold as its nearest neighbor, the Cl/N_3_ (through the *inner* N species) as well as the two remaining Cl connect to Au and Na. By this, the coordination spheres of the ligands are significantly different compared to NaCl and NaN_3_, in which the anions are located in a Na octahedron. Considering size and shape of the coordination spheres of the Cl ligands of the [AuCl_4_]^–^ unit (Figure 1 center, solid atoms), only a single coordination site shows the sterically preferred and sufficiently large environment needed to accommodate the exchange Cl ↔ N_3_ (Figure 1 center, shaded atoms). Altogether, the linear N_3_ unit compared to the rather spherical Cl in the strictly planar complex anion [AuCl_4–_ *_x_* (N_3_)*_x_*]^–^ forces the lattice parameter *a* to increase by about 0.88 Å, whereas *c* (in plane) and *b* (perpendicular to plane) shrink slightly by 0.12 Å and 0.16 Å, respectively; however, structural changes due to the corresponding shifts of the atoms remain small.

The sevenfold coordination of Na by 4 O atoms (from crystal water) and 3 Cl / 2 Cl +1 N resembles a trigonal prism capped by Cl on the square side built up only by O. These units share common O–O edges to form 1D ···*AB*··· stacks along [010]. The resulting Na–Na zig‐zag chains in Na[AuCl_4_]·2H_2_O and Na[AuCl_3_(N_3_)]·2H_2_O with *d*(Na–Na) = 3.7449(13) Å / 3.7886(26) Å and  ∠(Na–Na–Na) = 141.17(1)° / 131.40(1)° are more pronounced than the Au–Au chains; the angles between the Na–Na and Au–Au chains are 87.8° / 88.7° along [100] and 64.4° / 66.9° along [010], respectively.

**FIGURE 2:**
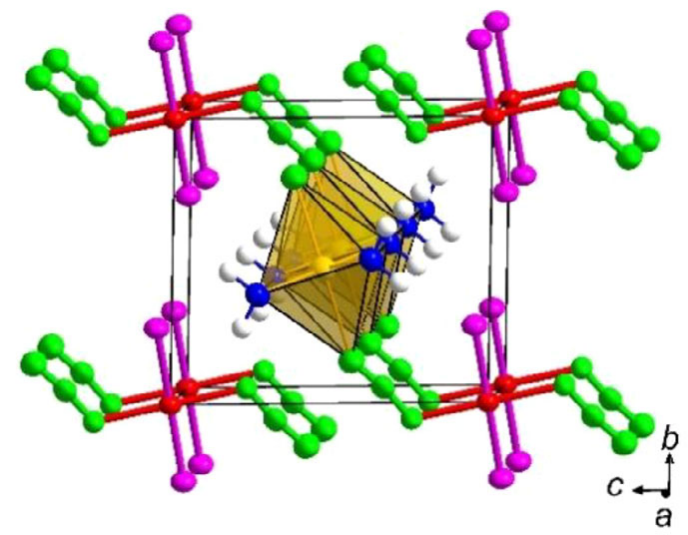
The crystal structure of Na[AuCl_2_(N_3_)_2_]·2H_2_O; Au red, Na yellow, Cl pink, N green, O blue, H white.

**Na[AuCl_2_(N_3_)_2_]·2H_2_O** (Figure 2) crystallizes in the triclinic space group *P* $\bar{1}$ (No. 2) with *Z* = 1 without any hint for a phase width. Depending on the Cl : N_3_ ratio in the starting materials, a deviation from 1 : 1 leads to mixtures of either Na[AuCl_3_(N_3_)]·2H_2_O and Na[AuCl_2_(N_3_)_2_]·2H_2_O or Na[AuCl_2_(N_3_)_2_]·2H_2_O and Na[AuCl(N_3_)_3_]·2H_2_O; samples containing all three or any other phases simultaneously are not observed.

The crystal structure of Na[AuCl_2_(N_3_)_2_]·2H_2_O was established from a twinned specimen, for more details, see Figure S3. As in Na[AuCl_3_(N_3_)]·2H_2_O, the predominant structural features in Na[AuCl_2_(N_3_)_2_]·2H_2_O are strictly planar complex anions [AuCl_2_(N_3_)_2_]^–^ with the gold atom located within a (slightly distorted) tetragonal planar coordination sphere built up by Cl and the *inner* N species and the N_3_ units located in *trans* position. Cl only connects to Au, whereas N_3_ connects to Au and Na through opposite N species.

The anions [AuCl_2_(N_3_)_2_]^–^ are stacked ···*AA*···, but tilted by 66.8° along [001] with both *d*(Au–Au) = *d*(Na–Na) = 3.6821(12) Å. Na is octahedrally coordinated by 4 O atoms (crystal water) forming the equatorial (quadrangle) square base and 2 *outer* N species from the azide ions forming the tips. These octahedra share common O–O edges to form stacks ···*AA*··· along [001].

**FIGURE 3:**
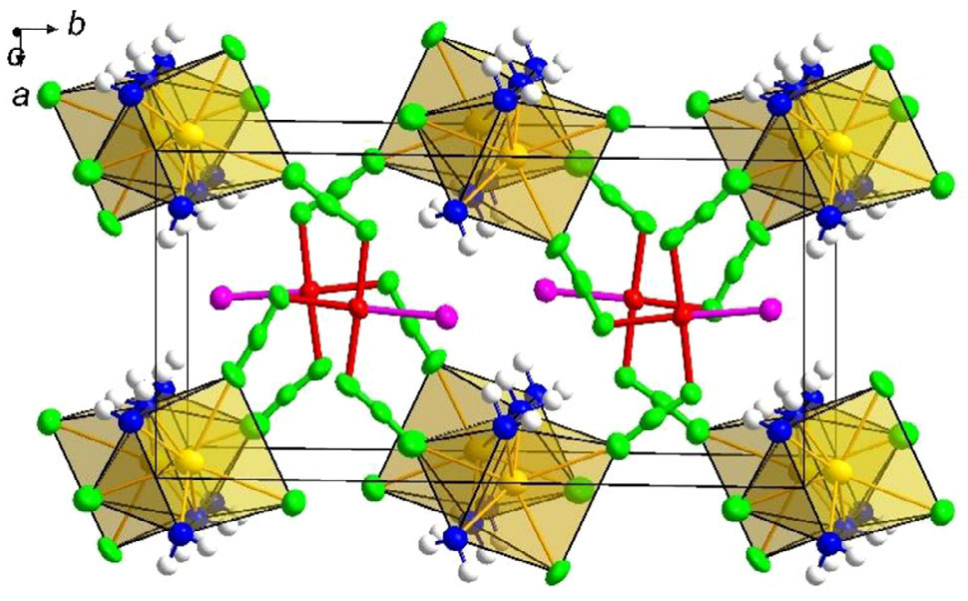
The crystal structure of Na[AuCl(N_3_)_3_]·2H_2_O; Au red, Na yellow, Cl pink, N green, O blue, H white.

**Na[AuCl(N_3_)_3_]·2H_2_O** (Figure 3) crystallizes in the orthorhombic space group *Pbcm* (No. 57) with *Z* = 4 without any hint for a phase width. Depending on the Cl : N_3_ ratio in the starting materials, a deviation from 1 : 3 leads to mixtures of either Na[AuCl_2_(N_3_)_2_]·2H_2_O and Na[AuCl(N_3_)_3_]·2H_2_O or Na[AuCl(N_3_)_3_]·2H_2_O and Na[Au(N_3_)_4_]·2H_2_O; samples containing all three or any other phases simultaneously are not observed.

The predominant structural features in Na[AuCl(N_3_)_3_]·2H_2_O are the complex anions [AuCl(N_3_)_3_]^–^. The gold center is located within a (slightly distorted) tetragonal planar coordination sphere built up by Cl and the *inner* N species of the azide ions, which are also located on this plane. Neither intermixing Cl/N nor disorder is observed. While Cl connects only to one Au, each of the three N_3_ anions connects to one Au and one Na through opposite N species. These complex anions are stacked ···*AB*··· along [001] with *d*(Au–Au) = 3.7542(7) Å and  ∠(Au–Au–Au) = 126.58(1)°, indicating formal zig‐zag chains Au–Au, but no significant Au···Au interactions.

As in Na[AuCl_3_(N_3_)]·2H_2_O, the sevenfold coordination of Na by 4 O atoms (from crystal water) and 3 N resembles a trigonal prism capped by N on the square side built up only by O. These units share common O–O edges to form 1D ···*AB*··· stacks along [001]. The resulting Na–Na zig‐zag chains with *d*(Na–Na) = 3.624(3) Å and  ∠(Na–Na–Na) = 135.49(1)° are more pronounced than the Au–Au chains; the angles between the Na–Na and Au–Au chains are ±17.2°.

**FIGURE 4:**
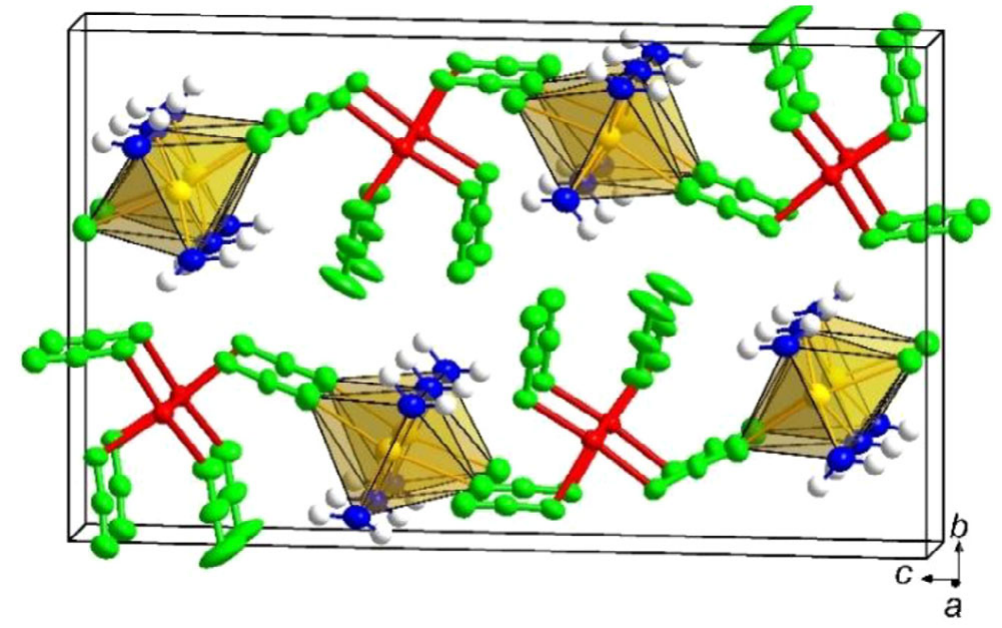
The crystal structure of Na[Au(N_3_)_4_]·2H_2_O; Au red, Na yellow, N green, O blue, H white.

**Na[Au(N_3_)_4_]·2H_2_O** (Figure 4) crystallizes in the monoclinic space group *P*2_1_/*c* (# 14) with *Z* = 4 without any hint for a phase width. An insufficient amount of N_3_^–^ in the starting materials leads to mixtures of Na[Au(N_3_)_4_]·2H_2_O and Na[AuCl(N_3_)_3_]·2H_2_O.

The predominant structural feature in Na[Au(N_3_)_4_]·2H_2_O are the complex anions [Au(N_3_)_4_]^–^. The gold atom is located within a (slightly distorted) tetragonal planar coordination sphere built up by the *inner* N species of the azide ions. Only the azide ions, which form through their *outer* N species the apices of the (NaO_4/2_N_2_) octahedra, are also located on this plane, whereas the other azide groups connect only to Au, and their dihedral angles to the AuN_4_ plane vary between 33° and 42°, indicating a significant torsion. These complex anions are stacked ···*AA*··· but tilted by 65.1° along [100] with *d*(Au–Au) = *d*(Na–Na) = 3.5843(6) Å. Na is octahedrally coordinated by 4 O atoms (crystal water) forming the equatorial square base and 2 *outer* N species from the azide ions forming the tips. These octahedra share common O–O edges to form stacks ···*AA*··· along [100].

The volume difference per formula unit of Na[AuCl_4–_ *_x_* (N_3_)*_x_*]·2H_2_O (*x* = 1, 2, 3, 4) for exchanging a chloride for an azide species corresponds to an increase of roughly 14 Å^3^ per exchanged ligand, a value similar to the difference of the molar volumes between NaCl and NaN_3_ (13.5 Å^3^) [9, 29].

### 2.2 Differential Scanning Calorimetry on Na[Au(N_3_)_4_]·2H_2_O

**FIGURE 5:**
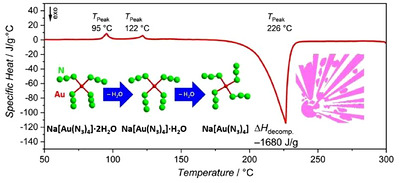
Dehydration and decomposition of Na[Au(N_3_)_4_]·2H_2_O.

The heating curve (heat capacity vs. temperature) of Na[Au(N_3_)_4_]·2H_2_O (Figure 5) shows two small endothermic effects (onset temperatures 92°C and 118°C), which correlate with dehydration in two steps to Na[Au(N_3_)_4_]·H_2_O and Na[Au(N_3_)_4_] and one strong exothermic signal (onset 212°C, peak temperature 226°C), which describes the explosive decomposition of Na[Au(N_3_)_4_].

An energy of approx. –1680 J/g (–652 kJ/mol) is released. In comparison, the value measured for AgN_3_ is –1943 J/g (–291 kJ/mol). Compared to the literature value of –279 kJ/mol [30], this is within the usual margin of error of 5% to 10% for this method. After decomposition, a pink precipitate remains in the container, indicating the presence of gold nanoparticles [31].

### 2.3 Thermal Investigations on Na[AuCl_4–_ *_x_* (N_3_)*_x_*]·2H_2_O (*x* = 0–3)

According to DTA/TG investigations in natural air, dehydration and decomposition occur almost in parallel in the mixed chlorido/azidoaurates Na[AuCl_3_N_3_]·2H_2_O, Na[AuCl_2_(N_3_)_2_]·2H_2_O, and Na[AuCl(N_3_)_3_]·2H_2_O. In contrast to Na[Au(N_3_)_4_]·2H_2_O (Figure 5), no two‐stage water release is observed. The dehydration temperature decreases with increasing azide content of the phases (Na[AuCl_4_]·2H_2_O: 119°C, Na[AuCl_3_N_3_]·2H_2_O: 99°C, Na[AuCl_2_(N_3_)_2_]·2H_2_O: 85°C and Na[AuCl(N_3_)_3_]·2H_2_O: 78°C).

Significant but less pronounced exothermic effects (*T*_peak_ ≈ 150–160°C) at a much lower temperature compared to Na[Au(N_3_)_4_] (*T*_peak_ = 226°C) are also observed, which can be correlated with the formation of Na[AuCl_4_] (detected as rehydrated Na[AuCl_4_]·2H_2_O, as the samples were exposed to natural air after the DTA analysis), NaCl, and Au, as shown in the X‐ray powder diagrams of the products according to, for example

$$6\;\mathrm{Na}\left[\mathrm{AuC}l_{3}N_{3}\right]\;\to \;4\;\mathrm{Na}\left[\mathrm{AuC}l_{4}\right]+2\;\mathrm{NaCl}+2\;\mathrm{Au}+9\;N_{2}$$

High‐temperature diffraction measurements on powders were performed in natural air in the range up to 100°C and confirmed the DTA/TG investigations (Figure S6 a–d) concerning dehydration. No changes were observed in the X‐ray powder diffraction diagrams for Na[AuCl_4_]·2H_2_O and Na[AuCl_3_N_3_]·2H_2_O; for Na[AuCl_2_(N_3_)_2_]·2H_2_O (∼ 90°C), Na[AuCl(N_3_)_3_]·2H_2_O (∼ 70°C), and Na[Au(N_3_)_4_]·2H_2_O (62°C, 87°C; Figure S6e), dehydration sets in earlier and earlier as the azide content increases, but only in the latter phase, a two‐step process is evident. No attempts were undertaken to further identify these new dehydrated mixed chlorido/azidoaurate phases. After cooling, immediate rehydration to the starting materials follows; increasingly strong Au and NaCl peaks indicate partial decomposition.

In conclusion, it should be noted that the thermal decomposition behavior of the multianionic chlorido/azidoaurates differs significantly from Na[Au(N_3_)_4_]·2H_2_O proceeding to the preferential formation of Na[AuCl_4_] without an explosion.

### 2.4 Crystal Structures of the Sodium Tetraazidoaurate Monohydrate and Sodium Tetraazidoaurate

The crystal structures of **Na[Au(N_3_)_4_]·H_2_O** and **Na[Au(N_3_)_4_]** were solved and refined from X‐ray powder diffraction data.

**FIGURE 6:**
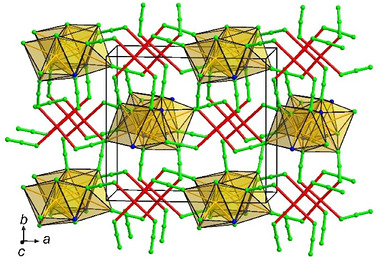
The crystal structure of Na[Au(N_3_)_4_]·H_2_O; Au red, Na yellow, N green, O blue.

**Na[Au(N_3_)_4_]·H_2_O** (Figure 6) crystallizes in the orthorhombic space group *P*2_1_2_1_2_1_ (# 19) with *Z* = 4 without any hint for a phase width (Figure S4, S5).

The predominant structural features in Na[Au(N_3_)_4_]·H_2_O are the complex anions [Au(N_3_)_4_]^–^. The gold center is located within a (slightly distorted) tetragonal near planar coordination sphere built up by the *inner* N species of the azide ions. All azide ions are connected through their outer N species to one or two Na species each and are located outside the AuN_4_ plane with dihedral angles of 4.0°, 4.6, 20.0°, and 36.7°, respectively. These complex anions are stacked ···*AB*··· along [001] with *d*(Au–Au) = 3.746(4) Å and  ∠(Au–Au–Au) = 128.9(1)°, very similar to Na[AuCl(N_3_)_3_]·2H_2_O and indicating formal zig‐zag chains Au–Au, but no significant Au···Au interactions.

The Na species are eightfold coordinated by 6 N and 2 O atoms (from crystal water), resembling a highly distorted tetragonal antiprism. These units share common N_2_O faces to form 1D ···*AB*··· columns along [001]. The resulting Na–Na zig‐zag chains with *d*(Na–Na) = 3.72(3) Å and  ∠(Na–Na–Na) = 130.8(8)° are slightly less pronounced than the Au–Au chains; the Na–Na and the Au–Au chains run nearly parallel.

Refinement of the H positions remained unsuccessful from the X‐ray powder diffraction data. Potential H sites are discussed in Figure S7.

**FIGURE 7:**
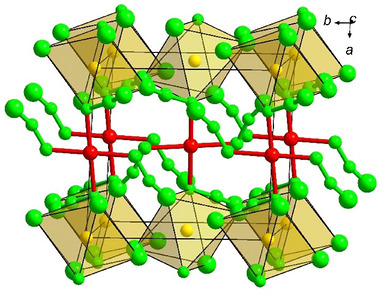
The crystal structure of Na[Au(N_3_)_4_]; Au red, Na yellow, N green.

**Na[Au(N_3_)_4_]** (Figure 7) crystallizes in the monoclinic space group *P*2_1_/*c* (# 14) with *Z* = 2 without any hint for a phase width.

As in all other tetraazidoaurates, the predominant structural features in Na[Au(N_3_)_4_] are the complex anions [Au(N_3_)_4_]^–^. The gold center is located within a (slightly distorted) tetragonal near planar coordination sphere built up by the *inner* N species of the two symmetry‐independent azide ions. One of these azide ions is further connected through the *inner* and *outer* N species to two Na cations, the other azide ion connects through the *outer* N species to one Na species. The azide ions are located slightly outside the AuN_4_ plane with dihedral angles of 11.5° and 18.2°, respectively. The Na species are octahedrally coordinated by N.

In contrast to all other phases presented here, no 1D entities are recognizable, but both the complex anions [Au(N_3_)_4_]^–^ and the isolated octahedra (NaN_6_) are arranged in layers in (011) plane with an angle between the Au layer and the AuN_4_ plane of 73.3°.

### 2.5 Structural Considerations

**FIGURE 8:**
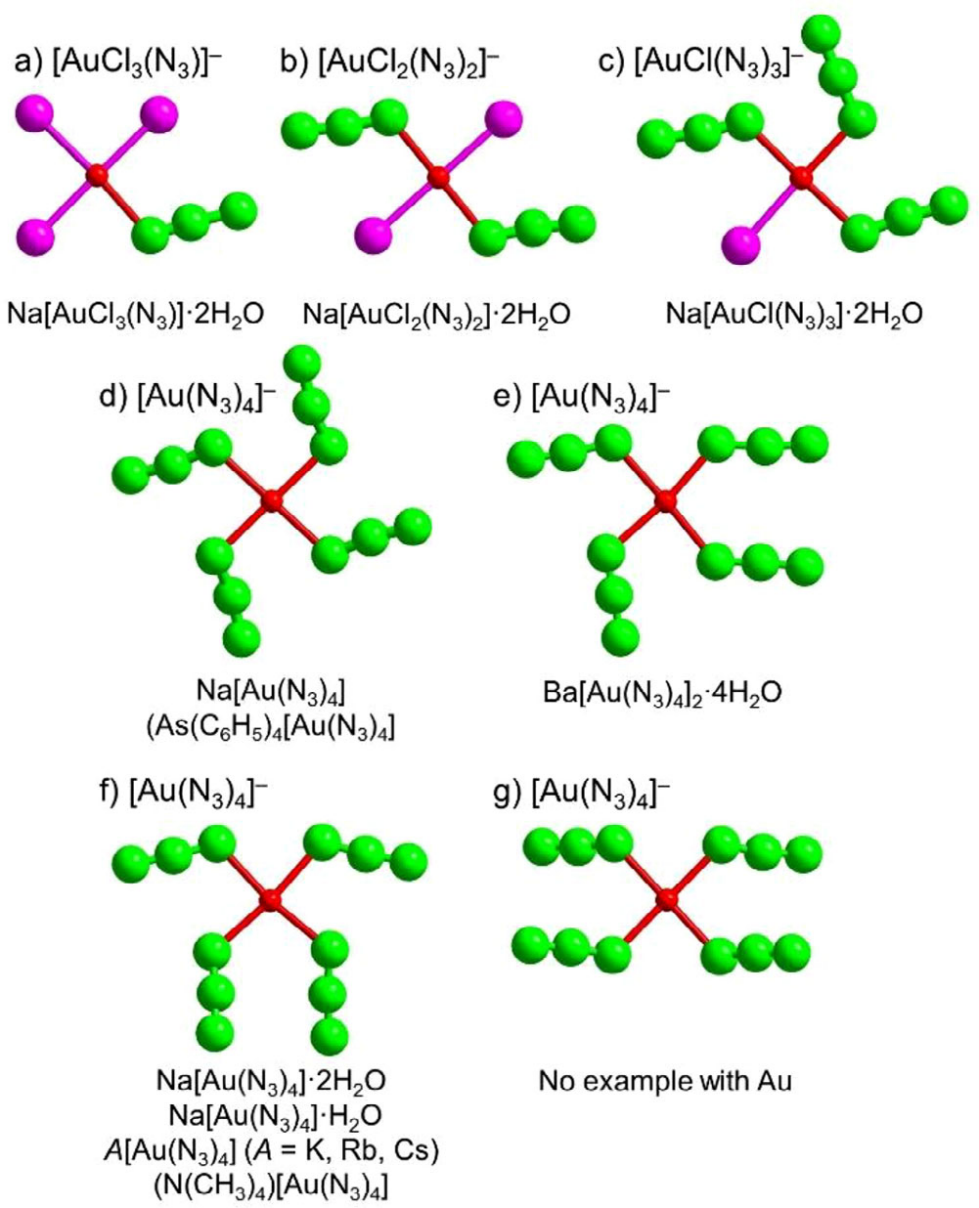
Overview of the different types and conformations of complex anions [AuCl_4–_ *_x_* (N_3_)*_x_*]^–^ (*x* = 1, 2, 3, 4) in azidoaurates(III) [20, 21, 22, 24]. Similar square‐planar anions have also been observed in azidopalladates(II) (d) [23, 32], e) [23], g) [23]) and azidoplatinates(II) (d) [32]), respectively.

Azidoaurate anions [Au(N_3_)_4_]^–^ as well as mixed anions [AuCl_4–_ *_x_* (N_3_)*_x_*]^–^ (Figure 8) share the same building principles. Predominant structural feature is the (slightly distorted) tetragonal planar coordination of the central atom by the *inner* N species of the azide ions, but a wide variety of conformations besides the ubitiquous windmill pattern exists.

**FIGURE 9:**
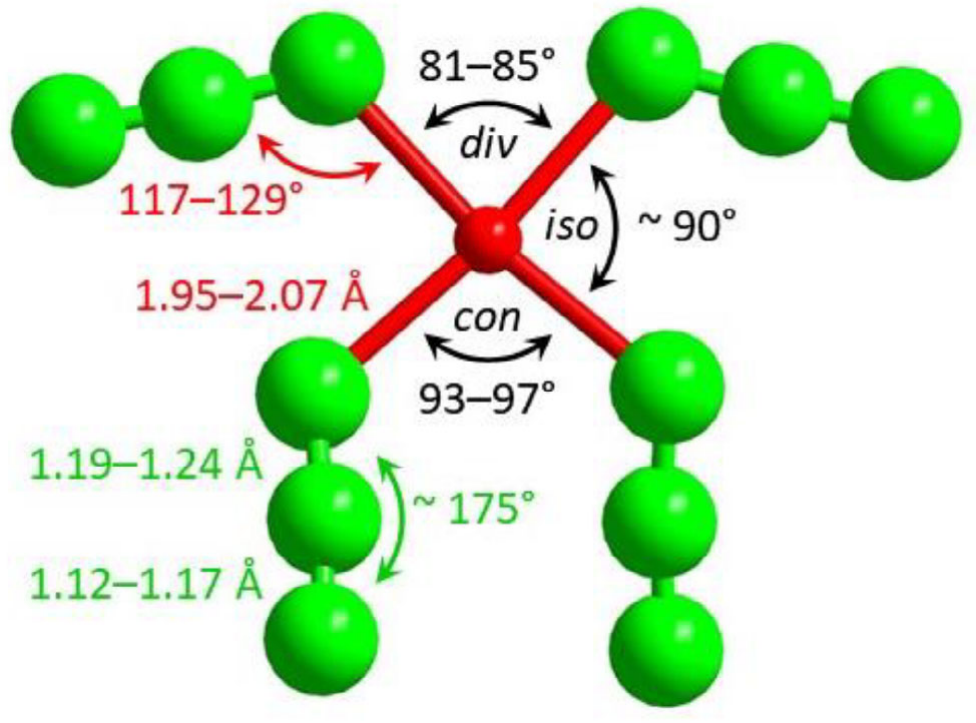
Distance and angle trends in the azidoaurate anion [Au(N_3_)_4_]^–^.

For all complex anions [AuCl_4–_ *_x_* (N_3_)*_x_*]^–^, distances and angles tend to fall in discrete regions depending on the conformation of the anion, but independent of the nature of the cationic partial structure (Figure 9). According to single crystal data, the distances *d*(Au–N) are in the range between 1.95 and 2.07 Å, *d*(Au–Cl) = 2.27–2.31 Å (not shown), the *inner d*(N–N) vary between 1.19 and 1.24 Å, and the *outer d*(N–N) between 1.12 and 1.17 Å as expected from literature data. In general, the anisotropic displacement parameters of Au and the *inner* and *central* N of the azide ion are well defined and rather spherical, whereas the displacement parameters of the *outer* N are rather elongated perpendicular to the N─N bond when not contacting any further cation, as evidenced in, for example Figures 1, 4.

The angles Au–N–N are in the range 117–129°, the angles N–N–N around 175°. For Na[Au(N_3_)_4_]·H_2_O and Na[Au(N_3_)_4_], the values differ slightly, presumably due to the refinement from powder X‐ray data.

In simplified form, the mutual orientation of neighboring azide groups on the central atom can be described as equal (*iso*), divergent (*div*), or convergent (*con*). The N–Au–N angles of neighboring groups can be arranged accordingly; the trends remain the same in each case, but the order of magnitude can change. *Iso* angles are in the range of 90°, *div* angles tend to be smaller at 81–85°, while *con* angles are larger at 93–97°.

**FIGURE 10:**
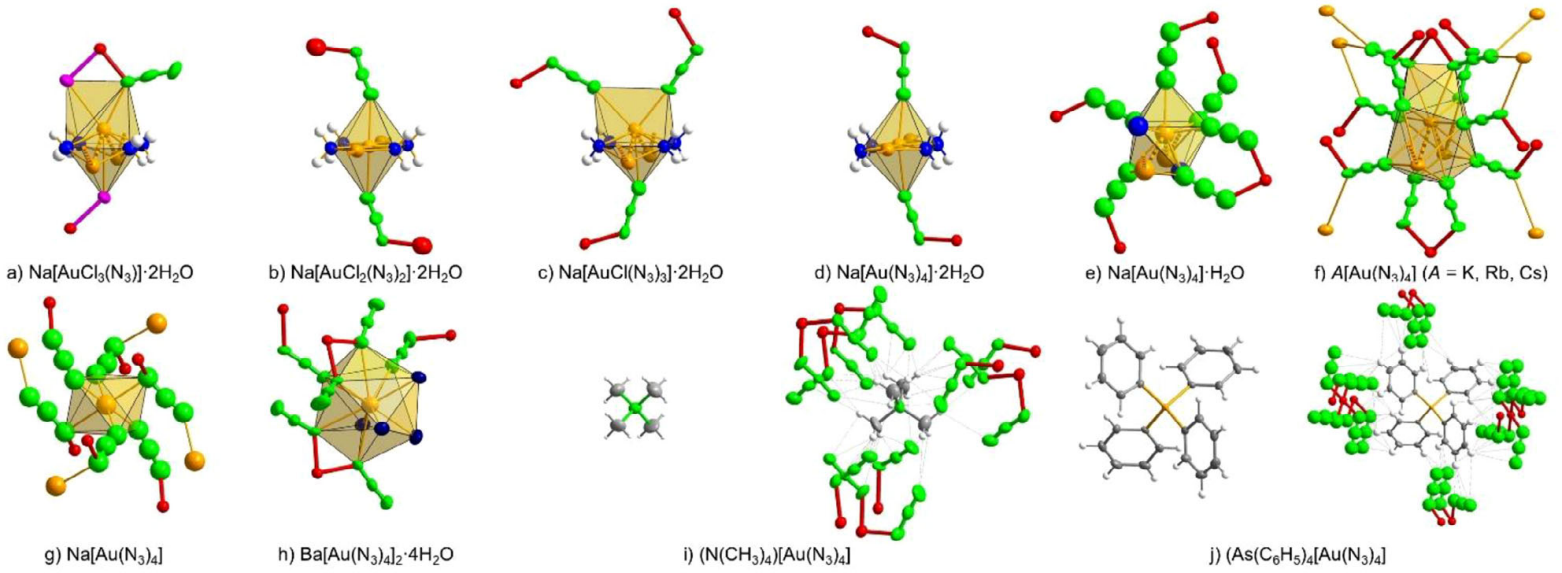
Overview of the different cationic partial structures in azidoaurates(III) [20, 21, 22, 24].

The cationic partial structures differ significantly depending on the nature of the cations and on the presence of crystal water in the structures (Figure 10). For all dihydrates containing sodium Na[AuCl_4–_ *_x_* (N_3_)*_x_*]·2H_2_O (*x* = 1, 2, 3, 4) (Figure 10a–d), 1D arrangements result with Na in either a sevenfold (Figure 10a, c) or sixfold (Figure 10b, d) coordination by N, O, and—in the case of Na[AuCl_3_(N_3_)]·2H_2_O—by Cl. All these coordination polyhedra share common edges formed by oxygen. In Na[Au(N_3_)_4_]·H_2_O (Figure 10e), Na is eightfold coordinated by 6 N (from five different [Au(N_3_)_4_] units) and 2 O species; by sharing two N_2_O faces, these polyhedra are also stacked to 1D chains. The coordination of the alkali cation *A* (Figure 10f) in *A*[Au(N_3_)_4_] (*A* = K, Rb, Cs) [20] is built up by 10 N species belonging to 8 different [Au(N_3_)_4_] units; neighboring polyhedra share common tetragonal phases to form chains. In both Na[Au(N_3_)_4_] and Ba[Au(N_3_)_4_]_2_·4H_2_O [24], the coordination polyhedra around the cations are isolated and built‐up by 6 nitrogen (Na[Au(N_3_)_4_], Figure 10g) or 6 nitrogen and 4 oxygen atoms (Ba[Au(N_3_)_4_]_2_·4H_2_O, Figure 10h), respectively. The coordination spheres of the complex cations [N(CH_3_)_4_] (Figure 10i left) and [As(C_6_H_5_)_4_] (Figure 10j left) in [N(CH_3_)_4_][Au(N_3_)_4_] [22] and [As(C_6_H_5_)_4_][Au(N_3_)_4_] [21] are difficult to deduce; neither analysis of *d*(N–H) nor application of methods like the Brunner‐Schwarzenbach formalism [33] leads to reliable results, so it remains to state that [N(CH_3_)_4_] is coordinated by nine and [As(C_6_H_5_)_4_] by ten different [Au(N_3_)_4_] units.

Although all compounds presented in this chapter may be formally considered as pseudobinary phases *AB* or *AB*_2_ (in the case of (Ba(H_2_O)_4_)[Au(N_3_)_4_]_2_), the centers of gravity of the constituents do not necessarily mimic a known simple structure type.

### 2.6 Vibrational Spectra of the Mixed Chlorido‐/Azidoaurates Na[AuCl_4–_ *_x_* (N_3_)*_x_*]·2H_2_O (*x* = 1, 2, 3)

**FIGURE 11:**
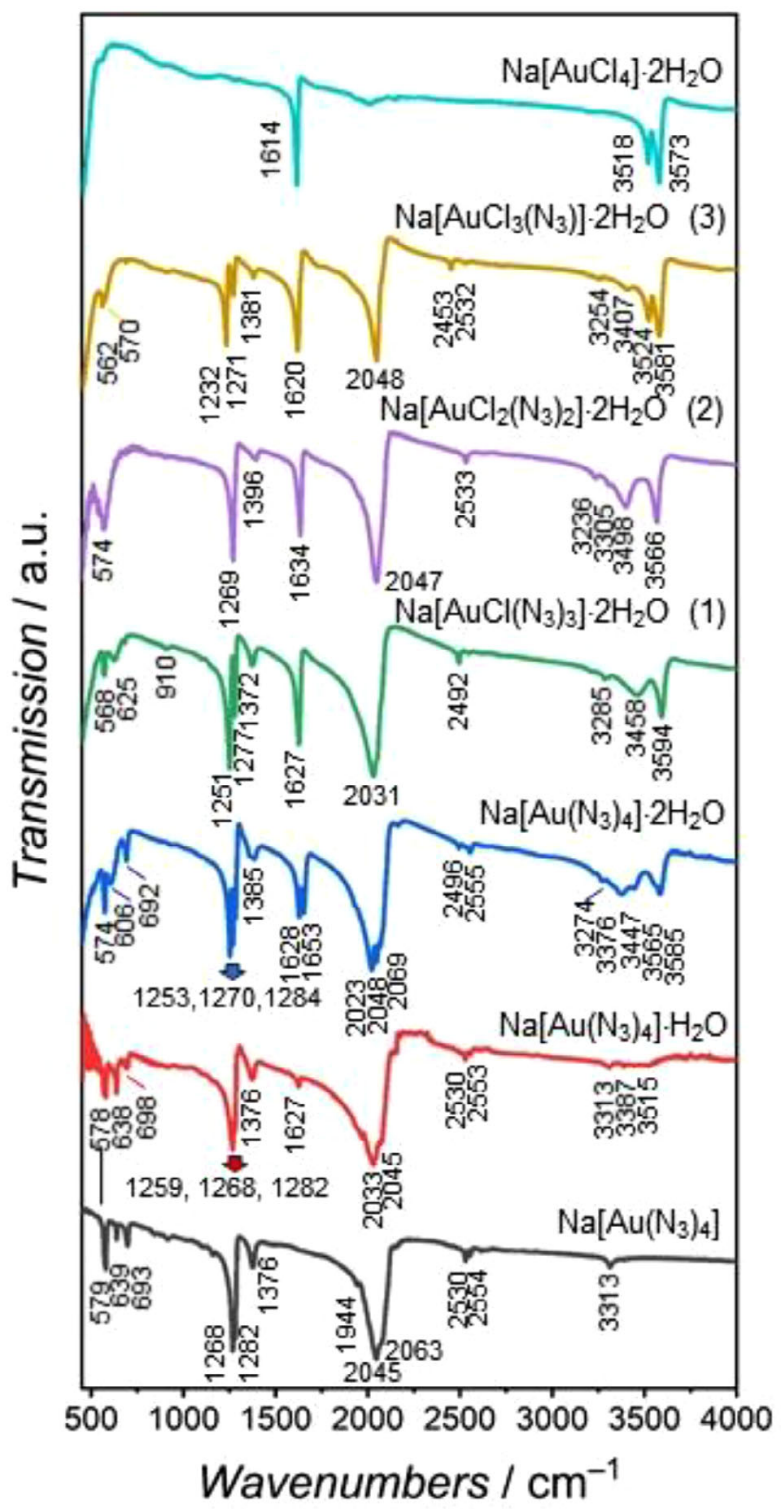
Infrared spectra of Na[AuCl_4–_ *_x_* (N_3_)*_x_*]·2H_2_O (*x* = 0, 1, 2, 3, 4), Na[Au(N_3_)_4_]·H_2_O, and Na[Au(N_3_)_4_] in the region 450–4000 cm^–^^1^. Transmission (IR‐ATR) in arbitrary units. For experimental conditions, see text.

The vibrationally relevant units in the mixed compounds Na[AuCl_3_(N_3_)]·2H_2_O (1), Na[AuCl_2_(N_3_)_2_]·2H_2_O (2) and Na[AuCl(N_3_)_3_]·2H_2_O (3) are square‐planar complex anions [AuCl_4–_ *_x_* (N_3_)*_x_*]^–^. Infrared and Raman spectra are shown in Figures 11 and S8–S10 together with reference spectra of Na[AuCl_4_]·2H_2_O and Na[Au(N_3_)_4_]·2H_2_O.

In the Raman spectra (Figure S8), three characteristic regions are observed:

(i) Au–Cl stretching (< 350 cm^–^^1^),
(ii) Au–N stretching and deformation (ca. 200–1300 cm^–^^1^), and
(iii) N_3_ group modes (650–2100 cm^–^^1^).

The ν(Au–Cl) band of Na[AuCl_4_]·2H_2_O at 346 cm^–^^1^ decreases in intensity and shifts to lower energy upon progressive azide substitution, appearing at 327 cm^–^^1^ in (1). These trends directly reflect the decreasing number of Au–Cl bonds and symmetry changes from *D*_4_ *_h_* to lower point symmetries in the mixed anions.

The ν (Au–N) stretching region behaves analogously: compound (3) shows a characteristic doublet, while (1) and (2) contain single Au–N features, consistent with the different ligand arrangements.

The azide vibrations show strong similarities across the series. Weak Raman signals at ∼685 cm^–^^1^ originate from out‐of‐plane bending of the azide unit, while the symmetric and antisymmetric azide stretching bands appear at ∼1230–1280 and ∼2030–2070 cm^–^^1^, respectively. Their positions demonstrate that the azide units behave essentially as “quasi‐free” ligands; changes between (1)—(3) are small and originate mainly from local geometry.

The IR spectra (Figures 11b–d and S8) of (1)—(3) confirm these assignments and are dominated by the strong azide absorptions, while Au–Cl stretches are absent due to experimental cut‐off (< 450 cm^–^^1^). Peaks from crystal water (νOH and δOH at ∼3500 and ∼1600 cm^–^^1^) appear strongly because of intense hydrogen bonding.

Overall, the vibrational characteristics of the mixed compounds follow the structural evolution: progressive azide incorporation weakens ν(Au–Cl), strengthens Au–N contributions, and preserves the square‐planar nature of the anions.

It is interesting to note that in IR spectra of mixtures [AuCl_4–_ *_x_* (N_3_)*_x_*]·2H_2_O (0 ≤ *x* ≤ 4) always only modes of two neighboring phases may be discerned (Figure S11).

### 2.7 Vibrational Spectra of Na[Au(N_3_)_4_]·2H_2_O

Na[Au(N_3_)_4_]·2H_2_O crystallizes with one crystallographically distinct azidoaurate unit [Au(N_3_)_4_]^–^ per asymmetric cell. Symmetry reduction (from ideal *C*_4_ *_h_* to *C*_1_ local symmetry) and the presence of *Z* = 8 formula units lead to extensive mode splitting, clearly reflected in the spectra (Figures 11 and S9).

The ν(Au–N) stretches appear at 414 and 397 cm^–^^1^ (symmetric and antisymmetric), observable only in Raman due to experimental IR limitations. Azide stretching band groups occur at 1250–1285 and 2020–2080 cm^–^^1^ and appear as triplets in both Raman and IR because of factor group splitting. Additional bending modes arise around 690–600 cm^–^^1^.

Strong hydrate water absorptions (δ at 1628/1653 cm^–^^1^ and ν at 3274–3585 cm^–^^1^) correlate with the presence of two independent water molecules. Their intensity pattern reflects the 2×8 interacting water molecules in the cell.

### 2.8 Spectra of Na[Au(N_3_)_4_]·*n*H_2_O (*n* = 2, 1, 0)

Sequential dehydration (*n* = 2 → 1 → 0) yields markedly simplified IR spectra (Figures 11 and S10). The disappearance of hydrate‐water bands confirms water release; water uptake proceeds directly from *n* = 0 → 2 (Figure S12). Peak patterns of the azide stretches remain essentially unchanged, demonstrating that dehydration does not alter the fundamental geometry of the [Au(N_3_)_4_]^–^ anion.

The remaining band multiplicity decreases stepwise, consistent with reduced factor group splitting (*Z* = 4 and 2 for *n* = 1 and 0, respectively). The vibrational signatures therefore reflect primarily symmetry effects, not structural transformations. For more information, please refer to SI.

### 2.9 DFT Calculation and Summary

**FIGURE 12:**
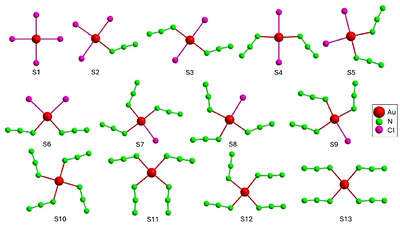
Optimized geometry structures of complexes [AuCl_4–_ *_x_* (N_3_)*_x_*] (*x* = 0, 1, 2, 3, 4); Au red, Cl pink, N green.

Structural, spectroscopic, and theoretical investigations of the series Na[AuCl_4–_ *_x_* (N_3_)*_x_*]·2H_2_O (*x* = 0, 1, 2, 3, 4) (Figure 12) provide a robust basis for understanding the electronic consequences of the stepwise chloride–azide substitution in square‐planar Au(III) complexes. DFT calculations (B3LYP/LANL2DZ) on isolated anions reproduce the experimentally observed Au–N, Au–Cl, and N–N bond metrics with high accuracy (Table S9), consistent with earlier computational studies on heavy‐metal azides and Au(III) complexes [34, 35, 36, 37, 38, 39]. Both theory and XRD consistently show that Au–N bonds (2.079–2.114 Å, force constants 1.754–2.009 Ncm^–^^1^) are significantly shorter and stronger than the corresponding Au–Cl bonds (2.430–2.464 Å, force constants 1.456–1.739 Ncm^–^^1^). This hierarchy is likewise reflected in published structural data for alkali azidoaurates [20, 24]. The intense yellow–orange colors of the crystals reflect the high covalency of the Au–N bond and the presence of ligand‐to‐metal charge‐transfer transitions, in agreement with general observations on azido complexes [22, 27, 40].

The internal bonding pattern of the azide ligand is remarkably uniform across the entire series. All azide‐containing complexes exhibit two distinct N–N bonds—one short/strong and one long/weaker—matching the established asymmetric resonance‐stabilized bonding model of N_3_^−^ [4, 41]. This rigidity is likewise reflected in the vibrational spectra, where the symmetric (1200–1300 cm^–^^1^) and antisymmetric (2000–2100 cm^–^^1^) N═N═N stretches appear consistently throughout the series, in agreement with previously reported azidoaurate spectra [14, 20, 24, 27]. The modest band splittings in Na[Au(N_3_)_4_]·2H_2_O arise from symmetry reduction and factor‐group splitting in the solid state rather than intrinsic perturbation of the azide unit, consistent with factor‐group analyses in metal–azide crystals [24, 42].

DFT also reveals a slight but systematic weakening of the Au–N bond as more azide ligands are introduced. For example, the calculated force constant decreases from 1.954 Ncm^–^^1^ in the monoazide Na[AuCl_3_(N_3_)] (S2) to 1.877 Ncm^–^^1^ in the tetraazido species Na[Au(N_3_)_4_] (S10). This trend reflects increasing electronic saturation at the Au(III) center, a phenomenon also noted earlier in computational and spectroscopic studies of polynitrogen‐coordinated Au(III) complexes [43]. Raman spectroscopy corroborates this behavior: the ν(Au–Cl) band shifts from 348 to 327 cm^–^^1^ upon progressive chloride replacement by azide (Figure S8b **),** consistent with spectroscopic trends reported for mixed chlorido–azido Au(III) complexes in the literature [44].

The intrinsic electronic sensitivity of Au(III)–azide complexes also manifests in their redox behavior. As demonstrated elsewhere, the tetraazidoaurate(III) anion undergoes photoreduction to [Au(N_3_)_2_]^–^ in nonaqueous solvents such as THF [27, 45]. The energetic nature of these compounds is further supported by DSC measurements on Na[Au(N_3_)_4_]·2H_2_O, where dehydration (endothermic peaks at 95°C and 122°C) is followed by a strongly exothermic decomposition at 226°C (≈ –1680 Jg^–^^1^). This behavior is in line with the known thermal instability of heavy‐metal azides [46, 47]. The hydrated forms of the salts thus provide crucial kinetic stabilization, consistent with hydration‐stabilized azidoaurates and azidopalladates reported previously [23, 24], whereas anhydrous materials may display strong photolability, in agreement with known photoreduction pathways of Au(III) azides mentioned above. In situ IR monitoring confirmed the protective role of lattice water during dehydration and transformation into the highly sensitive anhydrous phase.

In conclusion, the vibrational and structural data for Na[AuCl_4–_ *_x_* (N_3_)*_x_*]·2H_2_O (*x* = 0–3) and Na[Au(N_3_)_4_]·*n*H_2_O (*n* = 0–2) show excellent agreement with DFT calculations and with reported structures of alkali and alkaline‐earth azidoaurates such as *A*[Au(N_3_)_4_]·*n*H_2_O (*A* = K, Rb, Cs, [As(phen)_4_], *n* = 0–2) and Ba[Au(N_3_)_4_]_2_]·4H_2_O [20, 21, 24]. Substitution‐dependent shifts in the Au–N and N–N stretching regions can be rationalized through ligand‐field effects and local symmetry variations.

The correlation between the ν_as_(N_3_) stretching frequencies and the electronic nature of the coordinating ligand *X* in complexes of the type *X–*Au(N_3_)*_n_* is not straightforward. While a simple inductive model predicts higher ν_as_(N_3_) for more electronegative ligands, numerous studies have shown that halides—particularly chloride—act as π‐donors and redistribute back‐donation at the Au(III) center, thereby disrupting this trend [4]. Because azide is itself a strong π‐acceptor, especially in multiply coordinated environments, it dominates the electronic demand, leading to deviations from simple electronegativity‐based expectations. Comparable π‐competition effects have been reported in Au(III), Pt(IV), and other *d* ^8^ azide/halide systems [44], and the present data follow this established pattern.

## 3 Experimental Section

### 3.1 Synthesis

Due to the highly explosive nature of the title compounds, all handlings were carried out under extreme safety precautions during experiments (protective shields, goggles, and gloves) using minute amounts of sample. It is essential to wear protective gear and to prevent shock, heat, and intense radiation on samples.

White solid NaN_3_ (Merck, 99.9%, recrystallized in water) and blackish AuCl_3_ (Thermo Fisher Scientific, 99.99%) were starting materials to obtain all phases **Na[AuCl_4–_ *_x_* (N_3_)*_x_*]·2H_2_O (*x* = 1, 2, 3, 4)**; in all experiments an excess of NaN_3_ had to be employed to obtain specimen containing only one single azidochloridoaurate phase.

Each of the starting materials was dissolved in water; upon combining, the color of the aqueous solution instantaneosly changed to a deep orange. After few days, large transparent orange‐red crystalline agglomerates in the form of brittle and often twinned elongated plates or orthogonal columns were obtained upon evaporation of the solvent. Most of the colorless by‐products, presumably unreacted NaCl, NaN_3_, and other phases, could be mechanically removed. Repeated recrystallization in first methanol and then water not only removed the remaining traces of impurities but also improved crystal quality.

The use of HAuCl_4_ (Sigma Aldrich, 99.9%) instead of AuCl_3_ gave similar results. Temperature treatment experiments were performed using either a heat plate or an ice bath in the range of 0°C–50°C without improving results.

Upon heating, removal of crystal water proceeds in one step in **Na[AuCl_4–_ *_x_* (N_3_)*_x_*]·2H_2_O (*x* = 0, 1, 2, 3)** at temperatures between 119°C and 78°C; the resulting phases were not further investigated. However, exhaustive experiments to remove crystal water, either by slow heating or by cautious evacuation, were performed exclusively with sodium tetraazidoaurate dihydrate Na[Au(N_3_)_4_]·2H_2_O. Whereas the water‐free phase **Na[Au(N_3_)_4_]** is obtained easily, for example, by heating to 80°C in a drying oven for at least 1 h, a complex low‐temperature process was developed to obtain near single‐phase specimen of **Na[Au(N_3_)_4_]·H_2_O**. Putting completely dehydrated water‐free samples outside the glovebox into air led to rehydration to Na[Au(N_3_)_4_]·2H_2_O within two minutes without evidence of intermediate formation of the monohydrate phase Na[Au(N_3_)_4_]·H_2_O.

Due to the highly explosive nature of the title compounds, the content of N and O in the bulk material could not be determined quantitatively by carrier gas hot‐extraction technique. In preliminary experiments with sample amounts below 1 mg, heavy explosions threatened to damage not only the crucibles used for the analysis but also the analyzer itself.

For details on the experimental procedures, see Supporting Information.

### 3.2 X‐ray Diffraction

Single crystals of the phases Na[AuCl_4–_ *_x_* (N_3_)*_x_*]·2H_2_O (*x* = 1, 2, 3, 4), which were suitably sized for X‐Ray single crystal diffraction, were glued to the tips of glass fibers. Intensity data collection was performed at room temperature on a Rigaku AFC7 automatic diffractometer equipped with a Saturn 724+ CCD detector with Mo*K*α radiation using a graphite‐monochromator and the φ oscillation scan technique. The crystal structures were solved using SHELXS‐2018 and refined using the full‐matrix least‐squares procedure with the SHELXL‐2018 program package, see Tables S1–S8 for details [48, 49]. It was not possible to localize and refine hydrogen positions without restraints. The highest peaks in the residual electron density maps are located close to Au and have no structural significance.

Temperature‐dependent powder X‐ray diffraction data up to the instrumental limit of 102°C were collected on a STADI P diffractometer (Stoe & Cie, Darmstadt, Germany) using Ge‐monochromatized Cu*K*α_1_ radiation and a Mythen detector (Dectris, Baden, Switzerland) in Debye‐Scherrer geometry in 0.5 mm borosilicate glass capillaries. Heating and cooling were realized using an N_2_ Cryostream system (Oxford Cryosystems, Oxford, UK), allowing a sample equilibration for at least 30 min before the individual measurements.

The crystal structures of Na[Au(N_3_)_4_]·H_2_O and Na[Au(N_3_)_4_] were solved from powder X‐ray diffraction data (Figures S4, S5) using JANA 2020 and FOX program packages [50, 51]; the evolution from Na[Au(N_3_)_4_]·2H_2_O to Na[Au(N_3_)_4_] was investigated by a high‐temperature powder diffraction measurement (Figure S6).

Deposition Numbers = CSD‐2486294 (for Na[AuCl_4_]·2H_2_O), CSD‐2486295 (for Na[AuCl_3_(N_3_)]·2H_2_O), CSD‐2486289 (for Na[AuCl_2_(N_3_)_2_]·2H_2_O), CSD‐2486285 (for Na[AuCl(N_3_)_3_]·2H_2_O), CSD‐2486284 (for Na[Au(N_3_)_4_]·2H_2_O), CSD‐2486383 (for Na[Au(N_3_)_4_]·H_2_O), and CSD‐2486567 (for Na[Au(N_3_)_4_]) contain the supplementary crystallographic data for this paper. These data are provided free of charge by the joint Cambridge Crystallographic Data Centre and Fachinformationszentrum Karlsruhe <url href = “http://www.ccdc.cam.ac.uk/structures”>Access Structures service</url>. Graphical representations of the structure were created in Diamond [52].

### 3.3 Differential Scanning Calorimetry

The thermal behavior of **Na[Au(N_3_)_4_]·2H_2_O** and AgN_3_ (for comparison) was characterized using a power‐compensated DSC (DSC 8500, from Perkin Elmer). Samples of the compounds were measured under the following conditions: sample mass: 1.0 mg, (AgN_3_ 2.5 mg) heating and cooling rate: 10 K/min, crucible: screwable high‐pressure stainless‐steel crucible. The measurements were carried out in a temperature range from 25 to 400°C in a flowing argon atmosphere (Ar 99.999% 100 mL/min with additional drying and oxygen post‐purification via a Big Oxygen Trap from Trigon Technologies).

The temperature and heat flow were calibrated with indium and zinc. The evaluation was carried out using the sapphire method.

### 3.4 Thermal Analysis

The thermal decomposition behavior of **Na[AuCl_4_]·2H_2_O**, **Na[AuCl_3_N_3_]·2H_2_O**, **Na[AuCl_2_(N_3_)_2_]·2H_2_O**, and **Na[AuCl(N_3_)_3_]·2H_2_O** was also investigated by DTA/TG using an STA 409 (NETZSCH). The individual samples were measured under the following conditions: atmosphere: static ambient air, temperature range: 25 to 200°C, heating and cooling rate: 5 K/min, sample mass: 4.1 to 6.7 mg, crucible: corundum DTA/TG crucible with perforated lid, thermocouple: type S (PtRh/Pt).

### 3.5 Vibrational Spectroscopy

FT‐Raman spectra were obtained using a Renishaw inVia Raman microscope equipped with a 532 nm excitation laser source. The Raman spectra of selected crystals were recorded at room temperature in the range of 4000 to 50 cm^−1^ using either a Horiba LabRam HR Evolution spectrometer equipped with a He‐Ne laser (633 nm) and a Synapse CCD detector or Bruker Senterra II micro‐Raman spectrometer equipped with a diode laser (λ = 532 nm).

Infrared spectra were recorded in Attenuated Total Reflectance (ATR) mode using either a Thermo Scientific iS50 FT‐IR spectrometer with a single reflection diamond ATR module or a PerkinElmer UATR‐Two FTIR spectrometer (spectral range 4000−450 cm^−1^).

### 3.6 DFT Calculations

Quantum chemical calculations were conducted using the ORCA (version 6.0) program package [34]. Optimized molecular structures were obtained using DFT [53] with the B3LYP functional [36, 38, 39, 54] in combination with the LANL2DZ basis set [55, 56, 57, 58].

Frequency calculations were performed for all optimized geometries, confirming their stability by the absence of imaginary frequencies. Cartesian force constants extracted from the ORCA Hessian file were converted to internal coordinate force constants using the FCT program [59, 60, 61, 62]. Visualization of molecular structures was carried out using the JMOL program [63]. Optimized geometries of the complexes are presented in Figure 12, while calculated bond lengths and force constants are summarized in Table S9. For clarity, molecular structures in the text, Table S9, and Figure 12 are denoted as S followed by a number.

## 4 Conclusion

The novel sodium tetrachlorido‐/azidoaurate(III) dihydrates Na[AuCl_4–_ *_x_* (N_3_)*_x_*]·2H_2_O (*x* = 0, 1, 2, 3, 4) provide the first example of a complete series of gradual substitution on square planar complex anions to be described. Transparent yellow to dark orange single crystals of these phases were synthesized by reaction of NaN_3_ and AuCl_3_ or HAuCl_4_ in different molar ratios from aqueous solution. Controlled dehydration of Na[Au(N_3_)_4_]·2H_2_O led to Na[Au(N_3_)_4_]·H_2_O and Na[Au(N_3_)_4_] in form of orange microcrystalline powders, the latter being a highly explosive material. Predominant structural features of all phases are discrete anions [AuCl_4–_ *_x_* (N_3_)*_x_*]^–^ with gold in an ordered square planar coordination of azide/chloride anions. Vibrational spectra are analyzed in detail based on the idealized point group *C*_4_ *_h_* of the spectroscopically relevant unit [Au(N_3_)_4_]^–^ and show good agreement with other azidoaurates(III).

## Conflicts of Interest

The authors declare no conflict of interests.

## Supporting information



The authors have cited additional references within the Supporting Information [1–27]. **Supporting File: 1** chem70886‐sup‐0001‐SuppMat.docx

**Supporting File: 2** chem70886‐sup‐0002‐data.zip
