# Supplementary material for: Sodium Tetraazidoaurate(III)—From Na[AuCl4]·2H2O to Na[Au(N3)4] and Beyond One Step at a Time
Source: Chemistry. 2026 Mar 26;32(22):e02800. doi: 10.1002/chem.202502800 (PMC13250357; doi:10.1002/chem.202502800)
Supplement: Supplementary file 1 — The authors have cited additional references within the Supporting Information [1–27]. Supporting File: 1 chem70886‐sup‐0001‐SuppMat.docx [file CHEM-32-e02800-s002.docx]

Supporting Information
©Wiley-VCH 2019
69451 Weinheim, Germany

Sodium Tetraazidoaurate(III) – from Na[AuCl_4_]·2H_2_O to Na[Au(N_3_)_4_] and beyond one step at a time

Mehmet Somer,*^[a]^ Joannis Psilitelis,^[b]^ Raul Cardoso-Gil,^[c]^ Thomas Doert,^[d]^ Franziska Jach,^[e]^ Ayberk Yılmaz,^[f]^ Alexander Ovchinnikov,^[d]^ Yurii Prots,^[c]^ Helge Rosner,^[g]^ Marcus P. Schmidt,^[c]^ Peter Höhn*^[c]^

**Abstract:** The novel sodium tetrachlorido-/azidoaurate(III) dihydrates Na[AuCl_4–_*_x_*(N_3_)*_x_*]·2H_2_O (*x* = 0, 1, 2, 3, 4) provide the first example of a complete series of gradual substitution on square planar complex anions to be described. Transparent yellow to dark orange single crystals of these phases were synthesized by reaction of NaN_3_ and AuCl_3_ or HAuCl_4_ in different molar ratios from aqueous solution. Controlled dehydration of Na[Au(N_3_)_4_]·2H_2_O led to Na[Au(N_3_)_4_]·H_2_O and Na[Au(N_3_)_4_] in form of orange microcrystalline powders, the latter being a highly explosive material. Predominant structural features of all phases are discrete anions [AuCl_4–_*_x_*(N_3_)*_x_*]^–^ with gold in an ordered square planar coordination of azide/chloride anions. Vibrational spectra show good agreement with other known azidoaurates(III).

DOI: 10.1002/anie.2016XXXXX

Table of Contents

[Table of Contents 2](#_Toc218858534)

[Experimental Procedures 4](#_Toc218858535)

[**Synthesis** 4](#_Toc218858536)

[**Figure S1**: Single crystals of selected phases *A*[AuCl_4–_*_x_*(N_3_)*_x_*]·*n*H_2_O. a) Na[AuCl_4_]·2H_2_O, b) Na[AuCl_3_(N_3_)]·2H_2_O, c) Na[AuCl_2_(N_3_)_2_]·2H_2_O, d) Na[AuCl(N_3_)_3_]·2H_2_O, e,f) Na[Au(N_3_)_4_]·2H_2_O, g) K[AuCl_2_(N_3_)_2_], h) K[Au(N_3_)_4_] 4](#_Toc218858537)

[**Figure S2**: Relative change of the lattice parameters *a*, *b*, *c* with cell volume for Na[AuCl_4–_*_x_*(N_3_)*_x_* (0 ≤ *x* ≤ 1). Cell volume increases with *x*(N_3_). 4](#_Toc218858538)

[**X-ray diffraction** 5](#_Toc218858539)

[**Figure S3**: Reciprocal lattice of Na[AuCl_2_(N_3_)_2_]·2H_2_O in a projection along [001]* reconstructed from the collected data set. White and red parallelograms indicate the reciprocal lattices of two twin domains connected by rotation around the reciprocal vector [1 -1/4 0]*. In direct space, this corresponds to a rotation about the [100] axis and the twin law (1 0 0 -1/2 -1 0 0 0 -1). 6](#_Toc218858540)

[**Figure S4**: Detail of the X-ray powder diffraction pattern of Na[Au(N_3_)_4_]·H_2_O: indexing in *P*2_1_2_1_2_1_ (left) and *Pnma* (right) shows only minute, but significant changes (red arrows), which do not correspond to peak positions in *Pnma*. 7](#_Toc218858541)

[**Figure S5**: Rietveld refinement of the Na[Au(N_3_)_4_]·H_2_O sample. Experimental data, calculated intensities and difference curve are shown in black, red, and blue, respectively. Tick marks indicate Bragg peak positions for (from top to bottom) Na[Au(N_3_)_4_]·H_2_O (90.0(8) wt. %), NaCl (6.5(5) wt. %), and Na[Au(N_3_)_4_]·2H_2_O (3.5 wt. %). 8](#_Toc218858542)

[**Figure S6a-d**: High temperature powder diffraction measurement of a) Na[AuCl_4_]·2H_2_O, b) Na[AuCl_3_(N_3_)]·2H_2_O, c) Na[AuCl_2_(N_3_)_2_]·2H_2_O, and d) Na[AuCl(N_3_)_3_]·2H_2_O. Whereas in Na[AuCl_4_]·2H_2_O and Na[AuCl_3_(N_3_)]·2H_2_O no changes are evident, dehydration starts in Na[AuCl_2_(N_3_)_2_]·2H_2_O at 92 °C and in Na[AuCl_3_(N_3_)]·2H_2_O at 82 °C. Cooling to room temperature reestablishes the dihydrated phases, although some decomposition is evident from increased intensity of Au (red) and NaCl (green) peaks. 9](#_Toc218858543)

[**Figure S6e**: High temperature powder diffraction measurement of Na[Au(N_3_)_4_]·2H_2_O. The transformation to Na[Au(N_3_)_4_]·H_2_O (green) starts at 345 K and is not finished at the end of the measurement at 360 K, when Na[Au(N_3_)_4_] (red) already begins to appear. 10](#_Toc218858544)

[**Differential Scanning Calorimetry** 11](#_Toc218858545)

[**Thermal analysis** 11](#_Toc218858546)

[**Vibrational spectroscopy** 11](#_Toc218858547)

[**DFT calculations** 11](#_Toc218858548)

[Details of the structure description 12](#_Toc218858549)

[**Table S1.** Crystallographic data for Na[Au(Cl_4–_*_x_*(N_3_)*_x_*]·2H_2_O (*x* = 0, 1, 2, 3, 4). 12](#_Toc218858550)

[**Table S2a.** Atomic coordinates and isotropic displacement parameters [Å^2^] for Na[AuCl_4_]·2H_2_O. Standard deviations are given in parentheses. 13](#_Toc218858551)

[**Table S3a.** Anisotropic displacement parameters [Å^2^] for Na[AuCl_4_]·2H_2_O. Standard deviations are given in parentheses. 13](#_Toc218858552)

[**Table S4a.** Selected interatomic distances [Å] and bond angles [°] in Na[AuCl_4_]·2H_2_O with their multiplicity (*n*). Standard deviations are given in parentheses. 14](#_Toc218858553)

[**Table S2b.** Atomic coordinates and isotropic displacement parameters [Å^2^] for Na[AuCl_3_N_3_]·2H_2_O. Standard deviations are given in parentheses. 15](#_Toc218858554)

[**Table S3b.** Anisotropic displacement parameters [Å^2^] for Na[AuCl_3_N_3_]·2H_2_O. Standard deviations are given in parentheses. 15](#_Toc218858555)

[**Table S4b.** Selected interatomic distances [Å] and bond angles [°] in Na[AuCl_3_N_3_]·2H_2_O with their multiplicity (*n*). Standard deviations are given in parentheses. 16](#_Toc218858556)

[**Table S2c.** Atomic coordinates and isotropic displacement parameters [Å^2^] for Na[AuCl_2_(N_3_)_2_]·2H_2_O. Standard deviations are given in parentheses. 17](#_Toc218858557)

[**Table S3c.** Anisotropic displacement parameters [Å^2^] for Na[AuCl_2_(N_3_)_2_]·2H_2_O. Standard deviations are given in parentheses. 17](#_Toc218858558)

[**Table S4c.** Selected interatomic distances [Å] and bond angles [°] in Na[AuCl_2_(N_3_)_2_]·2H_2_O with their multiplicity (*n*). Standard deviations are given in parentheses. 18](#_Toc218858559)

[**Table S2d.** Atomic coordinates and isotropic displacement parameters [Å^2^] for Na[AuCl(N_3_)_3_]·2H_2_O. Standard deviations are given in parentheses. 19](#_Toc218858560)

[**Table S3d.** Anisotropic displacement parameters [Å^2^] for Na[AuCl(N_3_)_3_]·2H_2_O. Standard deviations are given in parentheses. 19](#_Toc218858561)

[**Table S4d.** Selected interatomic distances [Å] and bond angles [°] in Na[AuCl(N_3_)_3_]·2H_2_O with their multiplicity (*n*). Standard deviations are given in parentheses. 20](#_Toc218858562)

[**Table S2e.** Atomic coordinates and isotropic displacement parameters [Å^2^] for Na[Au(N_3_)_4_]·2H_2_O. Standard deviations are given in parentheses. 21](#_Toc218858563)

[**Table S3e.** Anisotropic displacement parameters [Å^2^] for Na[Au(N_3_)_4_]·2H_2_O. Standard deviations are given in parentheses. 22](#_Toc218858564)

[**Table S4e.** Selected interatomic distances [Å] and bond angles [°] in Na[Au(N_3_)_4_]·2H_2_O with their multiplicity (*n*). Standard deviations are given in parentheses. 23](#_Toc218858565)

[**Table S5.** Crystallographic data for Na[Au(N_3_)_4_]·*n*H_2_O (*n* = 2, 1, 0). 24](#_Toc218858566)

[**Table S8a.** Selected interatomic distances [Å] and bond angles [°] in Na[Au(N_3_)_4_]·H_2_O with their multiplicity (*n*). Standard deviations are given in parentheses. 26](#_Toc218858567)

[**Table S6b.** Atomic coordinates and isotropic displacement parameters [Å^2^] for Na[Au(N_3_)_4_]. Standard deviations are given in parentheses. 27](#_Toc218858568)

[**Table S7b.** Anisotropic displacement parameters [Å^2^] for Na[Au(N_3_)_4_]. Standard deviations are given in parentheses. 27](#_Toc218858569)

[**Table S8b.** Selected interatomic distances [Å] and bond angles [°] in Na[Au(N_3_)_4_] with their multiplicity (*n*). Standard deviations are given in parentheses. 28](#_Toc218858570)

[**Hydrogen bonds** 29](#_Toc218858571)

[**Figure S7.** The environment of azidoaurate anion [Au(N_3_)_4_]^–^ in Na[Au(N_3_)_4_]_2_·H_2_O. View perpendicular (left) and through the AuN_4_ plane (right). Contacts to O with distances 289 pm < *d*(N–O) < 339 pm (blue dotted lines) indicate potential moderate or weak hydrogen bonds.^[22]^ 29](#_Toc218858572)

[Spectroscopy 30](#_Toc218858573)

[**Vibrational Spectra of Na[AuCl_4–_*_x_*(N_3_)*_x_*]⋅2H_2_O (*x* = 1, 2, 3)** 30](#_Toc218858574)

[**Figure S8.** Vibrational spectra of Na[AuCl_4–_*_x_*(N_3_)*_x_*]·2H_2_O (*x* = 0, 1, 2, 3, 4): a) Raman spectra in the region 100–2500 cm^–1^, b) Detail of Raman spectra in the region 100–600 cm^–1^, c) IR spectra in the region 400–4000 cm^–1^. Intensity (Raman) and transmission (IR-ATR) in arbitrary units. For experimental conditions see text. 32](#_Toc218858575)

[**Figure S9.** Vibrational spectra of Na[Au(N_3_)_4_]·2H_2_O. Intensity (Raman) and transmission (IR-ATR) in arbitrary units. 33](#_Toc218858576)

[**Figure S10.** IR spectra of Na[Au(N_3_)_4_]·2H_2_O, Na[Au(N_3_)_4_]·H_2_O and Na[Au(N_3_)_4_]. Transmission (IR-ATR) in arbitrary units. 33](#_Toc218858577)

[34](#_Toc218858578)

[**Figure S11.** IR spectra of mixtures of Na[AuCl_4–_*_x_*(N_3_)*_x_*]·2H_2_O (0 ≤ *x* ≤ 4). Transmission in arbitrary units. 34](#_Toc218858579)

[**Figure S12.** IR spectra (in non-standard representation with transmission going down) of a heating measurement up to 125 °C of a sample of Na[Au(N_3_)_4_]·2H_2_O (yellow) showing the transition to Na[Au(N_3_)_4_]·H_2_O (red) and Na[Au(N_3_)_4_] (blue) and back. Mixed colors (orange, pink, green) indicate presence of multiple phases. Na[Au(N_3_)_4_]·H_2_O is not observed upon cooling. 35](#_Toc218858580)

[DFT calculations 35](#_Toc218858581)

[**Table S9.** Bond lengths (Å) and force constants (Ncm^–1^) (B3LYP/LANL2DZ) within the Na[AuCl_4–_*_x_*(N_3_)*_x_*] series 36](#_Toc218858582)

[References 37](#_Toc218858583)

[Author Contributions 37](#_Toc218858584)

Experimental Procedures

**Synthesis**

Due to the highly explosive nature of the title compounds, all handling during the experiments was carried out under extreme safety precautions (protective shields, goggles and gloves) using minute amounts of sample. It is essential to wear protective gear and to prevent shock, heat, and intense radiation on samples. For investigation, the fully prepared materials (mg amounts) were transported in safe containers between the different measurement facilities without use of public services.

White solid NaN_3_ (Merck, 99.9 %, recrystallized in water) and AuCl_3_ (Thermo Scientific, 99.99%) were educts to obtain all phases.

In all reactions, upon combining the different liquids, the color of the aqueous solution instantaniously changed to a deep orange. The obtained mixture was kept in a large crystallizing dish for 12 to 60 hours in a slightly cold environment (~ 5 ^°^C) to obtain crystals of the desired compound. After few days, large translucent orange-red single crystals (Figure S1) suitable to be examined by X-Ray single crystal diffraction as well as crystalline agglomerates in form of brittle and often twinned needle-like plates were obtained upon evaporization of the solvent. Most of the colorless by-products, presumabely unreacted NaCl, NaN_3_, and other phases, could be mechanically removed. Repeated recrystallization in first methanol and then water not only removed the remaining traces of impurities, but also improved crystal quality.


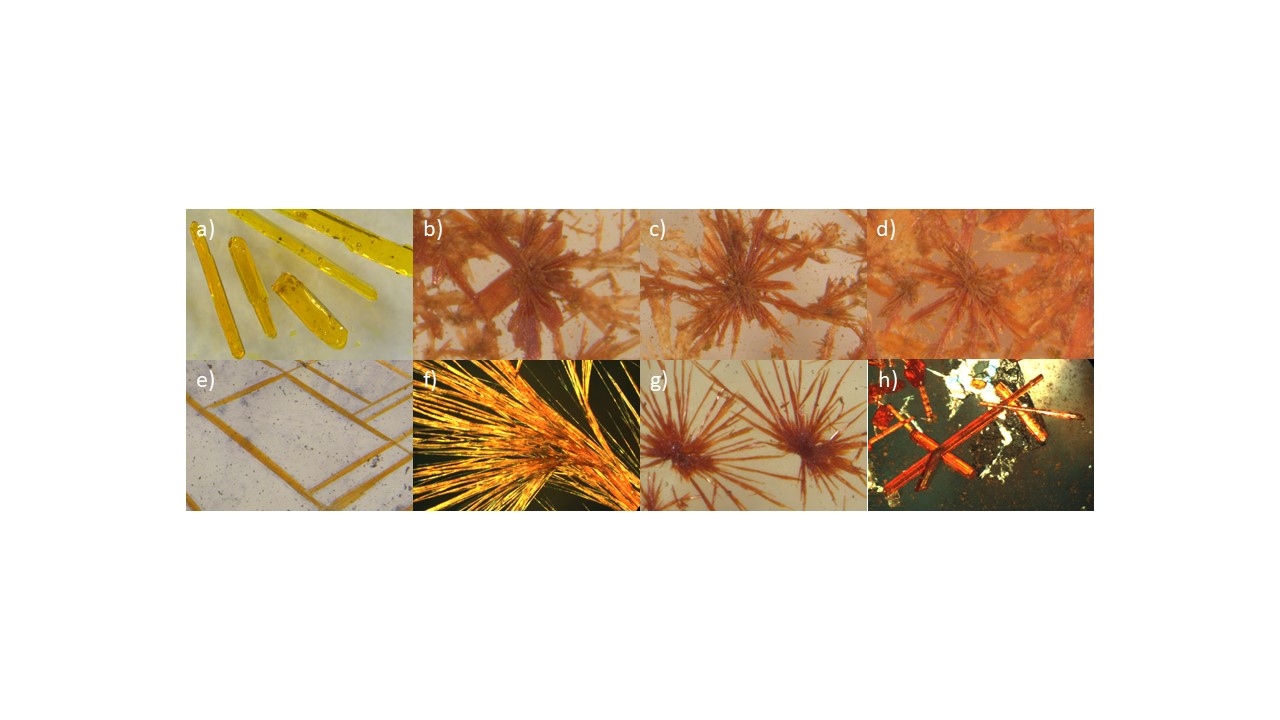
The crystals could be handled safely and even ground to fine powders of 25 my size upon being careful not to exert too high pressure, however, they were highly explosive when dry, exposed to high pressure, heat, or intense radiation. Due to the instable nature of the samples, no chemical analyses were performed.
The use of HAuCl_4_ (Sigma Aldrich, 99.9%) instead of AuCl_3_ gave similar results. Temperature treatment experiments were performed using either a heat plate or an ice bath in the range of 273 K – 323 K without improving results.

**Figure S1**: Single crystals of selected phases *A*[AuCl_4–_*_x_*(N_3_)*_x_*]·*n*H_2_O. a) Na[AuCl_4_]·2H_2_O, b) Na[AuCl_3_(N_3_)]·2H_2_O, c) Na[AuCl_2_(N_3_)_2_]·2H_2_O, d) Na[AuCl(N_3_)_3_]·2H_2_O, e,f) Na[Au(N_3_)_4_]·2H_2_O, g) K[AuCl_2_(N_3_)_2_], h) K[Au(N_3_)_4_]

**Na[AuCl_4–_*_x_*(N_3_)*_x_*****]·2H_2_O (*x* = 1, 2, 3, 4)**: in all experiments an excess of NaN_3_ had to be employed to obtain specimen containing only one single azidochloridoaurate phase. Na[AuCl_4_]·2H_2_O was prepared from NaCl and AuCl_3_ in aqueous solution according to literature.^[1]^


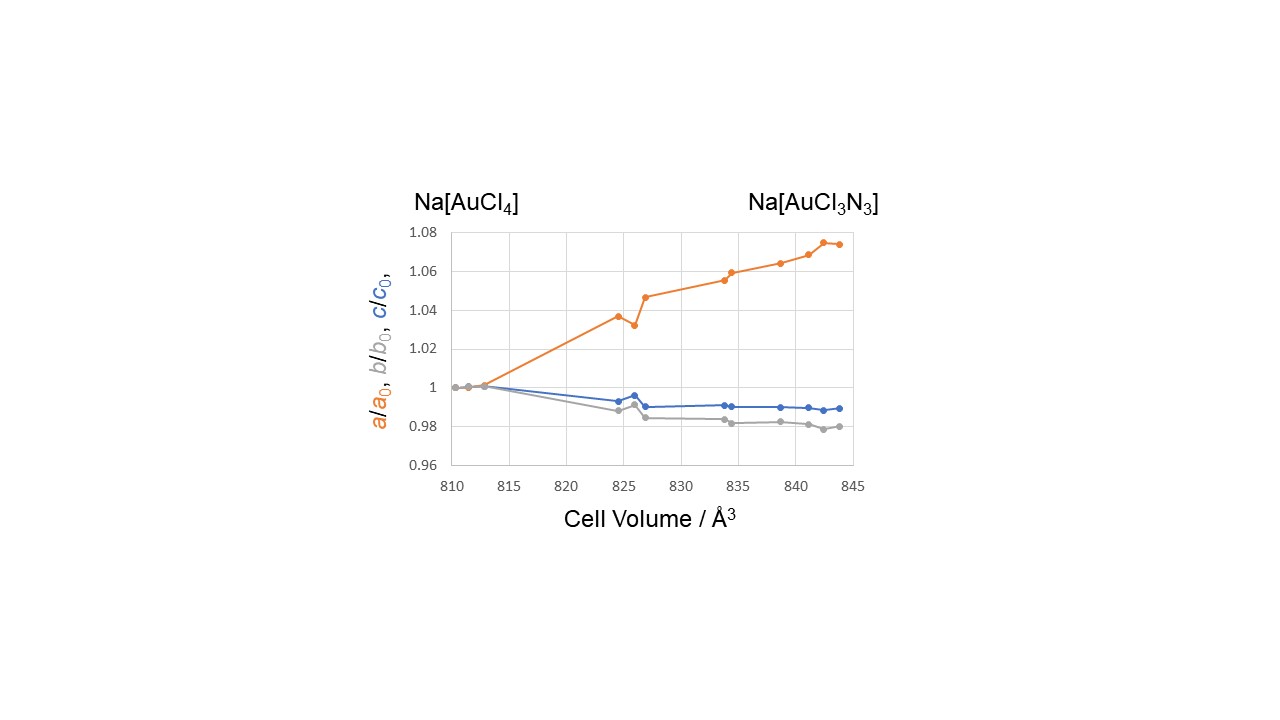
**Na[AuCl_3_(N_3_)]·2H_2_O** was obtained from mixtures of 0.011 mg / 0.169 mmol NaN_3_ and 0.035 mg / 0.115 mmol AuCl_3_, corresponding to a molar ratio of 1.47 : 1. The starting materials were dissolved in 1 ml water each, mixed and evaporated in air. In the course of our investigations, it was observed, that one of the chloride species of the [AuCl_4_]^–^ can be exchanged by an azide species leading to a solution series **Na[AuCl_4–_*_x_*(N_3_)*_x_*]·2H_2_O** **(0 ≤ *x* ≤ 1).** According to X-ray powder diffraction data (Figure S2) neither further ordering phenomena nor a miscibility gap are expected.

**Figure S2**: Relative change of the lattice parameters *a*, *b*, *c* with cell volume for Na[AuCl_4–_*_x_*(N_3_)*_x_* (0 ≤ *x* ≤ 1). Cell volume increases with *x*(N_3_).

Phase-pure **Na[AuCl_2_(N_3_)_2_]·2H_2_O** was prepared from mixtures of 0.018 mg / 0.277 mmol NaN_3_ and 0.035 mg / 0.115 mmol AuCl_3_, corresponding to a molar ratio of 2.40 : 1. The starting materials were dissolved in 1 ml water each, mixed and evaporated in air. No phase width was observed.
Synthesis of **Na[AuCl(N_3_)_3_]·2H_2_O** succeeded from mixtures of 0.025 mg / 0.385 mmol NaN_3_ and 0.035 mg / 0.115 mmol AuCl_3_, corresponding to a molar ratio of 3.33 : 1. The starting materials were dissolved in 1 ml water each, mixed and evaporated in a crystallizing dish. No phase width was observed.
To synthesize **Na[Au(N_3_)_4_]·2H_2_O**, the reaction of NaN_3_ and AuCl_3_ in molar ratio 6.67 : 1 gave best results. The starting materials, NaN_3_ (0.050 g / 0.769 mmol) and AuCl_3_ (0.035 g / 0.115 mmol, Sigma Aldrich, 99.99%), were dissolved in 1 ml water each; the solutions were reacted with a significant excess of alkali azide according to (1):
(1) AuCl_3_ + 6.67 NaN_3_ → Na[Au(N_3_)_4_] + 2.67 NaN_3_ + 3 NaCl (1)
Purification of **Na[Au(N_3_)_4_]·2H_2_O** succeeded by dissolving the crystalline product in methanol and subsequent evaporation of the solvent; crystal quality significantly improved employing one further dissolving/crystallization cycle from water. No phase width was observed.
Upon dissolving any of the reaction products above in water, the addition of small amounts of NaN_3_ or AuCl_3_ led to recrystallisation of mixtures also containing the respective neighboring phases richer in azide or chloride, respectively; in all such experiments only mixtures containing exactly two neighboring phases were observed.
No phases with a higher content of crystal water were obtained.
Upon heating, removal of crystal water proceeds in one step in **Na[AuCl_4–x_(N_3_)_x_]·2H_2_O** **(x = 0, 1, 2, 3)** at temperatures between 119 °C and 78 °C; the resulting phases were not further investigated.

Exhaustive experiments to remove crystal water, either by slow heating or by cautious evacuation, were performed with sodium tetraazidoaurate dihydrate Na[Au(N_3_)_4_]·2H_2_O. The water-free phase **Na[Au(N_3_)_4_]** is obtained easily either by heating to 80 °C in a drying oven for at least 1 h, heating to 68 °C in a tube furnace for 72 h, or evacuation in the antechamber of a glove box for at least 1 h. The samples obtained by these methods are highly sensitive and may explode upon handling. Putting completely dehydrated water-free samples outside the glovebox into air led to rehydration to Na[Au(N_3_)_4_]·2H_2_O within two minutes without evidence of formation of intermediate phases.
The emergence of the monohydrate phase **Na[Au(N_3_)_4_]·H_2_O** was first observed during DSC investigations of Na[Au(N_3_)_4_]·2H_2_O in measuring devices intended for quality control of explosives, Different attempts were made to prepare single-phase samples of the monohydrate **Na[Au(N_3_)_4_]·H_2_O**, most of them unsuccessful: short-time heating as well as short-term evacuation of finely ground samples of **Na[Au(N_3_)_4_]·2H_2_O** in an antechamber for about 10 minutes led to mixtures of **Na[Au(N_3_)_4_]·2H_2_O**, **Na[Au(N_3_)_4_]·H_2_O**, and **Na[Au(N_3_)_4_]**. Due to fast reaction with the environment, mixing equal amounts of **Na[Au(N_3_)_4_]·2H_2_O** and **Na[Au(N_3_)_4_]** proved also unsuccessful. The best results to obtain **Na[Au(N_3_)_4_]·H_2_O** were achieved filling finely ground **Na[Au(N_3_)_4_]·2H_2_O** in a glass capillary of 0.5 mm diameter, heating the open capillary in a tube furnace at 68 °C for 6 h, sealing the capillary and employing two further heating cycles of 12 h each about 24 h apart.
Due to the highly explosive nature of the title compounds, the content of N and O in the bulk material could not be determined quantitatively by carrier gas hot-extraction technique. In preliminary experiments with sample amounts below 1 mg, heavy explosions threatened to damage not only the crucibles used for the analysis, but also the analyzer itself.

**X-ray diffraction**

For all phases containing two crystal water – **Na[AuCl_4–_*_x_*(N_3_)*_x_*]·2H_2_O (*x* = 0, 1, 2, 3, 4)** – single or twinned crystals could be obtained, which were suitably sized for X-ray single crystal diffraction. They were glued to the tips of glass fibres for X-ray diffraction intensity data collection at room temperature on a Rigaku AFC7 automatic diffractometer equipped with a Saturn 724+ CCD detector and Mo*K*α radiation using a graphite-monochromator and applying the φ oscillation scan technique. All crystal structures were solved from single crystal data using SHELXS-2018 and refined using the full-matrix least-squares procedure with the SHELXL-2018 software package, see **Tables S1 – S3** for details.^[2-3]^ In most cases, it was only possible to localize and refine hydrogen positions using heavy restraints. In accordance with the solid solution series **Na[AuCl_4–_*_x_*(N_3_)*_x_*]·2H_2_O (0 ≤ *x* ≤ 1)**, the single crystal selected as Na[AuCl_3_(N_3_)]·2H_2_O proved to be **Na[AuCl_3.11(2)_(N_3_)_0.89(2)_]·2H_2_O**. The crystal structure of **Na[AuCl_2_(N_3_)_2_]·2H_2_O** was established from a twinned specimen, the twinning scheme is shown in Figure S3. In all phases, the highest peaks in the residual electron difference density map are located about 0.8 Å above and below the Au*X*_4_-plane (*X* = Cl, N_3_) and have no structural significance.

**
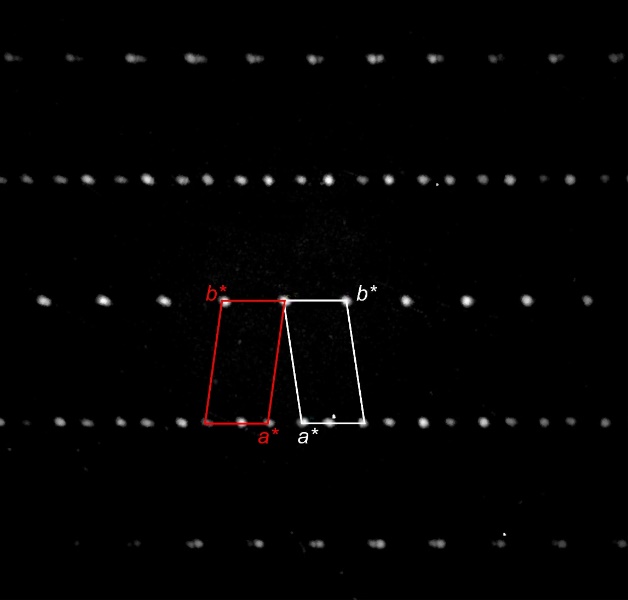
**

**Figure S3**: Reciprocal lattice of Na[AuCl_2_(N_3_)_2_]·2H_2_O in a projection along [001]* reconstructed from the collected data set. White and red parallelograms indicate the reciprocal lattices of two twin domains connected by rotation around the reciprocal vector [1 -1/4 0]*. In direct space, this corresponds to a rotation about the [100] axis and the twin law (1 0 0 -1/2 -1 0 0 0 -1).

No single crystals could be obtained for the phases **Na[Au(N_3_)_4_]·H_2_O** and **Na[Au(N_3_)_4_]**, so powder X-ray diffraction data had to be employed for structure solution and refinement. For the measurement of **Na[Au(N_3_)_4_]·H_2_O** and **Na[Au(N_3_)_4_]** on a STOE STADI P powder diffractometer, glass capillaries (outside-Ø = 0.3 or 0.5 mm; wall thickness = 0.01mm) were prepared using the methods described above; for Guinier measurements of **Na[Au(N_3_)_4_]**, finely ground **Na[Au(N_3_)_4_]·2H_2_O** was placed on a Vaseline-coated Kapton foil on a flatbed sample holder, evacuated in the antechamber of a glovebox for 1h, placed into the glovebox for 1 h and covered with a second Kapton foil prior to measurement to prevent reaction with water and air.

The X-ray powder diffraction experiment for crystal structure refinement was performed using a Stoe Stadi MP (STOE & Cie GmbH, Darmstadt, Germany) in Debye-Scherrer geometry. The diffractometer is equipped with a DECTRIS MYTHEN2 1K silicon strip detector (DECTRIS AG, Baden-Daettwil, Switzerland) and operates using pure Cu-Kα_1_ radiation (λ = 1.54056 Å, curved germanium (111) Johann-type Monochromator). To improve the statistics of the data points, the final powder pattern is the sum of three individual intensity datasets collected within the angular range of 5.00° ≤ 2θ ≤ 110° (scan step = 0.3°, time pro step 60 s, *t*_total_ ≈ 20 h).

Attempts to collect powder synchrotron diffraction data failed; upon radiating the powder samples placed in capillaries, the samples very rapidly decomposed.

Solution and refinement of the crystal structure of Na[Au(N_3_)_4_] proceeded straightforward in JANA2020,^[4]^ whereas Na[Au(N_3_)_4_]·H_2_O provided several challenges. The multi-phase Na[Au(N_3_)_4_]·H_2_O sample contained small amounts of Na[Au(N_3_)_4_]·2H_2_O and NaCl. Indexing of the reflections belonging to the major phase yielded an orthorhombic unit cell with reflections conditions indicating the *Pnma* space group. Close analysis of the pattern refined using the LeBail procedure showed that several weak reflections could not be described in space group *Pnma*, but were indexed successfully when the symmetry was lowered to *P*2_1_2_1_2_1_ (Figure S4).

**
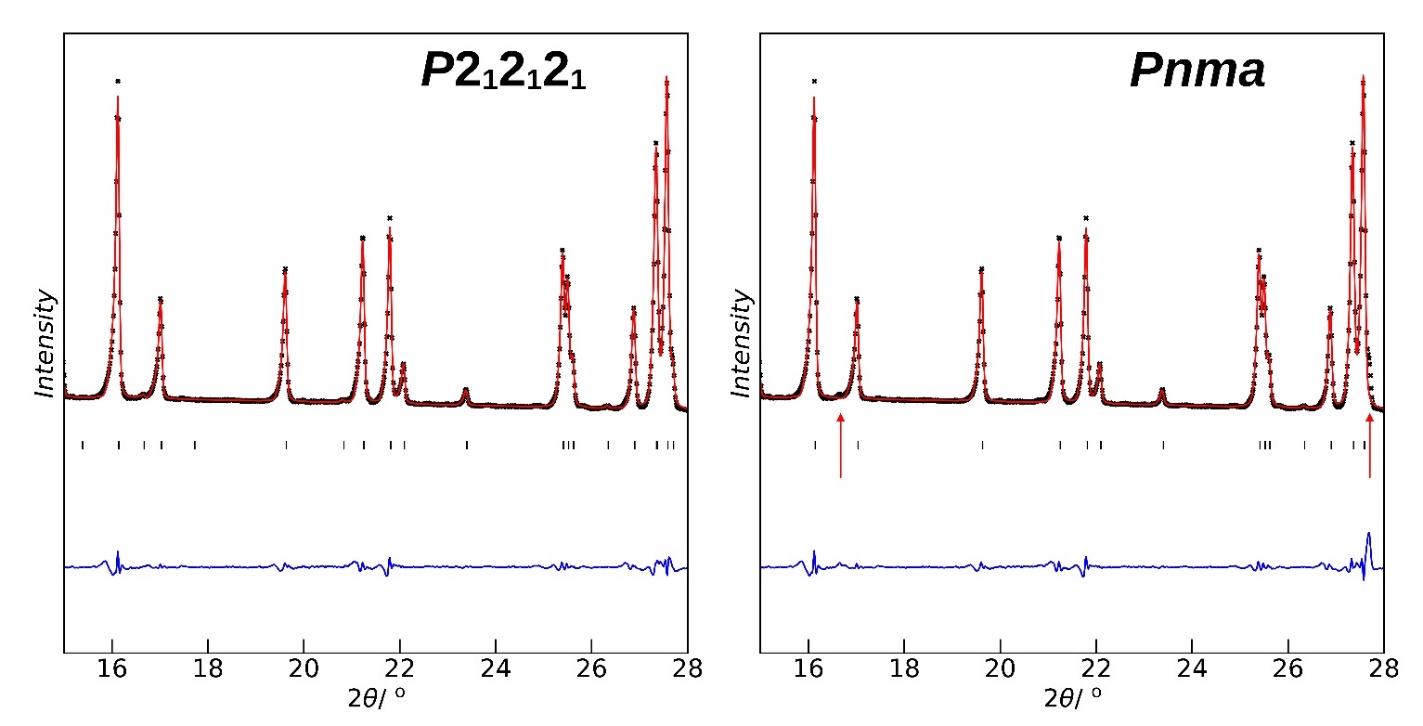
**

**Figure S4**: Detail of the X-ray powder diffraction pattern of Na[Au(N_3_)_4_]·H_2_O: indexing in *P*2_1_2_1_2_1_ (left) and *Pnma* (right) shows only minute, but significant changes (red arrows), which do not correspond to peak positions in *Pnma*.

Crystal structure solution with charge-flipping algorithm as implemented in Superflip within the JANA2020 package,^[4]^ provided positions of the heavy atoms Au and Na. The locations of the Au and Na atoms were consistent with space group *Pnma*, but, for further refinements, space group *P*2_1_2_1_2_1_ was retained. After initial refinements of the heavy atom positions, four inner nitrogen atoms forming a square-planar coordination around the Au atom, as well as the oxygen atom of the water molecule were located by difference Fourier mapping. However, further unconstrained refinement resulted in distortion of the square shape in the [AuN_4_] unit. The observed distortion can be explained by the fact that the electron density corresponding to the N atoms is very low and poorly structured, apparently due to the influence of the high electron density of the Au atoms. Consequently, the four nitrogen atoms deviate from their expected positions during refinement in order to describe the smeared electron density of the azide anions. To circumvent this issue, soft restraints were introduced for the Au–N bond distances, N–Au–N bond angles and the torsion angles involving the N atoms to keep the planar geometry of the [AuN_4_] unit. After additional refinement cycles, further N positions were located following difference Fourier mapping. For all newly found N positions, additional restraints were applied to ensure that the N–N distances and N–N–N angles are within the expected ranges. The refinement and Fourier mapping cycles were repeated until all nitrogen atoms had been found. In the final cycles of Rietveld refinement, isotropic displacement parameters were kept equal within the groups of inner, middle, and terminal N atoms, respectively.

We also employed an alternative approach for crystal structure determination of Na[Au(N_3_)_4_]·H_2_O by using direct space crystal structure solution in FOX.^[5]^ In this case, a global optimization of the positions of the selected structural building blocks was carried out, which allowed avoiding potential local minima. Given the unit cell volume and the fact that any atomic position in space group *P*2_1_2_1_2_1_ has a multiplicity of 4, the structure of Na[Au(N_3_)_4_]·H_2_O must contain one symmetry unique Na and O site, as well as one symmetry unique [Au(N_3_)_4_] unit (note that H atoms usually cannot be detected by X-ray powder diffraction and were neglected here). The [Au(N_3_)_4_] unit was introduced as a flexible molecule with soft restraints similar to those described above for the JANA2020 refinement. Global optimization was performed in the parallel tempering mode. More than 30 runs with 5·10^7^ trials per run were completed and resulted in the same crystal structure as the one obtained in the JANA2020 refinement above, thereby confirming the structural model.

Chrystallographic data and further details for Na[Au(N_3_)_4_]·H_2_O and Na[Au(N_3_)_4_] are given in Tables S4–S6.

Deposition Numbers <url href="[https://www.ccdc.cam.ac.uk/services/structures?id=doi:10.1002/chem.202502800 <https://track.editorialmanager.com/CL0/https:%2F%2Fwww.ccdc.cam.ac.uk%2Fservices%2Fstructures%3Fid=doi:10.1002%2Fchem.202502800/1/010f0199bdc059f4-8365e4b9-c6d6-4161-ae1c-93fc83c14950-000000/prRLDNLPIKzGaeQ6X7RwFoNAXyR8SltGlySivZcy3pU=231>](https://www.ccdc.cam.ac.uk/services/structures?id=doi:10.1002/chem.202502800%20%3chttps://track.editorialmanager.com/CL0/https:%2F%2Fwww.ccdc.cam.ac.uk%2Fservices%2Fstructures%3Fid=doi:10.1002%2Fchem.202502800/1/010f0199bdc059f4-8365e4b9-c6d6-4161-ae1c-93fc83c14950-000000/prRLDNLPIKzGaeQ6X7RwFoNAXyR8SltGlySivZcy3pU=231%3e%20) "> CSD-2486294 (for Na[AuCl_4_]·2H_2_O), CSD-2486295 (for Na[AuCl_3_(N_3_)]·2H_2_O), CSD-2486289 (for Na[AuCl_2_(N_3_)_2_]·2H_2_O), CSD-2486285 (for Na[AuCl(N_3_)_3_]·2H_2_O), CSD-2486284 (for Na[Au(N_3_)_4_]·2H_2_O), CSD-2486383 (for Na[Au(N_3_)_4_]·H_2_O), and CSD-2486567 (for Na[Au(N_3_)_4_]) contain the supplementary crystallographic data for this paper. These data are provided free of charge by the joint Cambridge Crystallographic Data Centre and Fachinformationszentrum Karlsruhe <url href="[http://www.ccdc.cam.ac.uk/structures <http://track.editorialmanager.com/CL0/http:%2F%2Fwww.ccdc.cam.ac.uk%2Fstructures/1/010f0199bdc059f4-8365e4b9-c6d6-4161-ae1c-93fc83c14950-000000/tNRLlHj2imbToi1xYm0kiaSs2ggbCftaWEHVInsm29g=231>](http://www.ccdc.cam.ac.uk/structures%20%3chttp:/track.editorialmanager.com/CL0/http:%2F%2Fwww.ccdc.cam.ac.uk%2Fstructures/1/010f0199bdc059f4-8365e4b9-c6d6-4161-ae1c-93fc83c14950-000000/tNRLlHj2imbToi1xYm0kiaSs2ggbCftaWEHVInsm29g=231%3e%20) ">Access Structures service</url>.

Graphical representations of the structure were created in Diamond.^[6]^


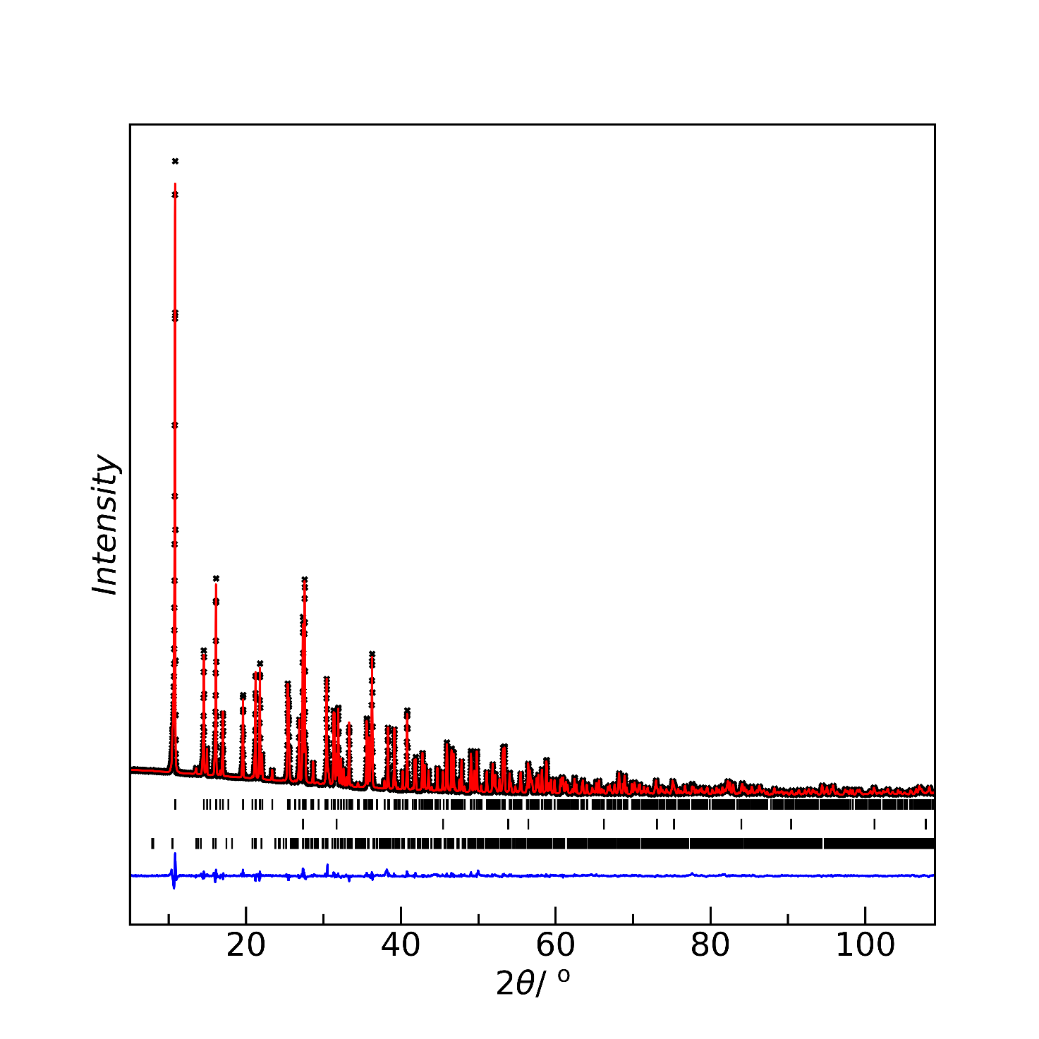

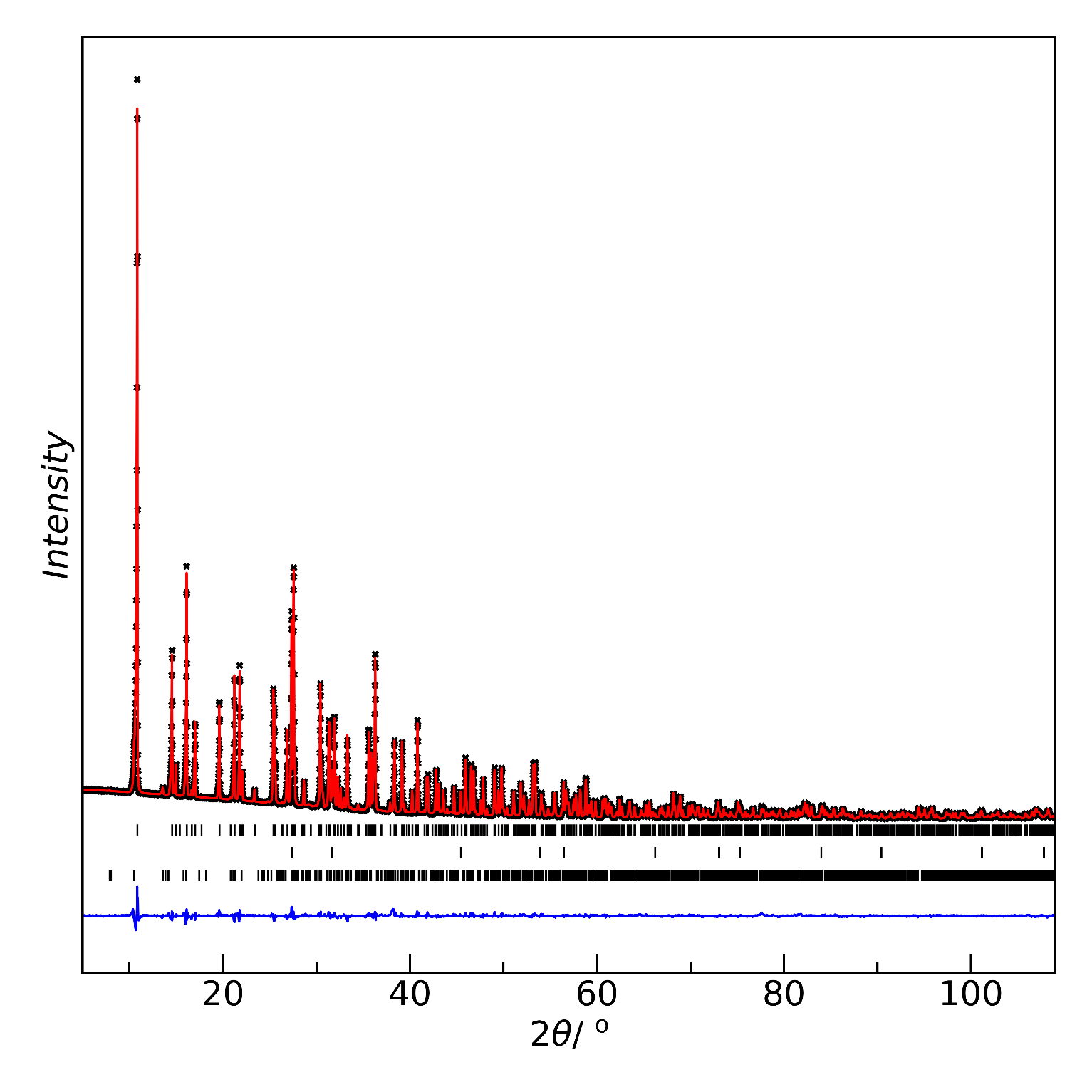


**Figure S5**: Rietveld refinement of the Na[Au(N_3_)_4_]·H_2_O sample. Experimental data, calculated intensities and difference curve are shown in black, red, and blue, respectively. Tick marks indicate Bragg peak positions for (from top to bottom) Na[Au(N_3_)_4_]·H_2_O (90.0(8) wt. %), NaCl (6.5(5) wt. %), and Na[Au(N_3_)_4_]·2H_2_O (3.5 wt. %).

To investigate the structural changes in **Na[Au(N_3_)_4_]·2H_2_O** *in situ*, temperature-dependent powder X-ray diffraction data were collected on a STADI P diffractometer (Stoe & Cie, Darmstadt, Germany) using Ge-monochromatized Cu*K*_α1_ radiation and a Mythen detector (Dectris, Baden, Switzerland) in Debye-Scherrer geometry. Heating and cooling was realized using an N_2_ Cryostream system (Oxford Cryosystems, Oxford, UK) allowing a sample equilibration for at least 30 minutes before the individual measurements.

The sample was placed in an open borosilicate glass capillary of 0.5 mm diameter in the temperature range between 25 °C and 125 °C, showing to the formation of **Na[Au(N_3_)_4_]·H_2_O** and **Na[Au(N_3_)_4_] (Figure S6e)**. Presumably due to reaction kinetics, thermodynamic equilibrium was not achieved. Great care was taken to keep the temperature below the decomposition temperature to prevent mechanical damages of the diffractometer.

Other highly sophisticated investigation methods like neutron diffraction experiments were out of the question due to the highly explosive nature of some of the phases involved and the as small as possible amounts of sample required to operate safely.

**
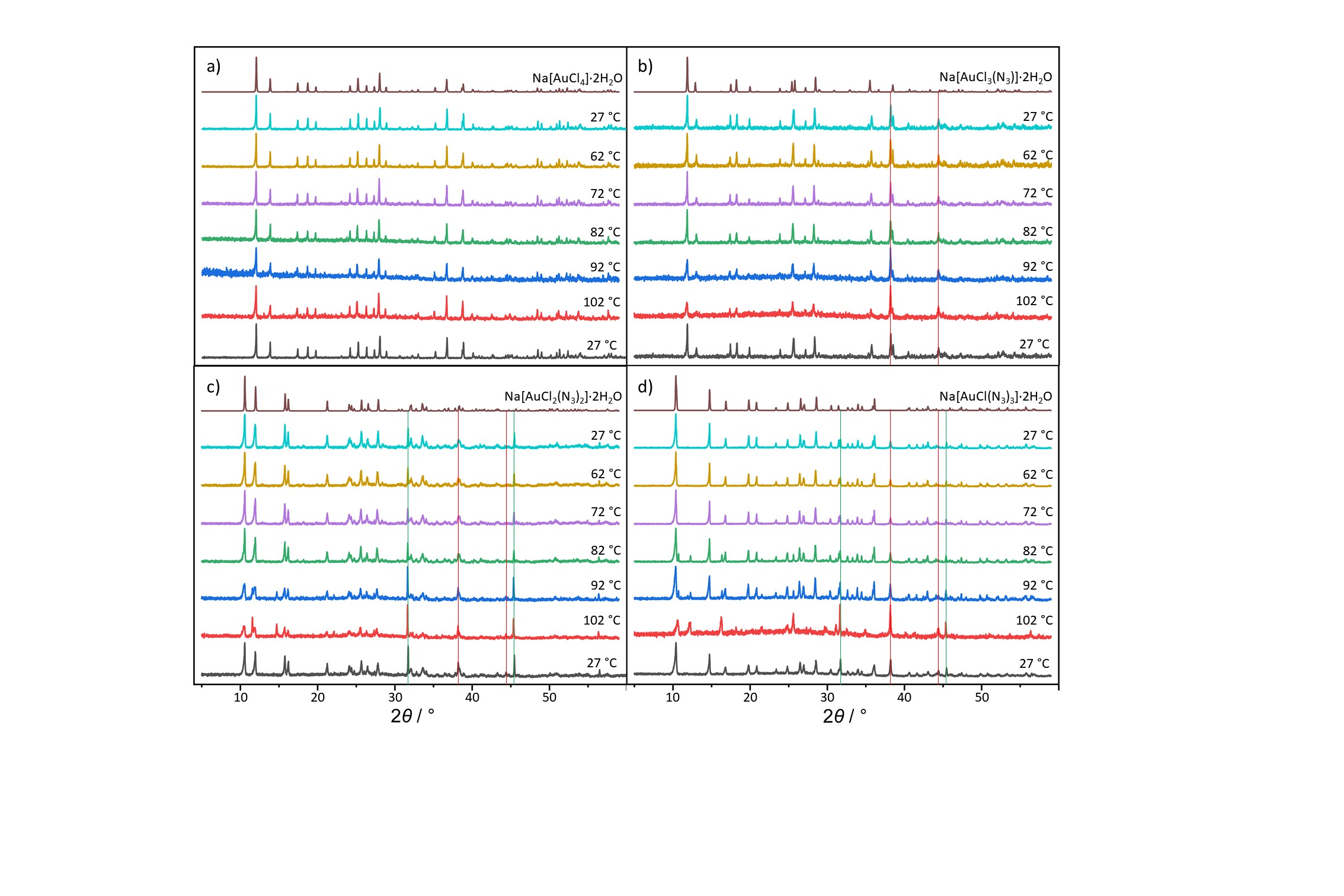
**

**Figure S6a-d**: High temperature powder diffraction measurement of a) Na[AuCl_4_]·2H_2_O, b) Na[AuCl_3_(N_3_)]·2H_2_O, c) Na[AuCl_2_(N_3_)_2_]·2H_2_O, and d) Na[AuCl(N_3_)_3_]·2H_2_O. Whereas in Na[AuCl_4_]·2H_2_O and Na[AuCl_3_(N_3_)]·2H_2_O no changes are evident, dehydration starts in Na[AuCl_2_(N_3_)_2_]·2H_2_O at 92 °C and in Na[AuCl_3_(N_3_)]·2H_2_O at 82 °C. Cooling to room temperature reestablishes the dihydrated phases, although some decomposition is evident from increased intensity of Au (red) and NaCl (green) peaks.

**
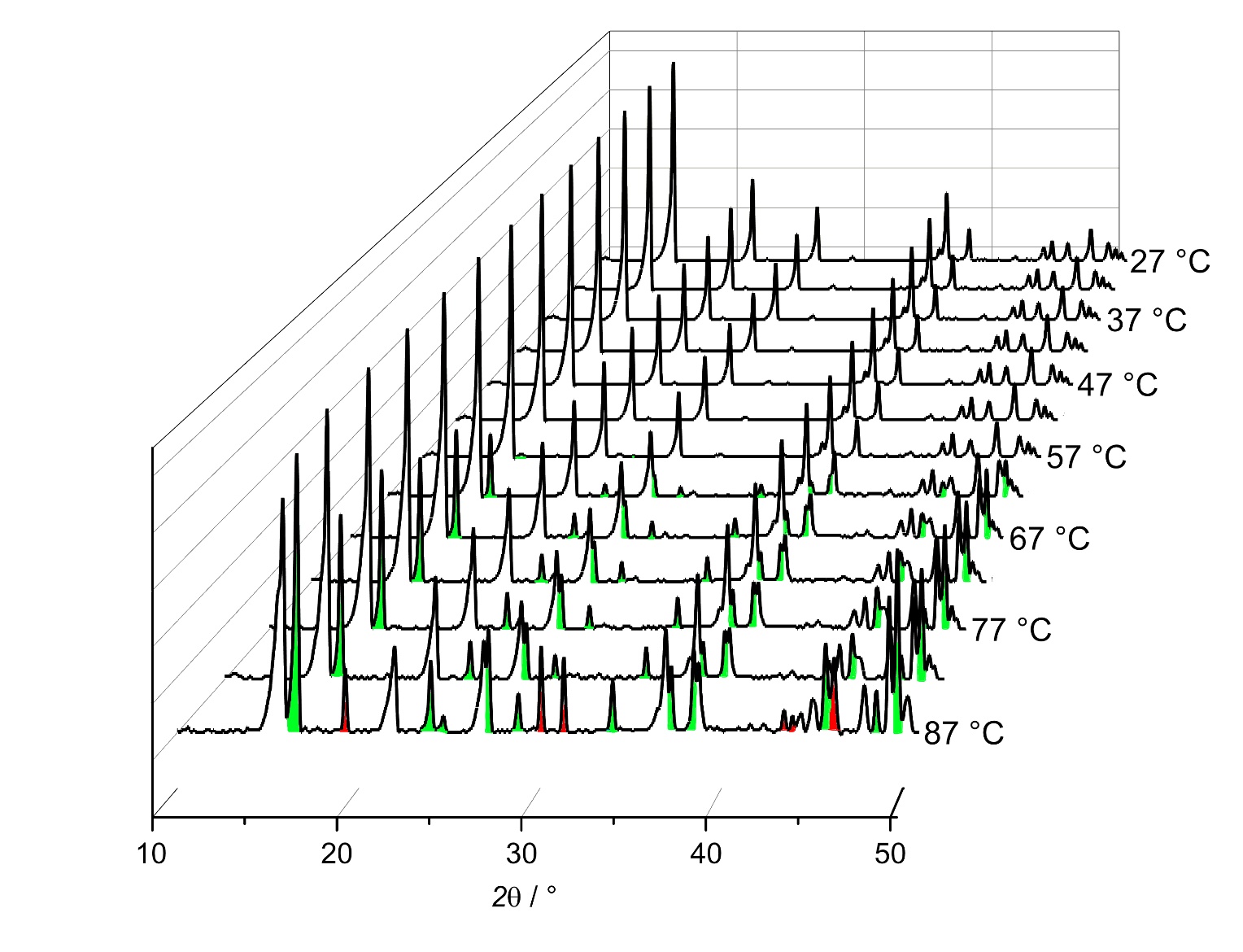
Figure S6e**: High temperature powder diffraction measurement of Na[Au(N_3_)_4_]·2H_2_O. The transformation to Na[Au(N_3_)_4_]·H_2_O (green) starts at 345 K and is not finished at the end of the measurement at 360 K, when Na[Au(N_3_)_4_] (red) already begins to appear.

## **Differential Scanning Calorimetry**

The thermal behavior of **Na[Au(N_3_)_4_]·2H_2_O** and AgN_3_ was characterized using a power-compensated DSC (DSC 8500, from Perkin Elmer). Samples of the compounds were measured under the following conditions: Sample mass: 1.0 mg, (AgN_3_ 2.5 mg) heating and cooling rate: 10 K/min, crucible: screwable high-pressure stainless-steel crucible. The measurements were carried out in a temperature range from 25 to 400 °C in a flowing argon atmosphere (Ar 99.999% 100 ml/min with additional drying and oxygen post-purification via Big Oxygen Trap from Trigon Technologies).

The temperature and heat flow were calibrated with indium and zinc. The evaluation was carried out using the sapphire method.

Reactions of **Na[AuCl_3_N_3_]·2H_2_O**, **Na[AuCl_2_(N_3_)_2_]·2H_2_O**, and **Na[AuCl(N_3_)_3_]·2H_2_O** with the crucible material may also occur in the high-pressure stainless steel DSC cells, as evidenced by the observable corrosion and rendering the measurements useless. The products obtained in this process cannot be clearly identified.

## **Thermal analysis**

The thermal decomposition behavior of **Na[AuCl_4_]·2H_2_O**, **Na[AuCl_3_N_3_]·2H_2_O**, **Na[AuCl_2_(N_3_)_2_]·2H_2_O**, and **Na[AuCl(N_3_)_3_]·2H_2_O** was also investigated by DTA/TG using an STA 409 (NETZSCH). The individual samples were measured under the following conditions: Atmosphere: static ambient air, Temperature range: 25 to 200 °C, Heating and cooling rate: 5 K/min, Sample mass: 4.1 to 6.7 mg, Crucible: corundum DTA/TG crucible with perforated lid, Thermocouple: type S (PtRh/Pt). Due to the small sample mass and the low mass loss, the signal-to-noise ratio of the thermogravimetric measurements is so poor that a comprehensive evaluation is not meaningful.

## **Vibrational spectroscopy**

FT-Raman spectra were obtained using a Renishaw inVia Raman microscope equipped with a 532 nm excitation laser source. The Raman spectra of selected crystals were recorded at room temperature in the range of 4000 to 50 cm^−1^ using either a Horiba LabRam HR Evolution spectrometer equipped with a He-Ne laser (λ = 633 nm) and a Synapse CCD detector or Bruker Senterra II micro-Raman spectrometer equipped with a diode laser (λ = 532 nm).

The investigation of spectral behavior of the [Au(N_3_)_4_]^–^ anions in the anhydrous state turned out to be a quite difficult issue since the dry substance may undergo a spontaneous decomposition which is accompanied by a violent explosion. The process is strongly enhanced when laser is used as a radiation source which is the case for Raman investigations. Therefore, the spectra of Na[Au(N_3_)_4_]·H_2_O and Na[Au(N_3_)_4_] were investigated only by IR spectroscopy.

Infrared spectra were recorded in Attenuated Total Reflectance (ATR) mode using either a Thermo Scientific iS50 FT-IR spectrometer with a single reflection diamond ATR module, a PerkinElmer UATR-Two FTIR spectrometer (spectral range 4000−450 cm−1) operated in air, or a similar instrument located in a glove box and operated under Argon.

Temperature-dependent IR measurements of **Na[Au(N_3_)_4_]·2H_2_O** were carried out by using a tungsten carbide mortar heated to 150 °C instead of the ATR pressing unit and recording IR spectra approximately every 3 minutes (Figure S9). In this way, it was possible to investigate the transition from **Na[Au(N_3_)_4_]·2H_2_O** to **Na[Au(N_3_)_4_]** and back *in situ* and obtain IR data of **Na[Au(N_3_)_4_]·H_2_O** that could be compared with spectra of the starting material as well as those of the anhydrous phase **Na[Au(N_3_)_4_]** recorded in the glove box.

## **DFT calculations**

Quantum chemical calculations were conducted using the ORCA (version 6.0) program package.^[7]^ Optimized molecular structures were obtained using DFT ^[8]^ with the B3LYP functional ^[9-12]^ in combination with the LANL2DZ basis set.^[13-16]^

Frequency calculations were performed for all optimized geometries, confirming their stability by the absence of imaginary frequencies. Cartesian force constants extracted from the ORCA Hessian file were converted to internal coordinate force constants using the FCT program.^[17-20]^ Visualization of molecular structures was carried out using the JMOL program.^[21]^ Optimized geometries of the complexes are presented in Figure 14, while calculated bond lengths and force constants are summarized in Table S9. For clarity, molecular structures in the text, Table S9, and Figure 14 are denoted as S followed by a number.

Details of the structure description

**Table S1.** Crystallographic data for Na[Au(Cl_4–_*_x_*(N_3_)*_x_*]·2H_2_O (*x* = 0, 1, 2, 3, 4).

| Composition | Na[AuCl_4_]·2H_2_O | Na[AuCl_3_(N_3_)]·2H_2_O | Na[AuCl_2_(N_3_)_2_]·2H_2_O | Na[AuCl(N_3_)_3_]·2H_2_O | Na[Au(N_3_)_4_]·2H_2_O |
| --- | --- | --- | --- | --- | --- |
| *x* | 0 | 0.89(2) | 2 | 3 | 4 |
| Refinement method |  |  | Single crystal  SHELXL-2018^[2-3]^ |  |  |
| Crystal system | orthorhombic | orthorhombic | triclinic | orthorhombic | monoclinic |
| Crystal color, shape | light yellow transparent prism-like crystals | orange-brown transparent prism-like crystals | orange transparent prism-like crystals | orange transparent prism-like crystals | orange transparent prism-like crystals |
| Space group | *Pnma* (No. 62) | *Pnma* (No. 62) | *P*$\bar{\text{1}}$ (No. 2) | *Pbcm* (No. 57) | *P*2_1_/*c* (No. 14) |
| *a* [Å]  *b* [Å]  *c* [Å] α [°]  β [°] γ [°] | 12.8305(5)  7.0639(3)  9.0047(4) | 13.7174(10) 6.9060(5)  8.8804(5) | 3.6821(12) 7.4598(19)  8.3583(19) 91.405(13) 90.401(17) 97.933(17) | 8.5264(17) 16.9020(30) 6.7071(14) | 3.5843(6) 13.037(2) 22.044(3)  92.518(9) |
| *V* [Å^3^] | 816.13(6) | 841.26(10) | 227.31(11) | 966.6(3) | 1029.1(3) |
| *Z* | 4 | 4 | 1 | 4 | 4 |
| *V*/*Z* [Å^3^] | 204.03 | 210.32 | 227.31 | 241.65 | 257.28 |
| Molar mass [g⋅mol^–1^] | 397.80 | 403.68 | 410.95 | 417.50 | 424.11 |
| δ*_x_* [g⋅cm^–3^] | 3.238 | 3.187 | 3.002 | 2.869 | 2.737 |
| Temperature *T* [K] |  |  | 293(2) |  |  |
| Diffractometer |  |  | Rigaku AFC7 |  |  |
| Detector |  |  | CCD Saturn 724+ |  |  |
| Radiation, λ [Å] |  |  | Mo *K*α, 0.71073 |  |  |
| Scan mode |  |  | ϕ |  |  |
| 2θ range [°] | 7.3–72.0 | 5.5–60.0 | 7.3–57.0 | 5.3–52.0 | 3.6–52.0 |
| *hkl* ranges | –21 ≤ h ≤ 18,  –11 ≤ k ≤ 9,  –14 ≤ l ≤ 9 | –19 ≤ h ≤ 17,  –9 ≤ k ≤ 4,  –11 ≤ l ≤ 12 | –4 ≤ h ≤ 2,  –9 ≤ k ≤ 9,  –11 ≤ l ≤ 11 | –10 ≤ h ≤ 10,  –20 ≤ k ≤ 19,  –8 ≤ l ≤ 6 | –4 ≤ h ≤ 3,  –16 ≤ k ≤ 14,  –27 ≤ l ≤ 26 |
| No. refl. | 13539 | 5379 | 2382 | 5118 | 5461 |
| No. refl. unique | 2050 | 1311 | 2382 | 1041 | 2010 |
| F(000) [*e*] | 712 | 726 | 186 | 760 | 776 |
| μ [mm^–1^] | 19.309 | 18.470 | 16.787 | 15.534 | 14.350 |
| *R*_int_ | 0.0307 | 0.0599 | * | 0.0412 | 0.0439 |
| Refined parameters | 54 | 68 | 65 | 88 | 157 |
| *R*1, *wR*2 (all data) | 0.035, 0.074 | 0.048, 0.083 | 0.052, 0.093 | 0.049, 0.095 | 0.056, 0.111 |
| GOOF | 1.121 | 1.009 | 1.049 | 1.167 | 1.041 |
| Highest electron diff. peak | 3.249 Å^–3^ | 3.853 Å^–3^ | 1.792 Å^–3^ | 3.236 | 2.635 |
| Deepest electron diff. hole | –2.948 Å^–3^ | –2.066 Å^–3^ | –1.142 Å^–3^ | –1.771 | –2.102 |
| * Not averaged due to twin; twin matrix (100,-1/2-10,00-1), twin component ratio 0.768(2)/0.232. | | | | | |

**Table S2a.** Atomic coordinates and isotropic displacement parameters [Å^2^] for Na[AuCl_4_]·2H_2_O. Standard deviations are given in parentheses.

| Atom | Site | *x / a* | *y / b* | *z / c* | *U*_eq/iso_ / Å^2^ |
| --- | --- | --- | --- | --- | --- |
| Au1 | 4*c* | 0.43895(2) | ¼ | 0.51801(2) | 0.02967(7) |
| Cl1 | 4*c* | 0.29441(8) | ¼ | 0.66735(13) | 0.0407(2) |
| Cl2 | 4*c* | 0.58424(10) | ¼ | 0.37221(16) | 0.0464(3) |
| Cl3 | 4*c* | 0.54342(10) | ¼ | 0.72272(18) | 0.0527(3) |
| Cl4 | 4*c* | 0.33340(10) | ¼ | 0.31259(14) | 0.0492(3) |
| Na1 | 4*c* | 0.48835(19) | ¼ | 0.0671(3) | 0.0530(5) |
| O1 | 8*d* | 0.1199(3) | 0.5006(5) | 0.4676(3) | 0.0463(6) |
| H1 | 8*d* | 0.158(4) | 0.528(7) | 0.382(3) | 0.069* |
| H2 | 8*d* | 0.177(3) | 0.506(11) | 0.533(5) | 0.069* |
| \| * U_eq_(H*x*) = 1.5 U_eq_(O1) \| \| --- \| | | | | | |

**Table S3a.** Anisotropic displacement parameters [Å^2^] for Na[AuCl_4_]·2H_2_O. Standard deviations are given in parentheses.

| Atom | *U*_11_ / Å^2^ | *U*_22_ / Å^2^ | *U*_33_ / Å^2^ | *U*_23_ / Å^2^ | *U*_13_ / Å^2^ | *U*_12_ / Å^2^ |
| --- | --- | --- | --- | --- | --- | --- |
| Au1 | 0.02915(9) | 0.02871(9) | 0.03115(10) | 0 | 0.00022(5) | 0 |
| Cl1 | 0.0312(4) | 0.0569(6) | 0.0340(5) | 0 | 0.0018(4) | 0 |
| Cl2 | 0.0386(5) | 0.0545(7) | 0.0462(7) | 0 | 0.0118(5) | 0 |
| Cl3 | 0.0392(5) | 0.0784(9) | 0.0405(7) | 0 | -0.0101(5) | 0 |
| Cl4 | 0.0415(6) | 0.0730(8) | 0.0331(5) | 0 | -0.0042(4) | 0 |
| Na1 | 0.0520(12) | 0.0493(11) | 0.0578(14) | 0 | -0.0016(11) | 0 |
| O1 | 0.0452(13) | 0.0499(14) | 0.0437(14) | 0.0063(12) | 0.0036(11) | 0.0059(11) |

**Table S4a.** Selected interatomic distances [Å] and bond angles [°] in Na[AuCl_4_]·2H_2_O with their multiplicity (*n*). Standard deviations are given in parentheses.

| Atoms |  | *d* [Å] | Atoms |  | *d* [Å] |
| --- | --- | --- | --- | --- | --- |
| Au1− | Au1 2x | 3.8773(2) | Na1 | Na1 2x | 3.7450(18) |
|  |  |  |  |  |  |
| Au1− | Cl3 | 2.2791(14) | Na1− | O1 2x | 2.415(4) |
|  | Cl2 | 2.2800(12) |  | O1 2x | 2.466(4) |
|  | Cl1 | 2.2908(11) |  | Cl4 | 2.973(3) |
|  | Cl4 | 2.2925(12) |  | Cl2 | 3.010(3) |
|  |  |  |  | Cl3 | 3.181(3) |
| O1− | H1 | 0.935(16) |  |  |  |
|  | H2 | 0.942(16) |  |  |  |
|  |  | ° |  |  | ° |
| ∠ (Au1–Au1– Au1) | | 131.263(1) | ∠ (O1–Na1–O1) | | 93.64(19) |
|  | |  | ∠ (O1–Na1–O1) 2x | | 79.80(12) |
| ∠ (Na1– Na1– Na1) | | 141.173(1) | ∠ (O1–Na1–O1) 2x | | 150.46(12) |
|  | |  | ∠ (O1–Na1–Cl4) 2x | | 83.76(10) |
| ∠ (Cl3–Au1– Cl2) | | 89.14(5) | ∠ (O1–Na1–Cl2) 2x | | 125.00(10) |
| ∠ (Cl3–Au1– Cl1) | | 90.08(4) | ∠ (O1–Na1–Cl3) 2x | | 76.47(10) |
| ∠ (Cl3–Au1– Cl4) | | 179.81(5) | ∠ (O1–Na1–O1) | | 91.78(17) |
| ∠ (Cl2–Au1– Cl1) | | 179.21(5) | ∠ (O1–Na1–Cl4) 2x | | 123.50(9) |
| ∠ (Cl2–Au1– Cl4) | | 91.05(5) | ∠ (O1–Na1–Cl2) 2x | | 80.56(9) |
| ∠ Cl1–Au1– Cl4) | | 89.74(4) | ∠ (O1–Na1–Cl3) 2x | | 74.00(8) |
|  | |  | ∠ (Cl4–Na1–Cl2) | | 66.09(7) |
| ∠ (Na1–O1–Na1) | | 100.20(12) | ∠ (Cl4–Na1–Cl3) | | 150.87(9) |
| ∠ (H1–O1–H2) | | 96(5) | ∠ (Cl2–Na1–Cl3) | | 143.04(9) |

**Table S2b.** Atomic coordinates and isotropic displacement parameters [Å^2^] for Na[AuCl_3_N_3_]·2H_2_O. Standard deviations are given in parentheses.

| Atom | Site | Occ. | *x / a* | *y / b* | *z / c* | *U*_eq/iso_ / Å^2^ | |
| --- | --- | --- | --- | --- | --- | --- | --- |
| Au1 | 4*c* | 1 | 0.42829(2) | ¼ | 0.52076(4) | 0.03834(13) | |
| Cl1 | 4*c* | 1 | 0.29438(17) | ¼ | 0.6756(2) | 0.0526(5) | |
| Cl2 | 4*c* | 0.109(19) | 0.573(2) | ¼ | 0.377(4) | 0.109(19) | |
| N2 | 4*c* | 0.891(19) | 0.5384(11) | ¼ | 0.3669(14) | 0.054(3) | |
| N2A | 4*c* | 0.891(19) | 0.6207(10) | ¼ | 0.4258(13) | 0.049(3) | |
| N2B | 4*c* | 0.891(19) | 0.7034(8) | ¼ | 0.4754(11) | 0.061(3) | |
| Cl3 | 4*c* | 1 | 0.52941(18) | ¼ | 0.7230(2) | 0.0586(6) | |
| Cl4 | 4*c* | 1 | 0.3288(2) | ¼ | 0.3158(3) | 0.0635(7) | |
| Na1 | 4*c* | 1 | 0.4883(3) | ¼ | 0.0859(5) | 0.0623(10) | |
| O1 | 8*d* | 1 | 0.1125(4) | 0.4942(9) | 0.4710(5) | 0.0565(12) | |
| H1 | 8*d* | 1 | 0.149(5) | 0.520(11) | 0.383(5) | 0.085* | |
| H2 | 8*d* | 1 | 0.157(5) | 0.438(11) | 0.541(7) | 0.085* | |
| \| * U_eq_(H*x*) = 1.5 U_eq_(O1) \| \| --- \| | | | | | | |  |

**Table S3b.** Anisotropic displacement parameters [Å^2^] for Na[AuCl_3_N_3_]·2H_2_O. Standard deviations are given in parentheses.

| Atom | *U*_11_ / Å^2^ | *U*_22_ / Å^2^ | *U*_33_ / Å^2^ | *U*_23_ / Å^2^ | *U*_13_ / Å^2^ | *U*_12_ / Å^2^ |
| --- | --- | --- | --- | --- | --- | --- |
| Au1 | 0.0400(2) | 0.0422(2) | 0.03280(19) | 0 | 0.00042(12) | 0 |
| Cl1 | 0.0451(13) | 0.0753(15) | 0.0375(10) | 0 | 0.0047(9) | 0 |
| N2 | 0.035(7) | 0.071(7) | 0.056(6) | 0 | 0.005(6) | 0 |
| N2A | 0.051(7) | 0.053(5) | 0.042(5) | 0 | 0.015(5) | 0 |
| N2B | 0.041(6) | 0.063(6) | 0.080(7) | 0 | 0.025(5) | 0 |
| Cl3 | 0.0451(13) | 0.0922(18) | 0.0385(11) | 0 | -0.0067(10) | 0 |
| Cl4 | 0.0563(15) | 0.0980(18) | 0.0361(11) | 0 | -0.0060(10) | 0 |
| Na1 | 0.054(3) | 0.073(3) | 0.060(2) | 0 | -0.0028(18) | 0 |
| O1 | 0.053(3) | 0.065(3) | 0.051(3) | 0.010(2) | 0.000(2) | -0.005(3) |

**Table S4b.** Selected interatomic distances [Å] and bond angles [°] in Na[AuCl_3_N_3_]·2H_2_O with their multiplicity (*n*). Standard deviations are given in parentheses.

| Atoms |  | *d* [Å] | Atoms |  | *d* [Å] |
| --- | --- | --- | --- | --- | --- |
| Au1− | Au1 2x | 3.991(3) | Na1 | Na1 2x | 3.789(4) |
|  |  |  |  |  |  |
| Au1− | Cl3 | 2.269(2) | Na1− | O1 2x | 2.451(6) |
|  | N2 | 2.037(12) |  | O1 2x | 2.464(7) |
|  | Cl2 | 2.36(3) |  | Cl4 | 2.992(5) |
|  | Cl1 | 2.295(2) |  | N2 | 2.589(14) |
|  | Cl14 | 2.275(2) |  | Cl2 | 2.84(3) |
|  |  |  |  | Cl3 | 3.272(5) |
| N2– | N2A | 1.24(3) |  |  |  |
| N2A– | N2B | 1.216(19) | O1– | H1 | 0.94(6) |
|  |  |  |  | H2 | 0.95(7) |
|  |  | ° |  |  | ° |
| ∠ (Au1–Au1– Au1) | | 119.80(1) | ∠ (O1–Na1–O1) | | 87.0(3) |
|  | |  | ∠ (O1–Na1–O1) 2x | | 79.1(2) |
| ∠ (Na1– Na1– Na1) | | 131.40(1) | ∠ (O1–Na1–O1) 2x | | 142.96(19) |
|  | |  | ∠ (O1–Na1–Cl4) 2x | | 130.47(15) |
| ∠ (Cl3–Au1–N2) | | 94.4(4) | ∠ (O1–Na1–N2) 2x | | 90.8(3) |
| ∠ (Cl3–Au1–Cl1) | | 90.86(8) | ∠ (O1–Na1–Cl3) 2x | | 71.15(14) |
| ∠ (Cl3–Au1–Cl4) | | 179.18(9) | ∠ (O1–Na1–O1) | | 91.6(3) |
| ∠ (N2–Au1–Cl1) | | 174.7(4) | ∠ (O1–Na1–Cl4) 2x | | 82.67(16) |
| ∠ (N2–Au1–Cl4) | | 84.7(4) | ∠ (O1–Na1–N2) 2x | | 123.3(2) |
| ∠ Cl1–Au1–Cl4) | | 89.96(9) | ∠ (O1–Na1–Cl3) 2x | | 71.88(14) |
| ∠ (Cl3–Au1–Cl2) | | 85.0(8) | ∠ (Cl4–Na1–N2) | | 62.4(3) |
| ∠ (Cl2–Au1–Cl1) | | 175.9(8) | ∠ (Cl4–Na1–Cl3) | | 142.97(16) |
| ∠ (Cl2–Au1–Cl4) | | 94.1(8) | ∠ (N2–Na1–Cl3) | | 154.6(4) |
| ∠ (N2–Au1–Cl2) | | 9.4(10) |  | |  |
|  | |  | ∠ (Na1–O1–Na1) | | 100.9(2) |
| ∠ (Au1–N2–N2A) | | 113.0(8) | ∠ (H1–O1–H2) | | 106(5) |
| ∠ (N2–N2A–N2B) | | 176.4(10) |  | |  |

**Table S2c.** Atomic coordinates and isotropic displacement parameters [Å^2^] for Na[AuCl_2_(N_3_)_2_]·2H_2_O. Standard deviations are given in parentheses.

| Atom | Site | *x / a* | *y / b* | *z / c* | *U*_eq/iso_ / Å^2^ |  |
| --- | --- | --- | --- | --- | --- | --- |
| Au1 | 1*a* | 0 | 0 | 0 | .0402(2) |  |
| N1 | 2*i* | 0.063(3) | 0.9479(12) | 0.2357(11) | 0.051(3) |  |
| N1A | 2*i* | 0.226(3) | 0.0678(13) | 0.3192(11) | 0.053(3) |  |
| N1B | 2*i* | 0.370(4) | 0.1705(14) | 0.4115(13) | 0.072(4) |  |
| Cl1 | 2*i* | 0.3094(9) | 0.2867(3) | 0.0295(3) | 0.0475(6) |  |
| Na1 | 1*h* | ½ | ½ | ½ | 0.0534(18) |  |
| O1 | 2*i* | 0.013(2) | 0.5613(10) | 0.3216(9) | 0.050(2) |  |
| H1 | 2*i* | 0.00(3) | 0.479(14) | 0.232(10) | 0.075* |  |
| H2 | 2*i* | 0.05(3) | 0.677(8) | 0.272(14) | 0.075* |  |
| \| * U_eq_(H*x*) = 1.5 U_eq_(O1) \| \| --- \| | | | | | | |

**Table S3c.** Anisotropic displacement parameters [Å^2^] for Na[AuCl_2_(N_3_)_2_]·2H_2_O. Standard deviations are given in parentheses.

| Atom | *U*_11_ / Å^2^ | *U*_22_ / Å^2^ | *U*_33_ / Å^2^ | *U*_23_ / Å^2^ | *U*_13_ / Å^2^ | *U*_12_ / Å^2^ |
| --- | --- | --- | --- | --- | --- | --- |
| Au1 | 0.0485(4) | 0.0309(3) | 0.0407(4) | 0.0010(2) | 0.0010(3) | 0.0044(3) |
| N1 | 0.077(8) | 0.035(5) | 0.037(5) | 0.000(4) | -0.006(5) | -0.001(5) |
| N1A | 0.075(8) | 0.045(6) | 0.043(5) | 0.003(5) | -0.006(6) | 0.023(6) |
| N1B | 0.112(12) | 0.045(6) | 0.057(7) | -0.003(5) | -0.023(6) | -0.001(6) |
| Cl1 | 0.0566(18) | 0.0320(12) | 0.0514(15) | 0.0001(11) | -0.0037(15) | -0.0027(13) |
| Na1 | 0.064(5) | 0.043(3) | 0.051(4) | 0.002(3) | -0.005(3) | 0.001(3) |
| O1 | 0.062(6) | 0.045(5) | 0.044(5) | 0.006(4) | 0.001(4) | 0.010(4) |

**Table S4c.** Selected interatomic distances [Å] and bond angles [°] in Na[AuCl_2_(N_3_)_2_]·2H_2_O with their multiplicity (*n*). Standard deviations are given in parentheses.

| Atoms |  | *d* [Å] | Atoms |  | *d* [Å] |
| --- | --- | --- | --- | --- | --- |
| Au1− | Au1 2x | 3.6821(12) | Na1 | Na1 2x | 3.6821(12) |
|  |  |  |  |  |  |
| Au1− | N1 2x | 2.035(9) | Na1− | O1 2x | 2.414(8) |
|  | Cl1 2x | 2.287(3) |  | O1 2x | 2.424(8) |
| N1– | N1A | 1.211(13) |  | N1B 2x | 2.529(10) |
| N1A– | N1B | 1.147(13) | O1− | H1 | 0.95(2) |
|  |  |  |  | H2 | 0.95(2) |
|  |  | ° |  |  | ° |
| ∠ (Au1–Au1– Au1) | | 180 | ∠ (O1–Na1–O1) 2x | | 180 |
|  | |  | ∠ (O1–Na1–O1) 2x | | 99.1(3) |
| ∠ (Na1– Na1– Na1) | | 180 | ∠ (O1–Na1–O1) 2x | | 80.9(3) |
|  | |  | ∠ (O1–Na1–N1B) 2x | | 86.9(4) |
| ∠ (N1–Au1– N1) | | 180 | ∠ (O1–Na1–N1B) 2x | | 88.5(3) |
| ∠ (N1–Au1– Cl1) 2x | | 87.5(3) | ∠ (O1–Na1–N1B) 2x | | 91.5(3) |
| ∠ (N1–Au1– Cl1) 2x | | 92.5(3) | ∠ (O1–Na1–N1B) 2x | | 93.1(4) |
| ∠ (Cl1–Au1– Cl1) | | 180 | ∠ (N1B–Na1–N1B) | | 180 |
|  | |  |  | |  |
| ∠ (Au1–N1–N1A) | | 117.2(7) | ∠ (Na1–O1–Na1) | | 99.1(3) |
| ∠ (N1–N1A–N1B) | | 173.0(12) | ∠ (H1–O1–H2) | | 103(10) |

**Table S2d.** Atomic coordinates and isotropic displacement parameters [Å^2^] for Na[AuCl(N_3_)_3_]·2H_2_O. Standard deviations are given in parentheses.

| Atom | Site | *x / a* | *y / b* | *z / c* | *U*_eq_ / Å^2^ |  |
| --- | --- | --- | --- | --- | --- | --- |
| Au1 | 4*d* | 0.49557(4) | 0.20008(2) | ¼ | 0.0362(2) |  |
| Cl1 | 4*d* | 0.5187(3) | 0.0643(2) | ¼ | 0.0519(9) |  |
| N1 | 4*d* | 0.2572(13) | 0.1872(6) | ¼ | 0.051(3) |  |
| N1A | 4*d* | 0.1799(12) | 0.2458(7) | ¼ | 0.054(3) |  |
| N1B | 4*d* | 0.0941(15) | 0.2995(7) | ¼ | 0.077(4) |  |
| N2 | 4*d* | 0.4652(12) | 0.3218(6) | ¼ | 0.045(3) |  |
| N2A | 4*d* | 0.5894(13) | 0.3553(6) | ¼ | 0.047(3) |  |
| N2B | 4*d* | 0.7050(13) | 0.3924(7) | ¼ | 0.076(4) |  |
| N3 | 4*d* | 0.7310(14) | 0.2163(7) | ¼ | 0.080(5) |  |
| N3A | 4*d* | 0.8132(13) | 0.1650(7) | ¼ | 0.060(3) |  |
| N3B | 4*d* | 0.9088(18) | 0.1163(9) | ¼ | 0.138(9) |  |
| Na1 | 4*d* | 0.0237(6) | –0.0388(3) | ¼ | 0.0522(14) |  |
| O1 | 8*e* | 0.1711(7) | 0.0383(4) | –0.0004(10) | 0.0502(15) |  |
| H1 | 8*e* | 0.270(6) | 0.016(5) | –0.038(14) | 0.075* |  |
| H2 | 8*e* | 0.199(11) | 0.083(4) | 0.079(12) | 0.075* |  |
| \| * U_eq_(H*x*) = 1.5 U_eq_(O1) \| \| --- \| | | | | | | |

**Table S3d.** Anisotropic displacement parameters [Å^2^] for Na[AuCl(N_3_)_3_]·2H_2_O. Standard deviations are given in parentheses.

| Atom | *U*_11_ / Å^2^ | *U*_22_ / Å^2^ | *U*_33_ / Å^2^ | *U*_23_ / Å^2^ | *U*_13_ / Å^2^ | *U*_12_ / Å^2^ |
| --- | --- | --- | --- | --- | --- | --- |
| Au1 | 0.0345(3) | 0.0327(3) | 0.0413(3) | 0 | 0 | –0.00620(18) |
| Cl1 | 0.0427(17) | 0.0383(15) | 0.075(3) | 0 | 0 | –0.0034(13) |
| Na1 | 0.049(3) | 0.065(4) | 0.042(3) | 0 | 0 | 0.002(2) |
| N1 | 0.038(6) | 0.034(6) | 0.081(9) | 0 | 0 | 0.002(4) |
| N1A | 0.040(6) | 0.050(7) | 0.071(8) | 0 | 0 | –0.018(6) |
| N1B | 0.052(7) | 0.042(7) | 0.136(14) | 0 | 0 | 0.005(6) |
| N2 | 0.043(6) | 0.035(5) | 0.057(7) | 0 | 0 | –0.007(5) |
| N2A | 0.058(7) | 0.028(5) | 0.054(7) | 0 | 0 | 0.011(5) |
| N2B | 0.049(7) | 0.043(7) | 0.135(12) | 0 | 0 | –0.023(6) |
| N3 | 0.039(6) | 0.043(7) | 0.159(15) | 0 | 0 | –0.015(6) |
| N3A | 0.035(6) | 0.034(6) | 0.110(10) | 0 | 0 | –0.013(5) |
| N3B | 0.050(9) | 0.065(10) | 0.30(3) | 0 | 0 | –0.001(8) |
| O1 | 0.042(3) | 0.046(4) | 0.062(4) | 0.000(3) | –0.006(3) | –0.003(3) |

**Table S4d.** Selected interatomic distances [Å] and bond angles [°] in Na[AuCl(N_3_)_3_]·2H_2_O with their multiplicity (*n*). Standard deviations are given in parentheses.

| Atoms |  | *d* [Å] | Atoms |  | *d* [Å] |
| --- | --- | --- | --- | --- | --- |
| Au1− | Au1 2x | 3.7541(7) | Na1 | Na1 2x | 3.624(4) |
|  |  |  |  |  |  |
| Au1− | N3 | 2.026(12) | Na1− | O1 2x | 2.358(7) |
|  | N1 | 2.044(11) |  | O1 2x | 2.470(8) |
|  | N2 | 2.074(11) |  | N2B | 2.589(11) |
|  | Cl1 | 2.304(3) |  | N3B | 2.799(17) |
|  |  |  |  | N1B | 2.911(13) |
| N1– | N1A | 1.190(15) |  |  |  |
| N1A– | N1B | 1.166(15) | O1– | H1 | 0.955(17) |
| N2– | N2A | 1.201(14) |  | H2 | 0.953(17) |
| N2A– | N2B | 1.168(14) |  |  |  |
| N3– | N3A | 1.115(15) |  |  |  |
| N3A– | N3B | 1.159(17) |  |  |  |
|  |  | ° |  |  | ° |
| ∠ (Au1–Au1–Au1) | | 126.577(1) | ∠ (Na1–Na1–Na1) | | 135.4(3) |
|  | |  |  | |  |
| ∠ (N3–Au1–N1) | | 178.4(4) | ∠ (O1–Na1–O1) | | 90.4(4) |
| ∠ (N3–Au1–N2) | | 89.4(4) | ∠ (O1–Na1–O1) 2x | | 82.7(2) |
| ∠ (N1–Au1–N2) | | 89.0(4) | ∠ (O1–Na1–O1) 2x | | 147.0(3) |
| ∠ (N3–Au1–Cl1) | | 92.9(4) | ∠ (O1–Na1–O1) | | 85.7(4) |
| ∠ (N1–Au1–Cl1) | | 88.8(3) | ∠ (O1–Na1–N2B) 2x | | 77.4(3) |
| ∠ (N2–Au1–Cl1) | | 177.7(3) | ∠ (O1–Na1–N2B) 2x | | 129.1(2) |
|  | |  | ∠ (O1–Na1–N3B) 2x | | 71.5(3) |
| ∠ (Au1–N1–N1A) | | 117.5(9) | ∠ (O1–Na1–N3B) 2x | | 75.5(3) |
| ∠ (N1–N1A–N1B) | | 174.7(13) | ∠ (O1–Na1–N1B) 2x | | 76.1(3) |
| ∠ (Au1–N2–N2A) | | 110.9(9) | ∠ (O1–Na1–N1B) 2x | | 132.2(2) |
| ∠ (N2–N2A–N2B) | | 175.6(13) | ∠ (N1B–Na1–N2B) | | 83.5(4) |
| ∠ (Au1–N3–N3A) | | 121.2(10) | ∠ (N1B–Na1–N3B) | | 139.3(5) |
| ∠ (N3–N3A–N3B) | | 174.2(15) | ∠ (N2B–Na1–N3B) | | 137.2(5) |
|  | |  |  | |  |
| ∠ (Na1–O1– Na1) | | 97.3(2) | ∠ (H1–O1–H2) | | 104(7) |

**Table S2e.** Atomic coordinates and isotropic displacement parameters [Å^2^] for Na[Au(N_3_)_4_]·2H_2_O. Standard deviations are given in parentheses.

| Atom | Site | *x / a* | *y / b* | *z / c* | *U*_eq/iso_ / Å^2^ |  |
| --- | --- | --- | --- | --- | --- | --- |
| Au1 | 4*e* | 0.40378(10) | 0.23198(3) | 0.38127(2) | 0.03435(18) |  |
| Na1 | 4*e* | 0.0552(12) | -0.1883(3) | 0.37100(18) | 0.0479(10) |  |
| N1 | 4*e* | 0.161(3) | 0.3469(7) | 0.3336(5) | 0.054(2) |  |
| N1A | 4*e* | 0.333(3) | 0.4241(7) | 0.3226(5) | 0.051(2) |  |
| N1B | 4*e* | 0.465(4) | 0.4993(10) | 0.3106(7) | 0.105(5) |  |
| N2 | 4*e* | 0.270(3) | 0.1348(7) | 0.3106(4) | 0.048(2) |  |
| N2A | 4*e* | 0.128(2) | 0.1757(7) | 0.2652(4) | 0.045(2) |  |
| N2B | 4*e* | -0.003(3) | 0.2081(8) | 0.2214(5) | 0.059(3) |  |
| N3 | 4*e* | 0.596(3) | 0.1032(7) | 0.4245(4) | 0.047(2) |  |
| N3A | 4*e* | 0.744(2) | 0.1130(6) | 0.4755(4) | 0.040(2) |  |
| N3B | 4*e* | 0.881(3) | 0.1172(7) | 0.5220(4) | 0.056(2) |  |
| N4 | 4*e* | 0.550(2) | 0.3156(6) | 0.4565(4) | 0.044(2) |  |
| N4A | 4*e* | 0.660(3) | 0.4019(7) | 0.4467(4) | 0.046(2) |  |
| N4B | 4*e* | 0.781(3) | 0.4827(8) | 0.4418(5) | 0.065(3) |  |
| O1 | 4*e* | 0.549(2) | -0.0740(6) | 0.3451(4) | 0.054(2) |  |
| O2 | 4*e* | -0.437(2) | -0.2932(6) | 0.4091(4) | 0.0523(19) |  |
| H11 | 4*e* | 0.73(3) | -0.037(9) | 0.370(5) | 0.079* |  |
| H21 | 4*e* | -0.40(3) | -0.349(6) | 0.382(4) | 0.079* |  |
| H12 | 4*e* | 0.50(3) | -0.079(10) | 0.3023(12) | 0.079* |  |
| H22 | 4*e* | -0.52(3) | -0.299(10) | 0.450(2) | 0.079* |  |
| \| * U_eq_(H*x*) = 1.5 U_eq_(O*x*) \| \| --- \| | | | | | | |

**Table S3e.** Anisotropic displacement parameters [Å^2^] for Na[Au(N_3_)_4_]·2H_2_O. Standard deviations are given in parentheses.

| Atom | *U*_11_ / Å^2^ | *U*_22_ / Å^2^ | *U*_33_ / Å^2^ | *U*_23_ / Å^2^ | *U*_13_ / Å^2^ | *U*_12_ / Å^2^ |
| --- | --- | --- | --- | --- | --- | --- |
| Au1 | 0.0388(3) | 0.0308(3) | 0.0337(3) | 0.00204(14) | 0.00358(17) | 0.00056(15) |
| Na1 | 0.051(2) | 0.045(3) | 0.048(3) | -0.003(2) | 0.0056(19) | 0.001(2) |
| N1 | 0.050(5) | 0.037(6) | 0.074(7) | 0.002(5) | -0.003(5) | 0.010(5) |
| N1A | 0.045(5) | 0.041(6) | 0.067(7) | 0.019(5) | -0.001(5) | 0.013(5) |
| N1B | 0.091(10) | 0.073(9) | 0.152(14) | 0.076(9) | 0.009(9) | 0.009(8) |
| N2 | 0.067(6) | 0.035(5) | 0.040(5) | -0.009(4) | -0.001(5) | 0.004(4) |
| N2A | 0.061(6) | 0.044(6) | 0.031(5) | 0.001(4) | -0.003(4) | -0.002(4) |
| N2B | 0.072(7) | 0.055(6) | 0.049(6) | 0.003(5) | -0.010(5) | -0.005(5) |
| N3 | 0.058(6) | 0.032(5) | 0.049(6) | 0.007(4) | -0.002(4) | 0.002(4) |
| N3A | 0.045(5) | 0.032(5) | 0.044(5) | 0.002(4) | 0.004(4) | 0.003(4) |
| N3B | 0.076(7) | 0.048(6) | 0.045(6) | -0.002(5) | 0.000(5) | 0.005(5) |
| N4 | 0.060(6) | 0.028(5) | 0.044(5) | 0.001(4) | 0.002(4) | -0.002(4) |
| N4A | 0.059(6) | 0.041(6) | 0.038(5) | -0.004(4) | -0.003(4) | 0.001(5) |
| N4B | 0.092(8) | 0.043(6) | 0.057(7) | -0.005(5) | -0.002(6) | -0.010(6) |
| O1 | 0.069(6) | 0.037(5) | 0.055(5) | 0.002(4) | 0.000(4) | 0.000(4) |
| O2 | 0.062(5) | 0.043(5) | 0.054(5) | 0.003(4) | 0.013(4) | 0.003(4) |

**Table S4e.** Selected interatomic distances [Å] and bond angles [°] in Na[Au(N_3_)_4_]·2H_2_O with their multiplicity (*n*). Standard deviations are given in parentheses.

| Atoms |  | *d* [Å] | Atoms |  | *d* [Å] |
| --- | --- | --- | --- | --- | --- |
| Au1− | Au1 2x | 3.5843(6) | Na1 | Na1 2x | 3.5843(6) |
|  |  |  |  |  |  |
| Au1− | N1 | 2.006(9) | Na1− | O1 | 2.398(9) |
|  | N4 | 2.035(8) |  | O2 | 2.398(9) |
|  | N3 | 2.035(9) |  | O1 | 2.401(9) |
|  | N2 | 2.049(9) |  | O2 | 2.412(9) |
|  |  |  |  | N2B | 2.445(11) |
| N1– | N1A | 1.211(13) |  | N3B | 2.536(11) |
| N1A– | N1B | 1.125(14) |  |  |  |
| N2– | N2A | 1.224(12) | O1− | H11 | 0.956(15) |
| N2A– | N2B | 1.137(12) |  | H12 | 0.957(15) |
| N3– | N3A | 1.228(12) | O2− | H12 | 0.955(15) |
| N3A– | N3B | 1.117(12) |  | H22 | 0.955(15) |
| N4– | N4A | 1.214(12) |  |  |  |
| N4A– | N4B | 1.147(13) |  |  |  |
|  |  | ° |  |  | ° |
| ∠ (Au1–Au1– Au1) | | 180 | ∠ (Na1– Na1– Na1) | | 180 |
|  | |  |  | |  |
| ∠ (N1–Au1–N4) | | 96.7(4) | ∠ (O1–Na1–O1) | | 96.7(3) |
| ∠ (N1–Au1–N3) | | 172.3(4) | ∠ (O1–Na1–O2) | | 172.9(3) |
| ∠ (N3–Au1–N4) | | 89.4(3) | ∠ (O1–Na1–O2) | | 83.2(3) |
| ∠ (N1–Au1–N2) | | 89.0(4) | ∠ (O1–Na1–O2) | | 82.9(3) |
| ∠ (N2–Au1–N4) | | 174.2(3) | ∠ (O1–Na1–O2) | | 172.9(3) |
| ∠ (N2–Au1–N3) | | 84.8(4) | ∠ (O2–Na1–O2) | | 96.3(3) |
|  | |  | ∠ (O1–Na1–N2B) | | 96.7(3) |
| ∠ (Au1–N1–N1A) | | 120.9(8) | ∠ (O1–Na1–N2B) | | 100.1(4) |
| ∠ (N1–N1A–N1B) | | 174.2(12) | ∠ (O1–Na1–N3B) | | 91.8(3) |
| ∠ (Au1–N2–N2A) | | 115.3(7) | ∠ (O1–Na1–N3B) | | 87.5(3) |
| ∠ (N2–N2A–N2B) | | 175.9(11) | ∠ (O2–Na1–N2B) | | 90.3(3) |
| ∠ (Au1–N3–N3A) | | 117.8(7) | ∠ (O2–Na1–N2B) | | 87.0(4) |
| ∠ (N3–N3A–N3B) | | 176.9(11) | ∠ (O2–Na1–N3B) | | 81.2(3) |
| ∠ (Au1–N4–N4A) | | 115.1(7) | ∠ (O2–Na1–N3B) | | 85.5(3) |
| ∠ (N4–N4A–N4B) | | 174.2(11) | ∠ (N2B–Na1–N3B) | | 167.9(4) |
|  | |  |  | |  |
| ∠ (Na1–O1–Na1) | | 96.7(3) | ∠ (Na1–O2–Na1) | | 96.3(3) |
| ∠ (H11–O1–H12) | | 135(10) | ∠ (H21–O2–H22) | | 125(10) |

**Table S5.** Crystallographic data for Na[Au(N_3_)_4_]·*n*H_2_O (*n* = 2, 1, 0).

| Composition | Na[Au(N_3_)_4_]·2H_2_O | Na[Au(N_3_)_4_]·H_2_O | Na[Au(N_3_)_4_] |
| --- | --- | --- | --- |
| *n* | 2 | 1 | 0 |
| Refinement method | Single crystal  SHELXL-2018^[2-3]^ | Powder  JANA-2020^[4]^ | Powder  JANA-2020^[4]^ |
| Crystal system | monoclinic | orthorhombic | monoclinic |
| Crystal color, shape | orange transparent prism-like crystals | orange microcristallites | orange microcristallites |
| Space group | *P*2_1_/*c* (No. 14) | *P*2_1_2_1_2_1_ (# 19) | *P*2_1_/*c* (No. 14) |
| *a* [Å]  *b* [Å]  *c* [Å] β [°] | 3.5843(6) 13.037(2) 22.044(3) 92.518(9) | 12.1472(2) 10.9716(2) 6.7600(2) | 7.8139(4) 8.0939(4) 6.9026(4) 111.829(5) |
| *V* [Å^3^] | 1029.1(3) | 900.94(6) | 405.25(4) |
| *Z* | 4 | 4 | 2 |
| *V*/*Z* [Å^3^] | 257.28 | 225.24 | 202.63 |
| Molar mass [g⋅mol^–1^] | 424.11 | 404.05 | 388.00 |
| δ*_x_* [g⋅cm^–3^] | 2.737 | 2.9788 | 3.18 |
| Temperature *T* [K] | 293(2) | 293(1) | 293(1) |
| Diffractometer | Rigaku AFC7 | Stoe Stadi MP | Stoe Stadi MP |
| Detector | CCD Saturn 724+ | DECTRIS MYTHEN2 | DECTRIS MYTHEN2 |
| Radiation, λ [Å] | Mo *K*α, 0.71073 | Cu *K*α_1_, 1.54056 | Cu *K*α_1_, 1.54056 |
| Scan mode |  |  |  |
| 2θ range [°] | 3.6–52.0 | 5.0-109.8 | 4.8–102.3 |
| *hkl* ranges | –4 ≤ h ≤ 3,  –16 ≤ k ≤ 14,  –27 ≤ l ≤ 26 |  |  |
| No. refl. / points | 5461 | 6988 | 4873 |
| No. refl. unique | 2010 |  |  |
| Profile Function |  | Pseudo-Voigt | Pseudo-Voigt |
| F(000) [*e*] | 776 | 776 | 112 |
| μ [mm^–1^] | 14.350 | 31.265 | 34.604 |
| *R*_int_ / *R*_prof_ | 0.0439 | 0.0382 | 0.0595 |
| Refined parameters | 157 | 82 | 42 |
| Restraints |  | 23 | 10 |
| Constraints |  | 1 | 1 |
| *R*1, *wR*2 (all data)  *R*_all_, *wR*_all_ | 0.056, 0.111 | 0.0316, 0.0438 | 0.0426, 0.0496 |
| GOOF | 1.041 | 2.6958 | 1.5717 |
| Highest electron diff. peak | 2.635 | 1.89 | 0.16 |
| Deepest electron diff. hole | –2.102 | –1.12 | –0.16 |

**Table S6a.** Atomic coordinates and isotropic displacement parameters [Å^2^] for Na[Au(N_3_)_4_]·H_2_O. Standard deviations are given in parentheses.

| Atom | Site | *x / a* | *y / b* | *z / c* | *U*_eq/iso_ / Å^2^ |
| --- | --- | --- | --- | --- | --- |
| Au1 | 4*a* | 0.8164(2) | 0.5043(5) | 0.3767(4) | 0.0220(7) |
| N1 | 4*a* | 0.940(3) | 0.629(4) | 0.403(6) | 0.031(5) |
| N1a | 4*a* | 0.031(3) | 0.610(5) | 0.458(4) | 0.036(7) |
| N1b | 4*a* | 0.112(4) | 0.593(5) | 0.506(6) | 0.040(7) |
| N2 | 4*a* | 0.699(3) | 0.646(3) | 0.399(6) | 0.031(5) |
| N2a | 4*a* | 0.604(3) | 0.619(4) | 0.381(6) | 0.036(7) |
| N2b | 4*a* | 0.511(3) | 0.593(4) | 0.363(9) | 0.040(7) |
| N3 | 4*a* | 0.692(4) | 0.379(3) | 0.350(6) | 0.031(5) |
| N3a | 4*a* | 0.699(5) | 0.272(3) | 0.336(5) | 0.036(7) |
| N3b | 4*a* | 0.705(4) | 0.172(3) | 0.323(7) | 0.040(7) |
| N4 | 4*a* | 0.934(4) | 0.363(4) | 0.354(6) | 0.031(5) |
| N4a | 4*a* | 0.926(5) | 0.269(3) | 0.440(4) | 0.036(7) |
| N4b | 4*a* | 0.919(4) | 0.182(4) | 0.519(7) | 0.040(7) |
| Na1 | 4*a* | 0.3136(18) | 0.495(4) | 0.394(3) | 0.0319(13) |
| O1 | 4*a* | 0.823(4) | 0.892(3) | 0.306(5) | 0.024(9) |

**Table S8a.** Selected interatomic distances [Å] and bond angles [°] in Na[Au(N_3_)_4_]·H_2_O with their multiplicity (*n*). Standard deviations are given in parentheses.

| Atoms |  | *d* [Å] | Atoms |  | *d* [Å] |
| --- | --- | --- | --- | --- | --- |
| Au1− | Au1 2x | 3.746(4) | Na1 | Na1 2x | 3.72(3) |
|  |  |  |  |  |  |
| Au1− | N1 | 2.04(4) | Na1− | O1 | 2.42(5) |
|  | N2 | 2.12(4) |  | O1 | 2.38(4) |
|  | N3 | 2.05(4) |  | N1b | 2.78(6) |
|  | N4 | 2.11(5) |  | N1b | 2.94(5) |
|  |  |  |  | N2b | 2.64(5) |
| N1– | N1a | 1.18(5) |  | N3b | 2.44(5) |
| N1a– | N1b | 1.05(6) |  | N3b | 2.96(5) |
| N2– | N2a | 1.20(5) |  | N4b | 2.40(6) |
| N2a– | N2b | 1.17(5) |  |  |  |
| N3– | N3a | 1.18(5) |  |  |  |
| N3a– | N3b | 1.10(5) |  |  |  |
| N4– | N4a | 1.19(5) |  |  |  |
| N4a– | N4b | 1.10(5) |  |  |  |
|  |  | ° |  |  | ° |
| ∠ (Au1–Au1– Au1) | | 128.90(10) | ∠ (Na1– Na1– Na1) | | 130.8(7) |
|  | |  |  | |  |
| ∠ (N1–Au1–N2) | | 89.8(15) | ∠ (Au1–N1–N1a) | | 127(3) |
| ∠ (N1–Au1–N3) | | 179.9(16) | ∠ (N1–N1a–N1b) | | 180(5) |
| ∠ (N1–Au1–N4) | | 90.0(17) | ∠ (Au1–N2–N2a) | | 117(3) |
| ∠ (N2–Au1–N3) | | 90.1(14) | ∠ (N2–N2a–N2b) | | 180(4) |
| ∠ (N2–Au1–N4) | | 179.9(16) | ∠ (Au1–N3–N3a) | | 128(3) |
| ∠ (N3–Au1–N4) | | 90.0(16) | ∠ (N3–N3a–N3b) | | 180(4) |
|  | |  | ∠ (Au1–N4–N4a) | | 123(3) |
| ∠ (Na1–O1–Na1) | | 101.5(15) | ∠ (N4–N4a–N4b) | | 180(4) |
|  | |  |  | |  |
| ∠ (O1–Na1–O1) | | 138.8(16) | ∠ (N1b-Na1-N2b) | | 131.7(15) |
| ∠ (O1–Na1–N1b) | | 74.3(16) | ∠ (N1b-Na1-N3b) | | 77.0(15) |
| ∠ (O1–Na1–N1b) | | 63.8(13) | ∠ (N1b-Na1-N3b) | | 75.7(16) |
| ∠ (O1–Na1–N2b) | | 140.3(17) | ∠ (N1b-Na1-N4b) | | 135.8(19) |
| ∠ (O1–Na1–N3b) | | 88.4(17) | ∠ (N1b-Na1-N2b) | | 77.5(16) |
| ∠ (O1–Na1–N3b) | | 78.1(15) | ∠ (N1b-Na1-N3b) | | 70.8(15) |
| ∠ (O1–Na1–N4b) | | 100.8(18) | ∠ (N1b-Na1-N3b) | | 120.7(14) |
| ∠ (O1-Na1-N1b) | | 67.0(14) | ∠ (N1b-Na1-N4b) | | 77.8(16) |
| ∠ (O1-Na1-N1b) | | 156.6(15) | ∠ (N2b-Na1-N3b) | | 73.3(15) |
| ∠ (O1-Na1-N2b) | | 79.1(17) | ∠ (N2b-Na1-N3b) | | 135.2(15) |
| ∠ (O1-Na1-N3b) | | 95.9(15) | ∠ (N2b-Na1-N4b) | | 82.2(18) |
| ∠ (O1-Na1-N3b) | | 76.5(13) | ∠ (N3b-Na1-N3b) | | 147.0(16) |
| ∠ (O1-Na1-N4b) | | 97.2(16) | ∠ (N3b-Na1-N4b) | | 147.1(20) |
| ∠ (N1b-Na1-N1b) | | 130.0(15) | ∠ (N3b-Na1-N4b) | | 65.1(14) |

**Table S6b.** Atomic coordinates and isotropic displacement parameters [Å^2^] for Na[Au(N_3_)_4_]. Standard deviations are given in parentheses.

| Atom | Site | *x / a* | *y / b* | *z / c* | *U*_eq/iso_ / Å^2^ |
| --- | --- | --- | --- | --- | --- |
| Au1 | *2c* | 0 | ½ | 0 | 0.0115(12) |
| N1 | 4*e* | 0.273(2) | 0.507(4) | 0.1982(16) | 0.011(9) |
| N1a | 4*e* | 0.327(4) | 0.610(3) | 0.317(4) | 0.004(11) |
| N1b | 4*e* | 0.395(4) | 0.718(3) | 0.440(5) | 0.006(11) |
| N2 | 4*e* | 0.046(4) | 0.245(2) | -0.014(3) | 0.005(7) |
| N2a | 4*e* | 0.171(3) | 0.171(3) | 0.087(4) | 0.005(7) |
| N2b | 4*e* | 0.299(4) | 0.097(4) | 0.209(5) | 0.043(14) |
| Na1 | 2*d* | ½ | 0 | ½ | 0.007(5) |

**Table S7b.** Anisotropic displacement parameters [Å^2^] for Na[Au(N_3_)_4_]. Standard deviations are given in parentheses.

| Atom | *U*_11_ / Å^2^ | *U*_22_ / Å^2^ | *U*_33_ / Å^2^ | *U*_23_ / Å^2^ | *U*_13_ / Å^2^ | *U*_12_ / Å^2^ |
| --- | --- | --- | --- | --- | --- | --- |
| Au1 | 0.0164(17) | 0.0097(17) | 0.0084(18) | 0.003(6) | 0.0045(14) | 0.004(7) |

**Table S8b.** Selected interatomic distances [Å] and bond angles [°] in Na[Au(N_3_)_4_] with their multiplicity (*n*). Standard deviations are given in parentheses.

| Atoms |  | *d* [Å] | Atoms |  | *d* [Å] |
| --- | --- | --- | --- | --- | --- |
| Au1− | N1 2x | 2.062(13) | Na1− | N1 2x | 2.612(17) |
|  | N2 2x | 2.103(17) |  | N1b 2x | 2.41(2) |
|  |  |  |  | N2b 2x | 2.18(3) |
| N1− | N1a | 1.14(3) |  |  |  |
| N1a− | N1b | 1.20(4) |  |  |  |
| N2− | N2a | 1.14(3) |  |  |  |
| N2a− | N2b | 1.20(4) |  |  |  |
|  |  |  |  |  |  |
| ∠ (N1–Au1–N2) 2x | | 84.6(11) | ∠ (N1–Na1–N1) | | 180 |
| ∠ (N1–Au1–N2) 2x | | 95.4(11) | ∠ (N1–Na1–N1b) 2x | | 79.4(11) |
| ∠ (N1–Au1–N1) | | 180 | ∠ (N1–Na1–N1b) 2x | | 100.6(11) |
| ∠ (N2–Au1–N2) | | 180 | ∠ (N1–Na1–N2b) 2x | | 84.7(11) |
|  | |  | ∠ (N1–Na1–N2b) 2x | | 95.3(11) |
| ∠ (Au1–N1–N1a) | | 121(2) | ∠ (N1b–Na1–N1b) | | 180 |
| ∠ (N1–N1a–N1b) | | 175(4) | ∠ (N1b–Na1–N2b) 2x | | 84.3(10) |
| ∠ (Au1–N2–N2a) | | 127.7(18) | ∠ (N1b–Na1–N2b) 2x | | 95.7(10) |
| ∠ (N2–N2a–N2b) | | 174(4) | ∠ (N2b–Na1–N2b) | | 180 |

**Hydrogen bonds**

**
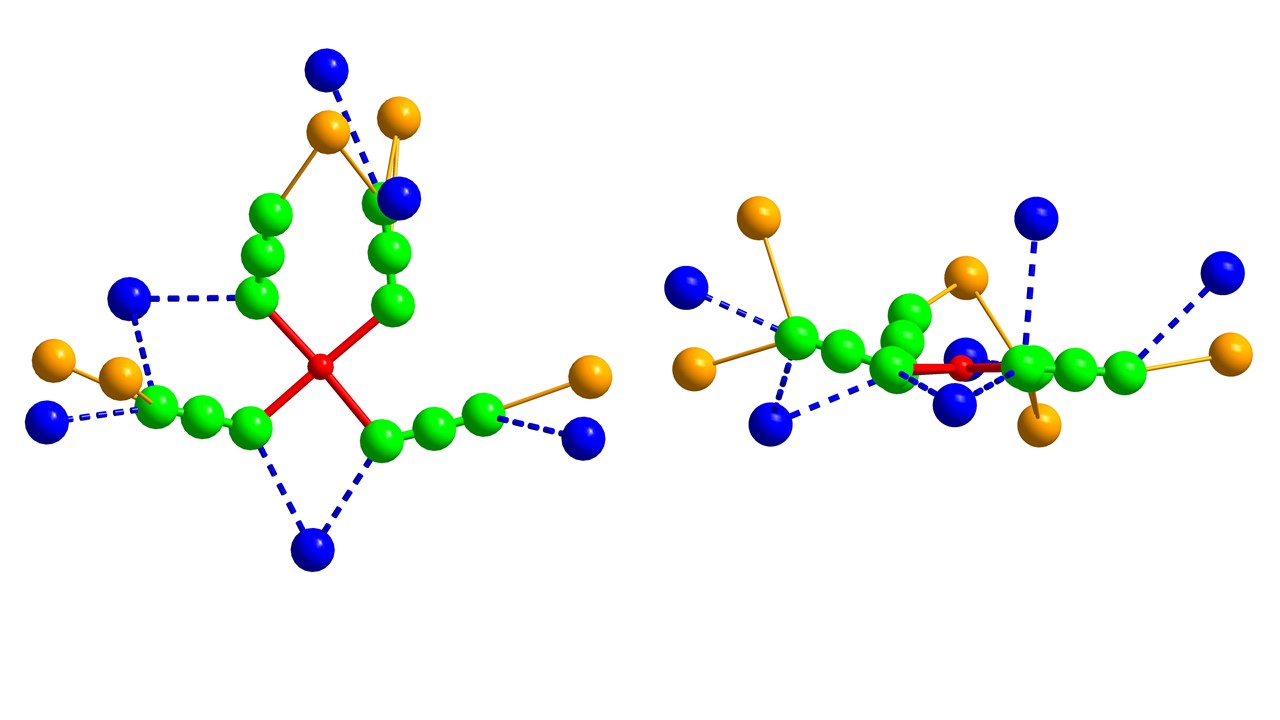
**The H positions in Na[Au(N_3_)_4_]_2_·H_2_O could not be refined from powder X-ray data.

**Figure S7.** The environment of azidoaurate anion [Au(N_3_)_4_]^–^ in Na[Au(N_3_)_4_]_2_·H_2_O. View perpendicular (left) and through the AuN_4_ plane (right). Contacts to O with distances 289 pm < *d*(N–O) < 339 pm (blue dotted lines) indicate potential moderate or weak hydrogen bonds.^[22]^

Spectroscopy

**Vibrational Spectra of Na[AuCl_4–_*_x_*(N_3_)*_x_*]⋅2H_2_O (*x* = 1, 2, 3)**

The vibrational spectroscopically relevant units in the three sodium chloridooazidoaurate compounds are the mixed anions [AuCl_4–_*_x_*(N_3_)*_x_*]·2H_2_O (*x* = 1–3) for which 15 (*x* = 1, Na[AuCl_3_(N_3_)]·2H_2_O (**1**)), 21 (*x* = 2, Na[AuCl_2_(N_3_)_2_]·2H_2_O (**2**)) and 27 (*x* = 3, Na[AuCl(N_3_)_3_]·2H_2_O (**3**)) modes are expected, respectively. Regarding the basic square-planar geometry of the anionic species, one can predict for the local symmetry of the central gold atoms with their Cl or N neighbors in ratios of 3:1 (i.e. three Cl and one N or vice versa) or 2:2 (i.e. two Cl and two N) coordinations with the idealized point symmetries *C*_s_/*m* and *C*_2_*_h_*/2*m*, respectively. But taking into account that those nitrogen atoms actually are stemming from the tri-atomic azide units, the real symmetry (site-symmetry) is in all cases lowered, due to the different spatial orientation of the azide groups. The symmetry reduction, i.e. the site symmetry, can be directly obtained from the Wyckoff symmetry of the central Au atoms in the space group of each coordination compound and corresponds to *C*_s_/*m*, *C*_i_/–1, and *C*_s_/*m*, for [AuCl_3_(N_3_)]^–^ (**1**), [AuCl_2_(N_3_)_2_]^–^ (**2**) and [AuCl(N_3_)_3_]^–^ (**3**), respectively.

The Raman and FTIR spectra of the mixed chloridooazidoaurates of sodium are presented in Figure S8, alongside those of Na[AuCl_4_] and Na[Au(N_3_)_4_] for comparison. The Raman spectra (Figures S8a+b) can be divided into three sections, represented by (Au–Cl) (50-350 cm^–1^), (Au–N) (200-1300 cm⁻¹), and (N=N=N) (500-2300 cm^–1^) modes. Given that the observable lower wave region of the currently employed FTIR spectrometer is limited to 450 cm^–1^, the chloridoaurate bands cannot be detected in the IR spectrum, but only in the Raman spectra. However, the presence of Au–Cl bonds will influence the geometry in such a way that the symmetry of the complex anion changes. This alteration will then also affect the appearance and patterning of the (Au–N) and (N=N=N) Raman bands, which are typically observed as band splittings and violations of selection rules both for Raman-allowed and IR-active modes in the spectra. It is interesting to note, that in IR spectra of mixtures [AuCl_4–_*_x_*(N_3_)*_x_*]·2H_2_O (0 ≤ *x* ≤ 4) always only modes of two neighboring phases may be discerned (Figure S11).

The point group symmetry *D*_4_*_h_*/4/*mm* of the the tetrachloridoaurate moieties in Na[AuCl_4_]·2H_2_O predicts a total of 9 modes whereby *A*_1g_, *B*_1g_ and *E*_u_ are stretching, and *B*_2g_, *A*_2u_, 2*B*_2u_ and 2*E*_u_ are bending vibrations. The three Raman-active *g*-modes are registered at 346, 326 and 180 cm^–1^ (Figure 11b top). The most intense Raman peak at 346 cm^–1^ is the breathing mode which is characteristic and can be used as a probe for detection of Au–Cl bonds. With the successive substition of the Cl ligands by azide groups in the coordination compounds (**1**) – (**3**) the characteristic ν(Au–Cl) shifts slightly to lower wavenumbers (348 cm^–1^ → 327 cm^–1^) accompanied by a decrease in intensities due to the decline in the number of Cl ligands. Accordingly, the idealized symmetry changes from *D*_4h_ (“AuCl_4_”) over *C*_s_ (“AuCl(N_3_)_3_”) to *D*_2h_ (trans “AuCl_2_(N_3_)_2_”) and *C*_s_ (“AuCl(N_3_)_3_”). Despite the symmetry reduction, the planarity of the anions is maintained in all cases.

In detail, the Raman spectra of the series (**1**) – (**3**) show particularly in the region 300–450 cm^–1^ distinct differenct characteristic (Au–Cl) and (Au–N) stretches. (**1**) has 3 (Au–Cl) bonds, accordingly there are two Raman modes representing symmetric and antisymmetric modes. In Na[AuCl_2_(N_3_)_2_]⋅2H_2_O there are also two chloridoaurate bonds, but due to the *cis-*configuration and the quasi-*C*_2h_ symmetry of the anion, the antisymmetric valence vibration becomes IR active and, thus, only the symmetric stretch will be observed in the Raman spectrum. Finally, in (**3**) there is only one (Au–Cl) bond giving rise to one Raman signal. Likewise, the appearance and wavenumbers for the (Au–N) bands in the complex series (**1**-**3**) are very similar, and they are recorded as a doublet in [AuCl(N_3_)_3_]^–^ and single bands in **1** and **2**, respectively. The higher frequency bands between 650–2100 cm^–1^ are stemming entirely from the normal vibrations of the “quasi-free” azide moieties^[23-25]^ in combination to their motions with respect to each other. In detail, the weak peaks at 689, 685 and 681 cm^–1^ in **1**, **2** and **3**, respectively, are assigned to out-of-plane deformation of the N_3_ moities, as observed in the free azide anion. In the same order the weak peaks at 1228, 1272 and 1248/1280 cm^–1^, as well as those at 2038, 2069 and 2032/2068 cm^–1^ are representing the symmetric and antisymmetric streches of the azide ligands. The Raman bands below 300 cm^–1^ are either (N–N–N), (Au–N–N) and (Au–Cl) deformations and torsions.

Finally, the FTIR spectra of **1**-**3** are shown in Figure 11c alongside that of Na[Au(N_3_)_4_]⋅2H_2_O for comparison. The infrared spectrum of sodium tetrachloridoaurate is not depicted as the frequency range of its normal modes (50–350 cm^–1^) lies outside of the available measurement range (450–4000 cm^–1^) of the spectrometer used. Compared to Raman spectra in Figures 11a,b two major differences are notable:

1) There is group of peaks of medium to strong intensity located at approximately 1620 cm^–1^ (i.e. 1627 cm^–1^ (**3**), 1634 cm^–1^ (**2**), and 1620 cm^–1^ (**1**)), as well as a strongly paterned broad multiplet around 3500 cm^–1^ (i.e. 3274, 3376, 3447, 3563, 3585 cm^–1^) (**3**), (3236, 3305, 3498, 3566 cm^–1^) (**2**), and (3254, 3407, 3524, 3581 cm^–1^) (**3**) which arise from the vibrations of the hydrated water molecules. Due to the polarity of hydrogen bonds, these peaks are very weak and could hardly be detected in their Raman spectra. Thus, only the OH valence mode around 3500 cm^–1^ could be recorded as a broad and very weak Raman band in **1**–**3**. Assuming that those bands alone have a very limited conclusiveness – and the clear proof for the presence of hydrated water would be delivered by the IR spectra anyway – the, Raman spectra were cut off > 2500 cm^–1^.

2) In the Raman spectra of **1**–**3** between 1000 and 2500 cm^–1^ there are only two weak to very weak peaks already attributed to the symmetric and antisymmetric stretches of the azide groups. Their pendants in IR are located as strong and very strong absorptions at (1232, 1271 cm^–1^) (**1**); 1269 cm^–1^ (**2**), and 1251, 1277 cm^–1^ (**3**) and at 2048 cm^–1^ (**1**), 2047 cm^–1^ (**2**), and 2031 cm^–1^ (**3**), respectively. This confirms that the dipole moment change during these vibrations is considerably strong. As for the Raman spectra, the peak at 1269 cm^–1^ is not split and appears as single line, due to the “quasi-*D*_2h_” symmetry of the of the anion (**2**).

The out-of-plane motions of the linear (N=N=N) units were monitored in Raman around 685 cm^–1^, their IR counterpart is only detectable in (**3**) at 625 cm^–1^. The peaks at 574 cm^–1^ (**2**) and 562, 570 cm^–1^ (**1**) correspond to antisymmetric in-plane deformation. The remaining weak to very weak absorptions at 910, 1372, 2492 cm^–1^ (**3**), 1364, 2533 cm^–1^ (**2**) and 1381, 2453, 2532 cm^–1^ (**3**) cannot be fundamentals and are interpreted as combinations or overtones, since – except for the peak at 910 cm^–1^ – the measured wavenumbers are very similiar in all three compounds.

**Vibrational Spectra of Na[Au(N_3_)_4_]⋅2H_2_O**

Na[AuN_12_]⋅2H_2_O crystallizes in space group *P*2_1_/*c* (# 14) with *Z* = 8. The spectroscopically relevant units are the discrete [Au(N_3_)_4_]^–^ moieties with the idealized symmetry *C*_4_*_h_* for which following fundamentals are expected:

*G*_vib_ = 5*A*_g_ (RE) + 5*B*_g_ (RE) + 2*E*_g_ (RE) + 3*A*_u_ (IR) + 3*B*_u_ (-,-) + 6*E*_u_ (IR).

However, in crystalline Na[Au(N_3_)_4_]·2H_2_O, there is one crystallographically distinct azidoaurate anion which experiences a symmetry reduction to *C*_1_/1 (site-symmetry) with the effect that all modes become now active in both RE and IR, the double generate *E*_g_ and *E*_u_ split up and the forbidden *B*_u_ will be observable in both spectra. In addition, the primitive unit cell contains 8 formula units of azidoaurate anions which can couple with each other giving rise to additional band splittings (factor group splitting). Thus, one may expect for Na[Au(N_3_)_4_]·2H_2_O quite complex and strongly patterned vibrational spectra, particularly well recognizable in the IR spectrum. The reason for the latter is that – in contrast to the weak Raman peaks – the IR modes stemming from the azide groups are simply more intense and dominant. In good agreement with the predictions, the vibrational spectra are strongly split and the number of observed bands speaks clearly for the fact that both site-symmetry and factor group splitting have taken place.

The vibrational spectra of azidoaurates *A*[Au(N_3_)_4_] (*A* = K, Rb, Cs), as well as those of Ba[Au_2_(N_3_)_4_]·4H_2_O have been discussed in detail recently.^[26-27]^ Therefore we want to point out only the significant spectroscopic differences and features between the novel Na[Au(N_3_)_4_]·2H_2_O (Figure S9) and the afore mentioned *A*[Au(N_3_)_4_] series. Of specific interest are the stretching (Au–N), (Au–**N–N**≡N) and (Au–N–**N≡N**) modes which are indicative for the respective bond strengths. The symmetric stretch ν(Au–N) is observed as the strongest band of the Raman spectrum at 414 cm^–1^ with a shoulder at 397 cm^–1^ representing the antisymmetric valence vibration of the square planar [AuN_4_] units. These two bands could only be registered in the Raman spectrum since their values are lying below the measuring range of the ATR-spectrometer (i.e. 450–4000 cm^–1^) used. The group of bands appearing between 1250-1285 cm^–1^ in both spectra are resulting from the symmetric in-phase (←**N–N→**≡N) stretch of the longer inner (N–N) bonds of the azide groups. They are usually observed as a single peak or doublet, emerging now – unlike the former examples – as intense and weak triplets in IR and RE, respectively, due to factor group splitting. The corresponding in-phase vibrations of the two outermost N atoms with shorter bond lengths (N**←N≡N**→) are located between 2020–2080 cm^–1^ as strong and medium intense triplets at 2023, 2048, 2069 cm^–1^ (IR) and 2029, 2054, 2075 cm^–1^ (RE), respectively. The medium to weak absorptions at 692, 606 and 574 cm^–1^ and the Raman peaks at 682 and 688 cm^–1^ are assigned to in-plane bending modes of the azide moieties. Finally, the region < 250 cm^–1^ is dominated by (N–Au–N) and (Au–N–N) deformations and torsions, as well as lattice vibrations.

Worth mentioning are also the two groups of strong to very strong absorptions registered as a doublet at 1628 and 1653 cm^–1^ and a multiplet at 3274, 3376, 3447, 3565 and 3585 cm^–1^. They are stemming from the bending (scissoring) motions and antisymmetric stretch of the two distinct oxygen atoms (i.e. hydrate water molecules) per formula unit. Based on these facts, one would predict for the two hydrate molecules the presence of a pair of two bands in the IR spectrum, differing only slightly in their wavenumbers. The doublet at 1628 and 1653 cm^–1^ fulfills these expectations whereas the “doublet” of the (O–H) stretch is further split to give a quintet. The strong patterning of the latter is due to the presence of the 2×8 interacting equivalent water molecules in the unit cell (factor group splitting).

The remaining weak IR peaks at 1385, 2496 and 2555 cm^–1^ are interpreted as combination bands; i.e. 2×692 = 1384 cm^–1^, 2×1253 = 2506 cm^–1^ and 1270 + 1284 = 2554 cm^–1^_,_ respectively.

**Spectra of Na[Au(N_3_)_4_]·*n*H_2_O (*n* = 2, 1, 0)**

Upon heating or employing vacuum, Na[Au(N_3_)_4_]·2H_2_O loses its waters of crystallization in two steps, finally yielding anhydrous sodium azidoaurate Na[Au(N_3_)_4_], The evolution of the structural changes in Na[Au(N_3_)_4_]·*n*H_2_O (*n* = 2, 1, 0) going from sodium tetraazidoaurate dihydrate to the anhydrous salt was followed by IR spectroscopy (Figures S10, S12). The IR spectrum of the compound with *n* = 2 is also depicted in this Figure for comparison. The compounds Na[Au(N_3_)_4_]·*n*H_2_O with *n* = 1 and 0 crystallize orthorhombic in space group *P*2_1_2_1_2_1_ with *Z* = 4 and monoclinic in space group *P*2_1_/*c* with *Z* = 2, respectively. From the gold positions we can directly derive the local geometry (site-symmetry) of the tetraazidoaurate units complexes: 1/*C*_1_ and –1/*C*_i_ for *n* = 1 and 0, respectively. Despite comprising exactly the same anions, the three spectra for *n* = 2, 1, 0 look significantly different, which cannot be explained by symmetry effects alone. At first glance, the IR measurement results (Figure 1b) reveal for the monohydrate and the anhydrous compound less patterened spectra which can be explained by the strong factor group splitting observed for Na[Au(N_3_)_4_]·2H_2_O. For *Z* = 4 and *Z* = 2 for *n* = 1 and 0 a factor group splitting is also expected, but its extent is obviously too weak to be detected.

An important detail is the change of the intensities of the bands at around 1600 and 3500 cm^–1^ which are attributed to water of crystallization. As expected, these bands lose their intensities successively by transitioning from *n* = 2 to 0, ultimately disappearing completely in Na[Au(N_3_)_4_]. In accordance with site symmetry analyses, the IR spectrum for the anhydrous salt is less patterned since only u modes are IR active, due to the presence of a symmetry center in *C*_i_. The tetraazidoaurate anion in the monohydrate exhibits *C*_1_ site-symmetry, thus, all modes are both Raman and IR active, meaning that we expect a more patterned IR spectrum, as it is the case. Except for the hydrate water peaks at 1600 and 3500 cm^–1^, there are no other vanishing or emerging bands, which are characteristic for fundamental structural or geometrical changes. The observed band splittings and multiplets result from the splittings of the fundamental vibrations of the [Au(N_3_)_4_]^–^ moities due to the symmetry reduction from the ideal symmetry *C*_4h_ to the respective site-symetries 1/*C*_1_ and –1/*C*_i_. The assignment of the peaks in Na[Au(N_3_)_4_]⋅*n*H_2_O (*n* = 1, 0) is practically the same as for Na[Au(N_3_)_4_]⋅2H_2_O previously discussed.


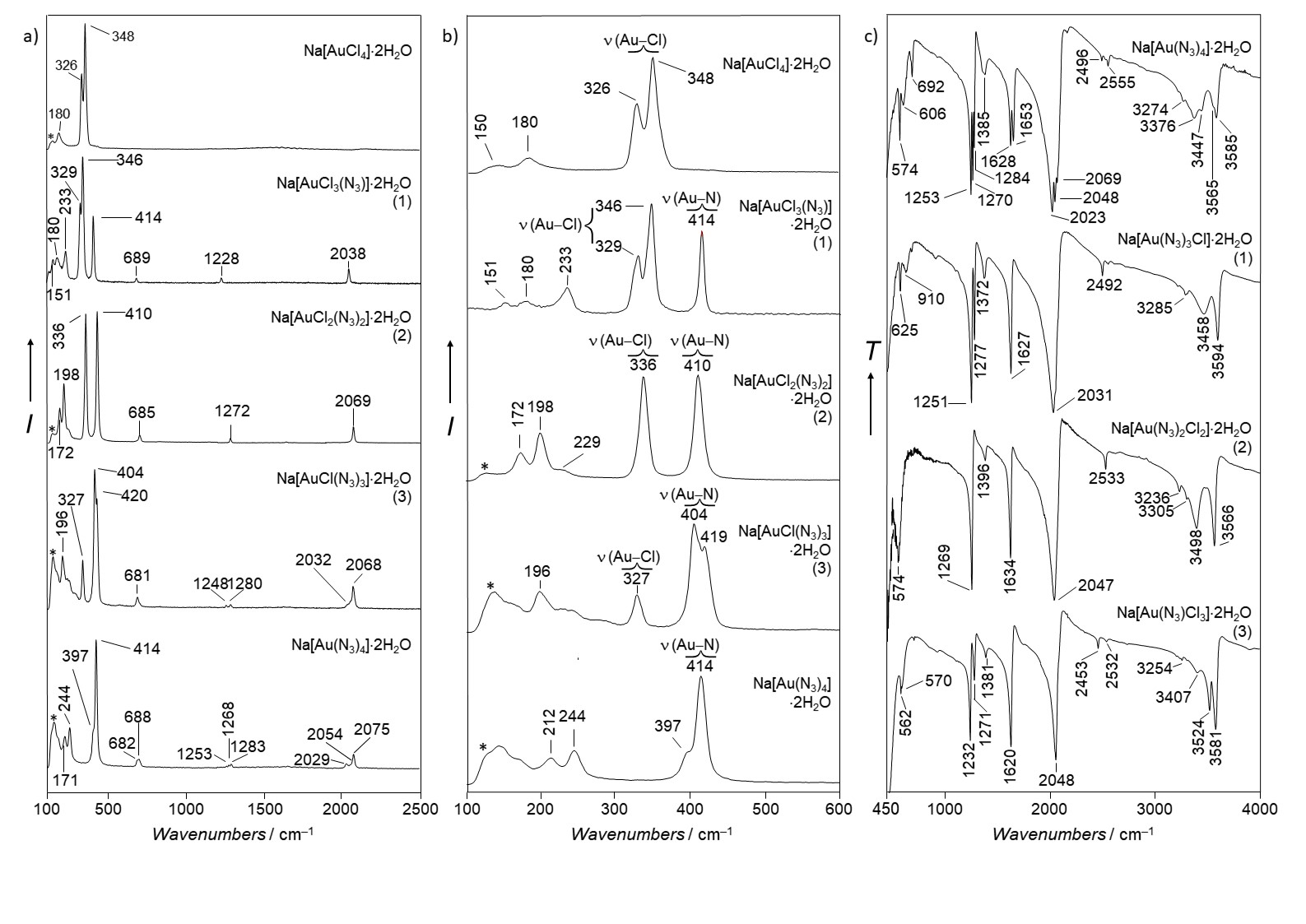


**Figure S8.** Vibrational spectra of Na[AuCl_4–_*_x_*(N_3_)*_x_*]·2H_2_O (*x* = 0, 1, 2, 3, 4): a) Raman spectra in the region 100–2500 cm^–1^, b) Detail of Raman spectra in the region 100–600 cm^–1^, c) IR spectra in the region 400–4000 cm^–1^. Intensity (Raman) and transmission (IR-ATR) in arbitrary units. For experimental conditions see text.

**
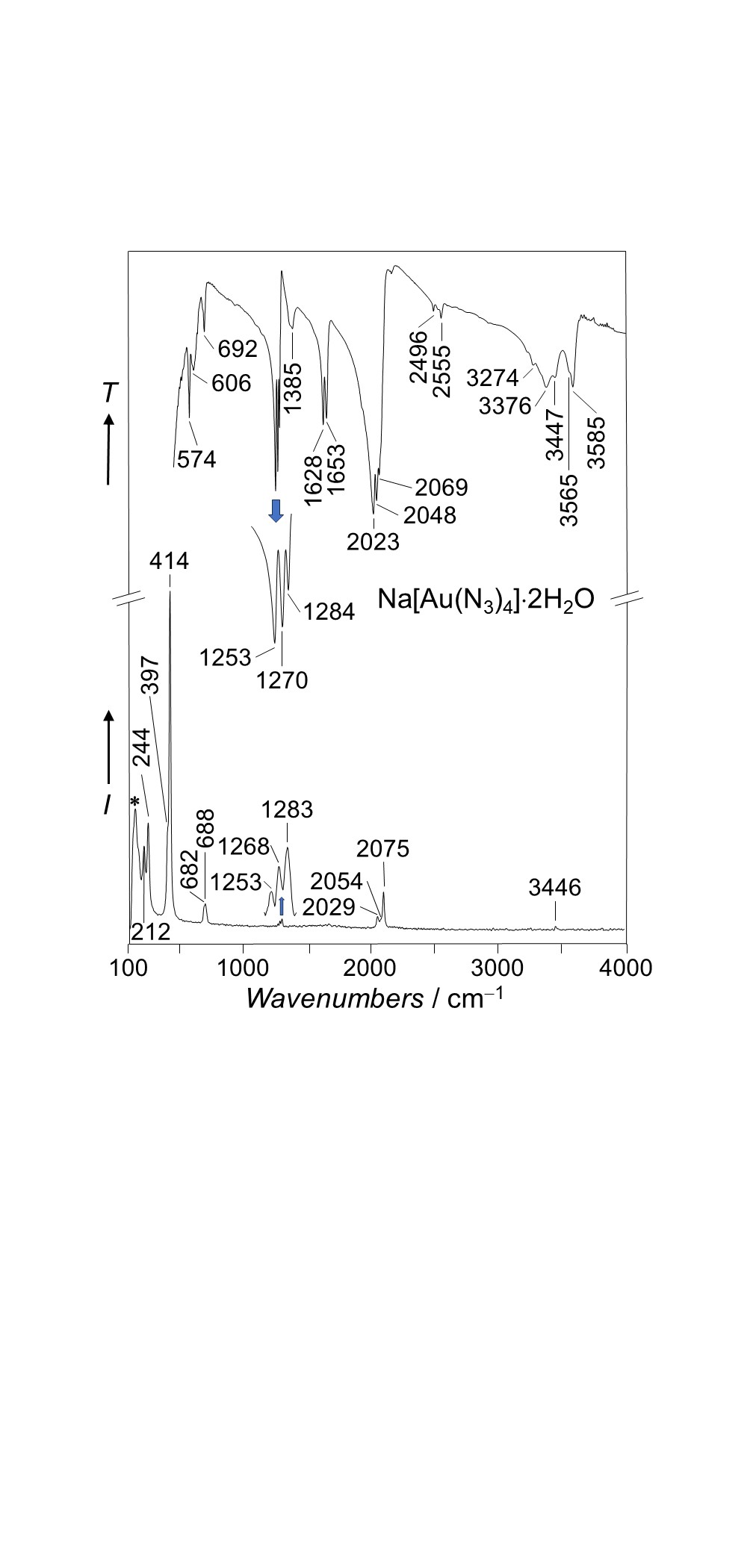
**

~~
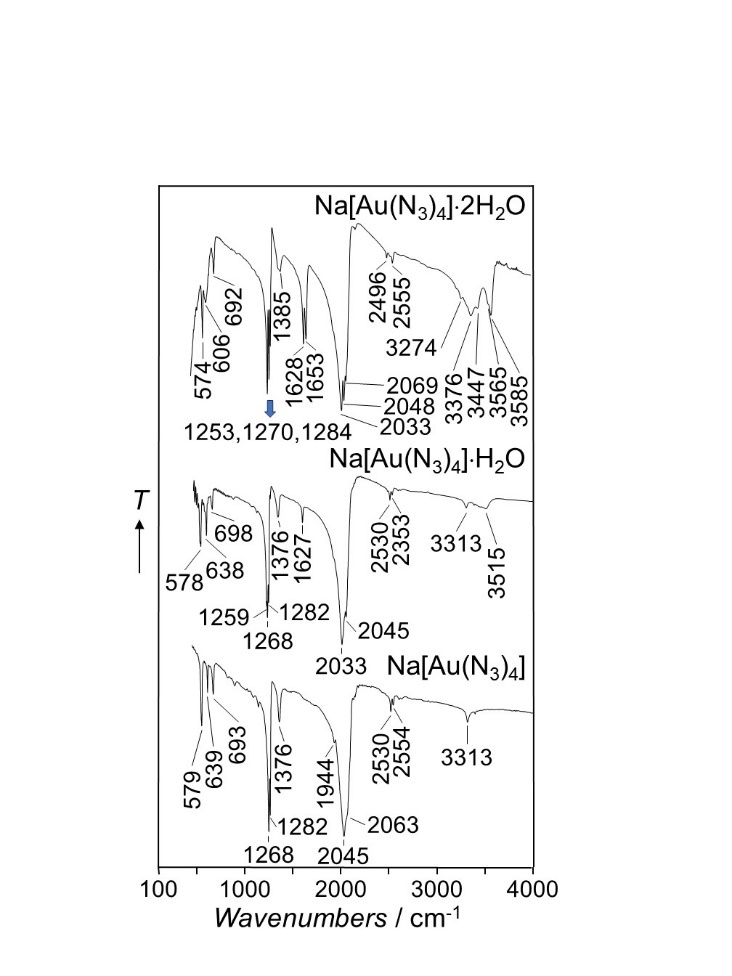
~~**Figure S9.** Vibrational spectra of Na[Au(N_3_)_4_]·2H_2_O. Intensity (Raman) and transmission (IR-ATR) in arbitrary units.

**Figure S10.** IR spectra of Na[Au(N_3_)_4_]·2H_2_O, Na[Au(N_3_)_4_]·H_2_O and Na[Au(N_3_)_4_]. Transmission (IR-ATR) in arbitrary units.

**
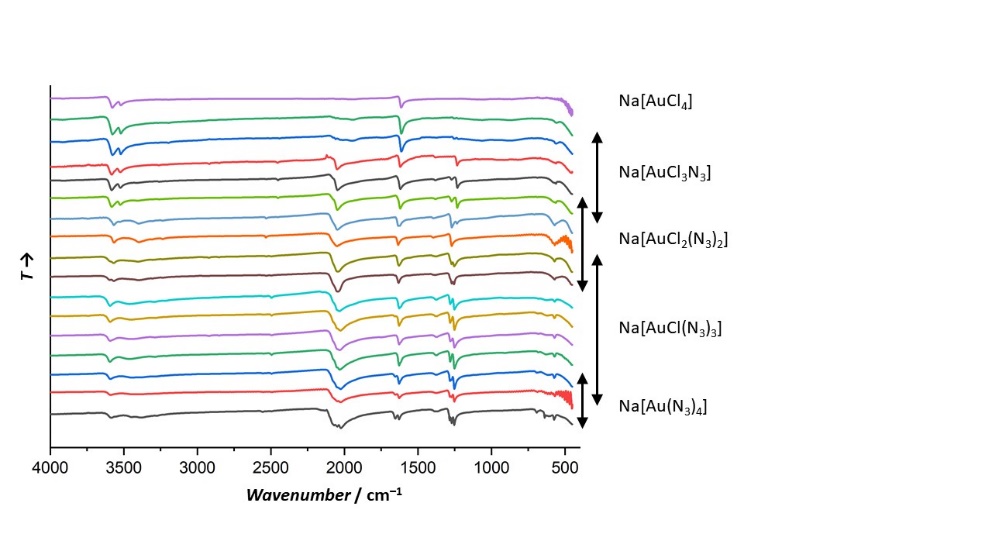
**

**Figure S11.** IR spectra of mixtures of Na[AuCl_4–_*_x_*(N_3_)*_x_*]·2H_2_O (0 ≤ *x* ≤ 4). Transmission in arbitrary units.

**
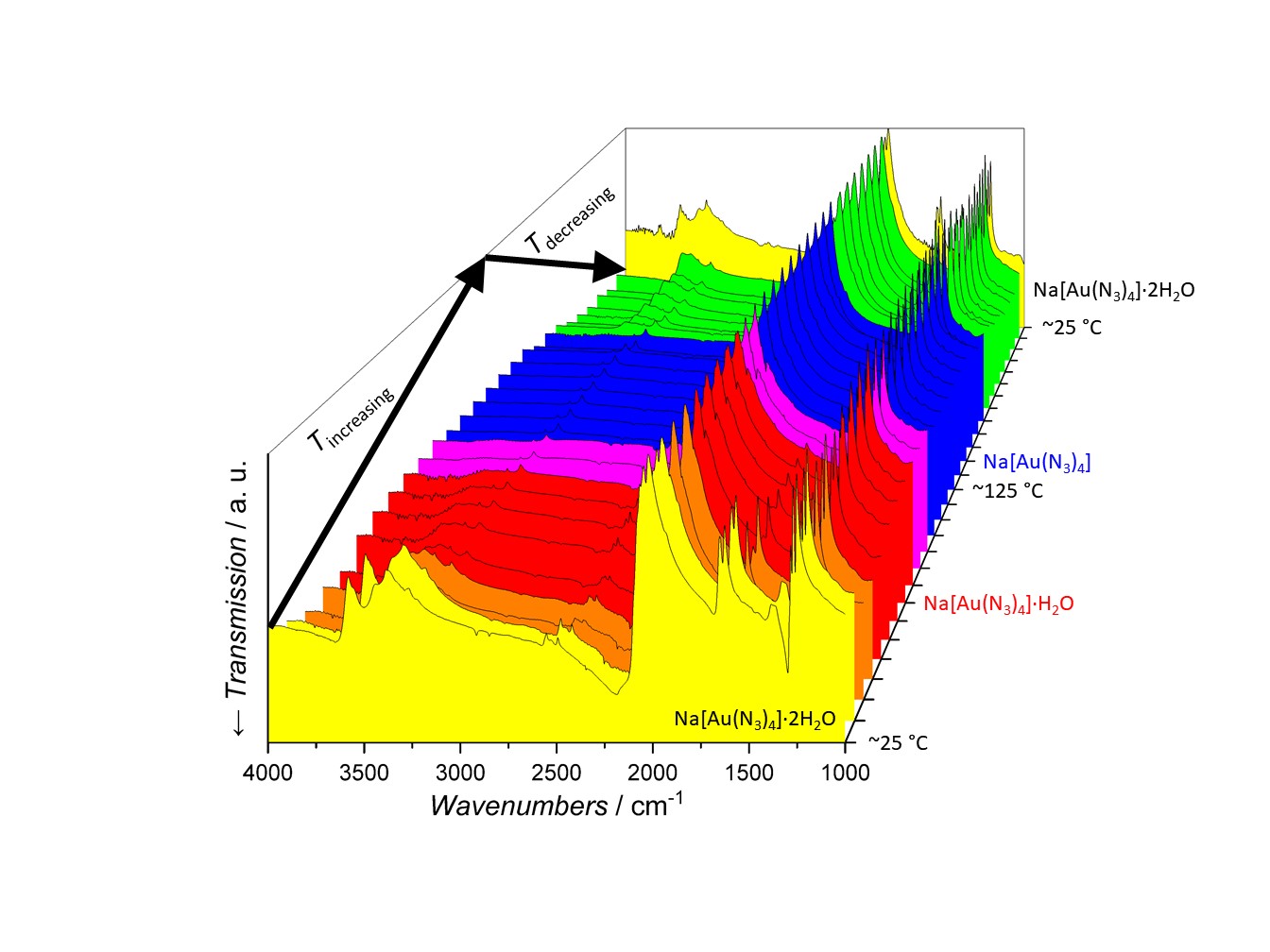
Figure S12.** IR spectra (in non-standard representation with transmission going down) of a heating measurement up to 125 °C of a sample of Na[Au(N_3_)_4_]·2H_2_O (yellow) showing the transition to Na[Au(N_3_)_4_]·H_2_O (red) and Na[Au(N_3_)_4_] (blue) and back. Mixed colors (orange, pink, green) indicate presence of multiple phases. Na[Au(N_3_)_4_]·H_2_O is not observed upon cooling.

# DFT calculations

Density Functional Theory (DFT) calculations were performed to investigate the chemical and structural properties of the [AuCl_4–_*ₓ*(N_3_)*ₓ*]^–^ complexes (*x* = 0, 1, 2, 3, 4) shown in Figure 14. The lengths of Au–N bonds, Au–Cl bonds, and N–N bonds within azide ligands in the studied complexes and their corresponding force constants are given in Table S9.

Analyzing Au–N bonds in azide-containing complexes ([AuCl_3_(N_3_)]^–^, [AuCl_2_(N_3_)_2_]^–^, [AuCl(N_3_)_3_]^–^, and [Au(N_3_)_4_]^–^) is important for revealing the structural and dynamic properties. As can be seen from Table S9, the Au–N bond lengths vary between approximately 2.079 Å and 2.114 Å, and the corresponding force constants vary between 1.754 Ncm^–1^ and 2.009 Ncm^–1^. These variations are due to differences in bond strength depending on the number and position of the ligands (chloride or azide) bound to the gold center. Longer Au–N bonds are typically associated with lower force constants and represent weaker bonds.

In chloride-containing complexes ([AuCl_4_]^–^, [AuCl_3_(N_3_)]^–^, [AuCl_2_(N_3_)_2_]^–^, and [AuCl(N_3_)_3_]^–^), Au–Cl bond lengths range from 2.430 Å to 2.464 Å, while force constants range from 1.456 Ncm^–1^ to 1.739 Ncm^–1^. The results in Table S9 show that Au–Cl bonds are generally longer and have lower force constants than Au–N bonds. This indicates that Au–Cl bonds are typically weaker than Au–N bonds. This difference is the result of nitrogen having a higher electronegativity and smaller atomic radius compared to chlorine, leading to a more favorable orbital overlap and stronger covalent character in the Au–N bond.

The N–N bonds in the azide ligands exhibit two distinct and remarkably consistent features in all azide-containing complexes studied. An examination of Table S9 reveals two separate sets of bond lengths and force constants for N–N bonds in azide ligands. In the first set, N–N bond lengths range from approximately 1.245 Å to 1.250 Å, and force constants range from 9.477 Ncm^–1^ to 9.961 Ncm^–1^. In the second set, N–N bond lengths range from approximately 1.181 Å to 1.188 Å, and force constants range from 14.076 Ncm^–1^ to 14.786 Ncm^–1^. This clear distinction between two sets of values arises from electron delocalization resulting from the resonance structures and the asymmetric nature of the azide (N_3_) ligand. The shorter N–N bond (approximately 1.185 Å) has a significantly higher force constant (approximately 14.431 Ncm^–1^) likely corresponds to a triple bond, which is stronger than a double bond but slightly weaker than a triple bond. In contrast, the longer N–N bond (approximately 1.248 Å) has a lower force constant (approximately 9.719 Ncm^–1^), indicating that the bond is stronger than a typical single bond but slightly weaker than a double bond. The internal bond structure of the azide ligand consistently exhibits two distinct bonding characteristics, regardless of the molecular environment. This finding is critical for understanding the intrinsic stability and reactivity of these azido complexes. However, the overall electronic effects of other ligands coordinating with the Au center may slightly modulate the absolute lengths and force constants of the N–N bonds.

In conclusion, the vibrational frequencies, along with the Au–N and N–N force constants and bond orders for the Na[AuCl_4–_ *ₓ*(N_3_)*_x_*]·2H_2_O (*x* = 0–3) and Na[Au(N_3_)_4_]·*n*H_2_O (*n* = 0–2) series, show good agreement with those obtained from quantum chemical calculations. Furthermore, the results align well with previously published data on alkali and alkaline earth metal azidoaurates, *A*[Au(N_3_)_4_] (*A* = K, Rb, Cs, [As(Phen)_4_])^[17-18]^ and Ba[Au(N_3_)_4_]_2_·4H₂O.^[21]^ The slight shifts in the Au–N and N–N stretching frequencies within the series can be attributed to symmetry effects arising from the different spatial orientations of azide moieties.

### **Table S9.** Bond lengths (Å) and force constants (Ncm^–1^) (B3LYP/LANL2DZ) within the Na[AuCl_4–_*_x_*(N_3_)*_x_*] series

**[AuCl(N_3_)_3_]^–^ (S1–S4)**

| Structure | *d*(Au–N) | *f*(Au–N) | *d*(Au–Cl) | *f*(Au–Cl) | *d*(N–N) | *f*(N–N) |
| --- | --- | --- | --- | --- | --- | --- |
| S1 | 2.079 | 2.007 | 2.447 | 1.591 | 1.247 | 9.819 |
| S2 | 2.101 | 1.845 | 2.451 | 1.525 | 1.249 | 9.657 |
| S3 | 2.096 | 1.848 | 2.443 | 1.609 | 1.250 | 9.484 |
| S4 | 2.080 | 1.984 | 2.458 | 1.483 | 1.248 | 9.754 |

**[AuCl_2_(N_3_)_2_]^–^ (S5–S7)**

| Structure | *d*(Au–N) | *f*(Au–N) | *d*(Au–Cl) | *f*(Au–Cl) | *d*(N–N) | *f*(N–N) |
| --- | --- | --- | --- | --- | --- | --- |
| S5 | 2.093 | 1.910 | 2.452 | 1.563 | 1.247 | 9.792 |
| S6 | 2.081 | 1.981 | 2.440 | 1.672 | 1.247 | 9.927 |
| S7 | 2.091 | 1.853 | 2.464 | 1.456 | 1.245 | 9.876 |

**[Au(N_3_)_4_]^–^ (S8–S9)**

| Structure | *d*(Au–N) | *f*(Au–N) | *d*(Au–Cl) | *f*(Au–Cl) | *d*(N–N) | *f*(N–N) |
| --- | --- | --- | --- | --- | --- | --- |
| S8 | 2.096 | 1.876 | -- | -- | 1.247 | 9.895 |
| S9 | 2.091 | 1.872 | -- | -- | 1.246 | 9.961 |

**[AuCl_3_(N_3_)]^–^ (S10)**

| Structure | *d*(Au–N) | *f*(Au–N) | *d*(Au–Cl) | *f*(Au–Cl) | *d*(N–N) | *f*(N–N) |
| --- | --- | --- | --- | --- | --- | --- |
| S10 | 2.083 | 1.953 | 2.436 | 1.652 | 1.250 | 9.477 |

**[AuCl_4_]^–^ (S11)**

| Structure | *d*(Au–N) | *f*(Au–N) | *d*(Au–Cl) | *f*(Au–Cl) | *d*(N–N) | *f*(N–N) |
| --- | --- | --- | --- | --- | --- | --- |
| S11 | -- | -- | 2.431 | 1.557 | -- | -- |

# References

[1] M. Bonamico, G. Dessy, A. Vaciago, Sulla struttura del cloroaurato(III) di sodio biidrato e di analoghi composti, *Atti Accad. Naz. Lincei, Rend. Cl. Sci. Fis. Mat. & Nat.* **1965**, *39*, 504-509, <http://www.bdim.eu/item?id=RLINA_1965_8_39_6_504_0>.

[2] G.M. Sheldrick, A short history of SHELX, *Acta Crystallogr. A* **2008**, *64*, 112-122, <https://doi.org/10.1107/S0108767307043930>

[3] G.M. Sheldrick, Crystal structure refinement with SHELXL, *Acta Crystallogr. C* **2015**, *71*, 3-8, <https://doi.org/10.1107/S2053229614024218>.

[4] V. Petříček, L. Palatinus, J. Plášil, M. Dušek, Jana2020 – a new version of the crystallographic computing system Jana, *Z. Kristallogr.* **2023**, *238*, 271-282, <https://doi.org/10.1515/zkri-2023-0005>.

[5] V. Favre-Nicolin, R. Cerny, FOX, 'free objects for crystallography': a modular approach to ab initio structure determination from powder diffraction, *J. Appl. Crystallogr.* **2002**, *35*, 734-743, <https://doi.org/10.1107/s0021889802015236>.

[6] Crystal Impact - Dr. H. Putz & Dr. K. Brandenburg GbR, *Diamond - Crystal and Molecular Structure Visualization*, Kreuzherrenstr. 102, 53227 Bonn, Germany, **2017**,

[7] F. Neese, The ORCA program system, *WIREs Comput. Mol. Sci.* **2012**, *2*, 73-78, <https://doi.org/10.1002/wcms.81>.

[8] A.D. Becke, Density functional calculations of molecular bond energies, *J. Chem. Phys.* **1986**, *84*, 4524-4529, <https://doi.org/10.1063/1.450025>.

[9] A.D. Becke, Density-functional thermochemistry. III. The role of exact exchange, *J. Chem. Phys.* **1993**, *98*, 5648-5652, <https://doi.org/10.1063/1.464913>.

[10] C.T. Lee, W.T. Yang, R.G. Parr, Development of the Colle-Salvetti correlation-energy formula into a functional of the electron density, *Phys. Rev. B* **1988**, *37*, 785-789, <https://doi.org/10.1103/PhysRevB.37.785>.

[11] S.H. Vosko, L. Wilk, M. Nusair, Accurate spin-dependent electron liquid correlation energies for local spin density calculations: a critical analysis, *Can. J. Phys.* **1980**, *58*, 1200-1211, <https://doi.org/10.1139/p80-159>.

[12] P.J. Stephens, F.J. Devlin, C.F. Chabalowski, M.J. Frisch, *Ab Initio* Calculation of Vibrational Absorption and Circular Dichroism Spectra Using Density Functional Force Fields, *J. Phys. Chem.* **1994**, *98*, 11623-11627, <https://doi.org/10.1021/j100096a001>.

[13] T.H. Dunning, P.J. Hay, Gaussian Basis Sets for Molecular Calculations*,*in *Modern Theoretical Chemistry, Vol 3: Methods of Electronic Structure Theory* (Ed.: H. F. Schaefer), Springer US, Boston, MA, **1977**, pp. 1-27, <https://doi.org/10.1007/978-1-4757-0887-5_1>.

[14] P.J. Hay, W.R. Wadt, *Ab initio* effective core potentials for molecular calculations. Potentials for the transition metal atoms Sc to Hg, *J. Chem. Phys.* **1985**, *82*, 270-283, <https://doi.org/10.1063/1.448799>.

[15] W.R. Wadt, P.J. Hay, *Ab initio* effective core potentials for molecular calculations. Potentials for main group elements Na to Bi, *J. Chem. Phys.* **1985**, *82*, 284-298, <https://doi.org/10.1063/1.448800>.

[16] P.J. Hay, W.R. Wadt, *Ab initio* effective core potentials for molecular calculations. Potentials for K to Au including the outermost core orbitals, *J. Chem. Phys.* **1985**, *82*, 299-310, <https://doi.org/10.1063/1.448975>.

[17] R.W. Williams, S. SchIücker, B.S. Hudson, Inelastic neutron scattering, Raman, vibrational analysis with anharmonic corrections, and scaled quantum mechanical force field for polycrystalline L-alanine, *Chem. Phys.* **2008**, *343*, 1-18, <https://doi.org/10.1016/j.chemphys.2007.09.063>.

[18] R.W. Williams, D. Malhotra, van der Waals corrections to density functional theory calculations: Methane, ethane, ethylene, benzene, formaldehyde, ammonia, water, PBE, and CPMD, *Chem. Phys.* **2006**, *327*, 54-62, <https://doi.org/10.1016/j.chemphys.2006.03.037>.

[19] R.W. Williams, A scaled quantum mechanical force field and vibrational analysis for the gamma glycine crystal polymorph: Hydrogen bond stretching modes observed, *J. Mol. Struct. THEOCHEM* **2004**, *685*, 101-107, <https://doi.org/10.1016/j.theochem.2004.04.044>.

[20] P. Pulay, G. Fogarasi, F. Pang, J.E. Boggs, Systematic ab initio gradient calculation of molecular geometries, force constants, and dipole moment derivatives, *J. Am. Chem. Soc.* **1979**, *101*, 2550-2560, <https://doi.org/10.1021/ja00504a009>.

[21] *Jmol: an open-source Java viewer for chemical structures in 3D*, <http://www.jmol.org/>.

[22] T. Steiner, The Hydrogen Bond in the Solid State, *Angew. Chem. Int. Ed.* **2002**, *41*, 48-76, [https://doi.org/10.1002/1521-3773(20020104)41:1<48::AID-ANIE48>3.0.CO;2-U](https://doi.org/10.1002/1521-3773(20020104)41:1).

[23] H. Siebert, *Anwendungen der Schwingungsspektroskopie in der Anorganischen Chemie, Vol. VII*, Springer-Verlag, Berlin/Heidelberg, **1966**.

[24] J. Weidlein, U. Müller, D. Dehnicke, *Schwingungsspektroskopie. Eine Einführung.*, Georg Thieme Verlag, Stuttgart, **1988**.

[25] O. Reckeweg, A. Simon, Azides and Cyanamides - Similar and Yet Different, *Z. Naturforsch. B* **2003**, *58*, 1097-1104, <https://doi.org/10.1515/znb-2003-1111>.

[26] S. Afyon, P. Höhn, M. Armbrüster, A. Baranov, F.R. Wagner, M. Somer, R. Kniep, Azidoaurates of the Alkali Metals, *Z. Anorg. Allg. Chem.* **2006**, *632*, 1671-1680, <https://doi.org/10.1002/zaac.200600062>.

[27] Y. Subaşı, E.S. Tekin, Y. Prots, F. Jach, M. Somer, S. Afyon, P. Höhn, The First Alkaline-Earth Azidoaurate(III), Ba[Au(N_3_)_4_]_2_ ⋅ 4 H_2_O, *Chem. Eur. J.* **2023**, *29*, e202203501, <https://doi.org/10.1002/chem.202203501>.

# Author Contributions

Peter Höhn, Mehmet Somer and Joannis Psilitelis (in Tübingen, Germany, 1996) grew the single crystals, Yurii Prots performed single crystal X-Ray diffraction experiments, Raul Cardoso performed powder X-ray diffraction experiments, Thomas Doert collected and analyzed high temperature powder X-Ray diffraction data, Peter Höhn, Alexander Ovchinnikov and Yurii Prots performed the crystal structure refinements, Marcus P. Schmidt performed the DSC measurements, and Mehmet Somer, Kamil Kiraz, Peter Höhn and Franziska Jach carried out and interpreted IR and Raman experiments. Ayberk Yılmaz and Helge Rosner performed DFT calculations. Peter Höhn and Mehmet Somer wrote the paper with the help of all authors.
